# Associations between types and sources of dietary carbohydrates and cardiovascular disease risk: a prospective cohort study of UK Biobank participants

**DOI:** 10.1186/s12916-022-02712-7

**Published:** 2023-02-14

**Authors:** Rebecca K. Kelly, Tammy Y. N. Tong, Cody Z. Watling, Andrew Reynolds, Carmen Piernas, Julie A. Schmidt, Keren Papier, Jennifer L. Carter, Timothy J. Key, Aurora Perez-Cornago

**Affiliations:** 1 Cancer Epidemiology Unit, Nuffield Department of Population Health, University of Oxford, Richard Doll Building, Roosevelt Drive, Oxford, OX3 7LF UK; 2 Department of Medicine, University of Otago, Dunedin, 9016 New Zealand; 3 Nuffield Department of Primary Care Health Sciences, University of Oxford, Oxford, OX3 7LF UK; 4 Department of Clinical Epidemiology, Department of Clinical Medicine, Aarhus University and Aarhus University Hospital, Olof Palmes Allé 43-45, 8200 Aarhus N, Denmark; 5 Clinical Trial Service Unit and Epidemiological Studies Unit, University of Oxford, Oxford, OX3 7LF UK

**Keywords:** Coronary heart disease, Stroke, Nutritional epidemiology, Primary prevention, Carbohydrates

## Abstract

**Background:**

Recent studies have reported that the associations between dietary carbohydrates and cardiovascular disease (CVD) may depend on the quality, rather than the quantity, of carbohydrates consumed. This study aimed to assess the associations between types and sources of dietary carbohydrates and CVD incidence. A secondary aim was to examine the associations of carbohydrate intakes with triglycerides within lipoprotein subclasses.

**Methods:**

A total of 110,497 UK Biobank participants with ≥ two (maximum five) 24-h dietary assessments who were free from CVD and diabetes at baseline were included. Multivariable-adjusted Cox regressions were used to estimate risks of incident total CVD (4188 cases), ischaemic heart disease (IHD; 3138) and stroke (1124) by carbohydrate intakes over a median follow-up time of 9.4 years, and the effect of modelled dietary substitutions. The associations of carbohydrate intakes with plasma triglycerides within lipoprotein subclasses as measured by nuclear magnetic resonance (NMR) spectroscopy were examined in 26,095 participants with baseline NMR spectroscopy measurements.

**Results:**

Total carbohydrate intake was not associated with CVD outcomes. Free sugar intake was positively associated with total CVD (HR; 95% CI per 5% of energy, 1.07;1.03–1.10), IHD (1.06;1.02–1.10), and stroke (1.10;1.04–1.17). Fibre intake was inversely associated with total CVD (HR; 95% CI per 5 g/d, 0.96;0.93–0.99). Modelled isoenergetic substitution of 5% of energy from refined grain starch with wholegrain starch was inversely associated with total CVD (0.94;0.91–0.98) and IHD (0.94;0.90–0.98), and substitution of free sugars with non-free sugars was inversely associated with total CVD (0.95;0.92–0.98) and stroke (0.91;0.86–0.97). Free sugar intake was positively associated with triglycerides within all lipoproteins.

**Conclusions:**

Higher free sugar intake was associated with higher CVD incidence and higher triglyceride concentrations within all lipoproteins. Higher fibre intake and replacement of refined grain starch and free sugars with wholegrain starch and non-free sugars, respectively, may be protective for incident CVD.

**Supplementary Information:**

The online version contains supplementary material available at 10.1186/s12916-022-02712-7.

## Background

Cardiovascular disease (CVD) is the leading cause of death worldwide [1]. Evidence from randomised controlled trials (RCTs) and observational studies suggests that total carbohydrate intakes are neither harmful nor beneficial to cardiovascular health [2–4]. However, recent studies suggest that carbohydrate quality may be a more important determinant of CVD outcomes than carbohydrate quantity [3].

Carbohydrates are classified chemically as monosaccharides and disaccharides (sugars), polyols, oligosaccharides, and polysaccharides (starch and non-starch) [2]. Sugars may be further categorised as free sugars (all monosaccharides and disaccharides added to foods by the manufacturer, cook or consumer, plus sugars naturally present in honey, syrups, and unsweetened fruit juices) or non-free sugars (all sugars excluded from the definition for free sugars, mostly naturally occurring in fruit, vegetables, and dairy products) [2, 5]. Public health bodies recommend limiting free sugar [2, 6] or added sugar [7] intake to ~5–10% of total daily energy intake based on meta-analyses of RCTs in adults showing that reducing free sugar intake in an ad libitum diet reduces total energy intake, which may relate to lower body weight [2, 8]. Moreover, in a recent UK Biobank study, we found that free sugar intake was associated with higher triglyceride concentrations [9], which Mendelian randomisation (MR) studies have suggested have a causal association with ischaemic heart disease (IHD) [10, 11], although it is unknown whether these associations depend on the transporting lipoprotein particle. While meta-analyses of observational studies have found that intake of sugar-sweetened beverages (SSBs) is associated with IHD [12–15], the associations between total dietary free sugars and risk of CVD and CVD subtypes remain unclear [2, 16, 17].

Findings from a recent meta-analysis of observational studies suggest that higher intakes of wholegrain foods and fibre are associated with lower risk of IHD, although evidence is limited for stroke risk [18]. Increasing fibre and wholegrain food intake improves cardiometabolic risk markers (e.g. adiposity and blood pressure) in RCTs [18]. Whereas higher risks of total CVD and stroke were observed among participants in the highest category of refined grain food intake in a recent large observational study [19].

The primary aim of this study was to investigate the prospective associations of types and sources of carbohydrates with risks of total CVD, IHD and total stroke, and the role of dietary substitutions in these associations. A secondary aim was to examine the associations of carbohydrate intakes with plasma triglycerides in different lipoprotein subclasses as determined by nuclear magnetic resonance (NMR) spectroscopy.

## Methods

### Study design and participants

UK Biobank is a prospective cohort study of 503,317 men and women aged 37 to 73 years recruited between 2006 and 2010 [20]. Eligible adults living within 25 miles of 22 assessment centres across England, Wales and Scotland (9.2 million) were identified from National Health Service (NHS) registers and invited to participate (response rate 5.5%). At baseline, participants provided detailed information on lifestyle and sociodemographic factors via a self-administered touchscreen questionnaire and interview, and physical measurements and biological samples were collected using standardised procedures (see Additional file 1: Supplemental methods). The UK Biobank was approved by the NHS North West Multicentre Research Ethics Committee (approval letter dated 29th of June 2021, reference 21/NW/0157), and all participants provided informed consent to participate and be followed through linkage to their health records. Further details regarding the study protocol and data access for researchers have been published elsewhere [21].

### Assessment of carbohydrate intakes

Diet was measured using the Oxford WebQ questionnaire, an online 24-h dietary assessment [22]. This questionnaire was recently validated against energy expenditure measured by accelerometery and biomarkers for total sugar intake and found to perform well compared with traditional interviewer-administered 24-h dietary recalls [23]. Participants recruited between April 2009 and September 2010 completed the 24-h dietary assessment at the assessment centre. Participants who provided a valid email address at recruitment were invited to complete identical 24-h dietary assessments on four further occasions between February 2011 and April 2012 (Additional file 1 Fig. S1).

Intakes of 206 food items and 32 beverages were calculated from responses to each 24-h dietary assessment. Carbohydrate intakes were calculated by multiplying the carbohydrate content of food items and beverages by the frequency of intake using the UK Nutrient Databank food composition tables [24]. Types of carbohydrates calculated included total sugars, which were further separated into free sugars and non-free sugars (total sugars minus free sugars) [5], and fibre (non-starch polysaccharides [NSPs] measured using the Englyst method) [24, 25]. Sources of carbohydrates were also calculated as follows: refined grain starch (starch content of white bread, white pasta and rice, other cereals, pizza, samosas, pakoras, grain dishes with added fat, savoury snacks, savoury crackers, biscuits, cakes, pastries and desserts), and wholegrain starch (starch content of brown seeded and wholemeal bread, wholemeal pasta and brown rice, bran cereal, biscuit cereal, oat cereal and muesli) [26]. The starch content of wholegrain and refined grain foods were calculated to approximate the amount of wholegrain and refined grains consumed, as starch is the primary component of wheat grains [27]. Intakes of carbohydrates were calculated from the average of ≥ two (maximum of five) 24-h dietary assessments to minimise the effects of random error and within-person variability [9, 28]. See Additional file 1 Table S1 for further details on the food items and beverages used to calculate carbohydrate types and sources.

### Ascertainment of cardiovascular disease outcomes

Information on date and cause for hospital admission were coded from linkage to Health Episode Statistics for English participants, the Patient Episode Database for Welsh participants, and Scottish Morbidity Records for Scottish participants. Date and cause of death were provided by the NHS Information Centre for English and Welsh participants and NHS Central Register Scotland for Scottish participants’ death certificates. At the time of our analyses, hospital admission data were available up until 30th of September 2021 for England, 31st of July 2021 for Scotland, and 28th of February 2018 for Wales, and death data were available up until 30th of September 2021 for England and Wales, and 31st of October 2021 for Scotland. Therefore, we censored analyses for all outcomes at the earliest censoring date for each country.

Primary outcomes were IHD, defined as a primary diagnosis of incident (fatal or non-fatal) IHD (ICD-10 [international classification of diseases, 10th revision] codes I21-I25) or coronary revascularisation (OPCS-4 [Classification of Interventions and Procedures, 4th revision] codes K49-K50, K75, K40-K46); total stroke, defined as primary diagnosis of incident (fatal or non-fatal) ischaemic or haemorrhagic stroke (ICD-10 codes I60-I61, I63-I64); and total CVD, defined as a primary diagnosis of incidental (fatal or non-fatal) IHD or total stroke (see Additional file 1 Table S2) [29–32]. We performed secondary analyses for IHD and stroke subtypes, including acute myocardial infarction (AMI; ICD-10 I21), ischaemic stroke (ICD-10 I63), and haemorrhagic stroke (ICD-10 I60-I61).

### Measurement of triglycerides in lipoprotein subclasses

Lipids and other metabolic measures (168 absolute levels and 81 ratios) were quantified from a random subset of ~118,000 non-fasting plasma samples obtained from UK Biobank participants at baseline (2006–2010) using high-throughput NMR spectroscopy (Nightingale Health Ltd., Helsinki, Finland) [33]. In a recent UK Biobank study of macronutrient intakes and serum lipids measured by clinical chemistry [9], carbohydrate intakes were most strongly associated with total triglycerides, although it remains unclear whether these associations diverge for triglycerides within different lipoprotein subclasses [10, 34]. The Nightingale NMR platform provided simultaneous quantification of total triglyceride concentrations and triglyceride concentrations within 17 lipoprotein subclasses. Triglyceride measurements with ≥20% of values below the limit of quantification (LOQ) were excluded (*n*=1) and values below the LOQ were set to half the minimum lowest measured value for that triglyceride measurement (Additional file 1 Table S3) [35, 36]. Therefore, total triglyceride concentrations and triglyceride concentrations within 16 lipoprotein subclasses were included in this study. Non-fasting blood collection procedures are described in detail elsewhere [37], and further information on NMR spectroscopy measurements and quality control can be found in Additional file 1 Supplemental methods.

### Exclusion criteria

**Fig. 1:**
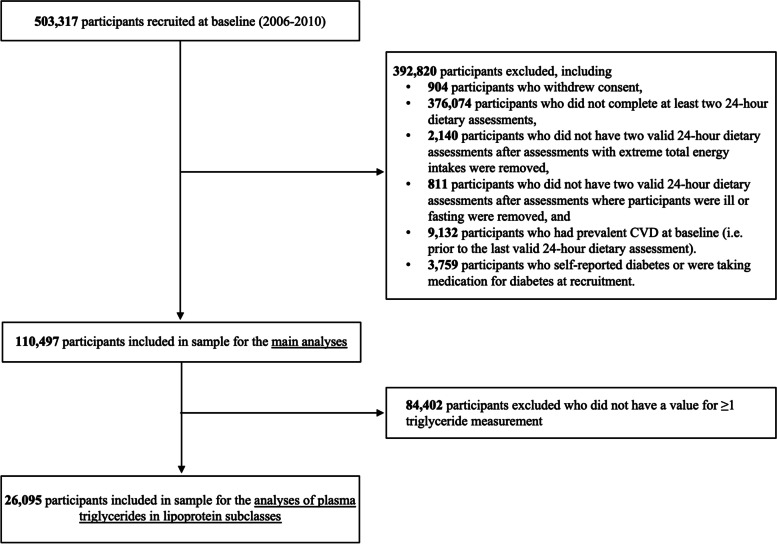
Flow chart of participants included in the sample for the main prospective analyses (*n*=110,497) and the observational analyses of plasma total triglycerides and triglycerides in lipoprotein subclasses (*n*=26,095). *Abbreviations:* CVD cardiovascular disease

Participants were excluded if they withdrew consent from the study (*n*=904), had prevalent CVD prior to their most recent 24-h dietary assessment (*n*=9132), or diabetes at recruitment (either self-reported diabetes diagnosis or were taking medication for diabetes; *n*=3759), or they did not complete ≥ two 24-h dietary assessments (*n*=376,074; see Fig. 1). Participants were also excluded if they did not have ≥ two 24-h dietary assessments after excluding dietary assessments with extreme energy intakes (outside the range of 3347 to 17573 kJ, or 800 to 4200 kcal/d for men, outside the range of 2092 to 14,644 kJ, or 500 to 3500 kcal/d for women [38]; *n*=2140) or where participants reported they were ill or fasting on the day of dietary assessment (*n*=811). The main prospective analyses included a total of 110,497 participants who completed on average 2.9 (SD 0.9) 24-h dietary assessments. For the observational analyses of carbohydrate intakes and triglycerides, participants were further excluded if they were missing values for one or more triglyceride measurements (*n*=84,402), leaving a total of 26,095 participants available for these analyses.

### Statistical analysis

Carbohydrate intakes were expressed as a percentage of total energy intake, except for fibre which was expressed in grams per day (g/d), and each were categorised into quartiles. The baseline characteristics of participants were described by highest and lowest quartiles of total carbohydrate, free sugar, and fibre intakes.

Cox proportional hazards regression, with age as the underlying time variable, was used to estimate hazard ratios (HRs) and 95% confidence intervals (CIs) for the associations between quartiles of carbohydrate intakes and CVD incidence. Carbohydrate intakes were also modelled continuously in increments of 5% higher energy intake except for fibre intake, which was modelled in increments of 5 g/d higher intake. Potential non-linear associations were assessed by using likelihood ratio (LR) tests comparing the model with quartiles of carbohydrates intake treated as ordered categorical variables to a model with quartiles of carbohydrate intakes treated as continuous variables. Tests for linear trend were performed using the continuous (per increment) values for carbohydrate intakes. We tested the proportional hazards assumption on the basis of Schoenfeld residuals, and this was not violated for exposures and covariates of interest in our multivariable models for any outcome.

We estimated participant survival time from age at last completed 24-h dietary assessment until age at last follow-up, first diagnosis of CVD outcome, loss to follow-up or death, whichever occurred first. The minimally adjusted model was stratified by age at recruitment (<45, 45–49, 50–54, 55–59, 60–64, ≥65 years) and sex, and adjusted for recruitment region (London, North West England, North-Eastern England, Yorkshire & the Humber, West Midlands, East Midlands, South-East England, South-West England, Wales & Scotland). Multivariable models were further adjusted for ethnicity (white, mixed, Asian or Asian British, black or black British, other, unknown), Townsend deprivation index (quintiles from least to most deprived, unknown), education (college/university degree or vocational qualification, national examination at 17–18 years of age, national examination at 16 years of age, unknown), alcohol intake (0.1-0.9 g/d, 1–4.9 g/d, 5-14.9 g/d, ≥15 g/d, or none for women, and 0.1–0.9 g/d, 1–4.9 g/d, 5–29.9 g/d, ≥30 g/d, or none for men), smoking status (never, former, light smokers [<15 cigarettes/d], medium to heavy smokers [≥15 cigarettes/d], smoker of unknown number of cigarettes, unknown), physical activity (low, medium or high according to excess metabolic equivalent task [MET] hours/week, unknown), menopausal status at recruitment among women only (pre-menopausal, post-menopausal, unknown), body mass index (BMI; <20, 20–22.49,22.5–24.9, 25.0–27.49, 27.5–29.9, 30–32.49, 32.5–34.9, ≥35 kg/m^2^, unknown), saturated fatty acid (SFA) intake (quintiles of % of energy intake), and average daily energy intake (sex-specific quintiles of kJ/d). Multivariable models were also adjusted for fruit and vegetable intake (quintiles of g/d) as a marker of healthy diet, excepting for models with total sugars, non-free sugars, and fibre because whole fruit and vegetables are a major source of these exposures and therefore introduce collinearity.

We also examined the role of other key cardiometabolic risk factors in supplemental analyses, including waist circumference, systolic blood pressure, serum lipids measured by clinical chemistry (LDL cholesterol [LDL] cholesterol, high-density lipoprotein [HDL] cholesterol, triglycerides, apolipoprotein B [ApoB]), and glycated haemoglobin (HbA1c); however, because these were potential physiological mediators, they were not included in the final models. While we examined other dietary factors (i.e. polyunsaturated fatty acids, monounsaturated fatty acids and trans fatty acids) and women-specific variables (i.e. menopausal hormonal therapy, oral contraceptive pill use, and parity) as potential covariates, these did not have any effects on the model and were therefore not included in the final models. Dietary covariates (i.e. total energy, fruit and vegetable, and SFA intakes) were calculated from responses to ≥ two 24-h dietary assessments (2009–2012), and all other covariates were defined from questionnaire and interview data and physical measurements collected at the UK Biobank assessment centre visit at recruitment (2006–2010). See Additional file 1 Supplemental methods for further details on covariate definitions.

#### Analyses of dietary substitution

In modelled isoenergetic substitution analyses, we estimated the risks of CVD outcomes when 5% of energy from refined grain starch was replaced with wholegrain starch, or 5% of energy from free sugars was replaced with non-free sugars. Models included energy from all other carbohydrates (i.e. energy from total carbohydrates minus energy from free sugars or refined grain starch), energy from protein, energy from fats, and total energy. Therefore, regression coefficients can be interpreted as the estimated effect of replacing refined grain starch or free sugars with wholegrain starch or non-free sugars, respectively [38].

#### Observational analyses of triglycerides in lipoprotein subclasses

For carbohydrate types or sources that were significantly associated with CVD risks in our main analyses, and were also significantly associated with triglycerides measured by clinical chemistry in our prior analyses of UK Biobank [9], we assessed their associations with concentrations of plasma total triglycerides and triglycerides in lipoprotein subclasses in a subsample of participants with baseline NMR spectroscopy measurements (*n*=26,095; see Fig. 1). We calculated the geometric means (with 95% CI) of triglyceride measurements. Multivariable linear regression models adjusted for the same covariates as our main Cox regression models were used to examine the associations between the carbohydrate of interest and each log-transformed triglyceride measurement. We exponentiated the regression coefficients, subtracted one from this number, and multiplied by 100 to obtain the estimated percentage difference in triglyceride concentrations per each higher increment of carbohydrate intake. Further details on metabolite analyses can be found in Additional file 1 Supplemental methods.

#### Sensitivity and subgroup analyses

The robustness of our prospective findings was examined in sensitivity analyses by restricting to participants with (i) ≥ three 24-h dietary assessments (*n*=67,218), and (ii) ≥ 2 years of follow-up (*n*=109,682). We also conducted a sensitivity analysis using absolute intakes of refined grain foods and wholegrain foods in grams per day as exposures. Heterogeneity in associations across subgroups of sex, BMI (~median, <26, ≥26 kg/m^2^), and smoking status (never smoker, ever smoker) was assessed by including an interaction term between the subgroup and exposure of interest in the Cox model and testing for statistical significance using a LR test.

LR *χ*^2^ statistics were obtained by comparing the Cox regression models with and without the exposure of interest (i.e. carbohydrate intakes) as a measure of the extent to which each exposure predicted CVD risks in different models [39]. The percentage change in the LR *χ*^2^ statistic after adjustment for covariates was calculated using the minimally adjusted model as the reference, with large reductions suggesting that part of any remaining associations may be due to residual confounding [39]. All tests of significance were two-sided, and the Benjamini-Hochberg method was used to control the false discovery rate (FDR) with the alpha set to 0.05 to determine *P*-values that survived multiple testing [40]. All analyses were conducted using Stata version 17.0 (Stata Corp, TX, United States), and figures were created using R 4.1.2 (R Core Team, Vienna, Austria).

## Results

### Participant characteristics

Participant characteristics by quartiles of total carbohydrate, free sugar, and fibre intakes are displayed in Table 1 (see Additional file 1 Tables S4-S6 for characteristics across all quartiles). Participants with the highest intakes of total carbohydrate had lower alcohol intakes, total energy intakes, SBP, BMI, and waist circumference, and a higher proportion were women, and a lower proportion were current smokers. Participants with the highest intakes of free sugar had higher total energy intake, waist circumference, and total triglyceride concentrations (measured by clinical chemistry), and a higher proportion were men and current smokers. Whereas the highest consumers of fibre had higher total energy intake, as well as lower BMI, waist circumference, and LDL-C concentrations, and a lower proportion were current smokers. Mean intakes and main food sources of carbohydrate intakes are shown in Additional file 1 Tables S7-S8 and Fig. S2, respectively.

**Table 1:** Baseline characteristics across lowest and highest quartiles total carbohydrate, free sugar, and fibre intakes in 110,497 UK Biobank participants

Characteristics	Total carbohydrate intake		Free sugar intake		Fibre intake	
	Q1	Q4	Q1	Q4	Q1	Q4
*N*	27,625	27,624	27,625	27,624	27,625	27,624
Intake of carbohydrate of interest^a^	39.9 (4.6)	58.3 (3.5)	5.9 (1.7)	17.9 (3.6)	11.3 (2.1)	25.3 (4.1)
Sociodemographic characteristics						
Age at recruitment	55.7 (7.7)	55.6 (8.0)	55.9 (7.6)	55.2 (8.1)	54.6 (7.8)	56.5 (7.8)
Female sex, *n* (%)	15,111 (54.7%)	16,700 (60.5%)	18,388 (66.6%)	13,511 (48.9%)	16,552 (59.9%)	14,537 (52.6%)
White ethnicity, *n* (%)	26,929 (97.8%)	26,313 (95.5%)	26,732 (97.1%)	26,448 (96.1%)	26,423 (96.0%)	26,806 (97.4%)
Most affluent, *n* (%)^b^	5841 (21.1%)	5903 (21.4%)	5895 (21.3%)	5988 (21.7%)	5804 (21.0%)	6055 (21.9%)
College or university degree, *n* (%)	20,760 (79.2%)	20,006 (78.3%)	20,550 (79.1%)	19,740 (76.8%)	18,971 (74.1%)	21,455 (82.3%)
Lifestyle						
Alcohol (g/d)^c^	24.43 (20.73)	9.55 (11.84)	15.67 (15.88)	16.55 (18.90)	18.84 (19.58)	14.24 (15.20)
Current smoker, *n* (%)	2755 (10.0%)	1344 (4.9%)	1801 (6.5%)	2480 (9.0%)	2941 (10.7%)	1292 (4.7%)
Physical activity (excess MET h/wk)	37.0 (38.2)	40.7 (41.7)	38.0 (38.5)	39.3 (42.1)	34.5 (38.4)	44.8 (43.1)
Energy intake (kJ/d)	8782 (2033)	8187 (1851)	8088 (1850)	8895 (2,005)	7387 (1,612)	9831 (1,905)
SFA intake (% energy intake)	12.8 (3.1)	10.0 (2.4)	11.4 (3.1)	11.6 (2.9)	12.2 (3.1)	10.9 (2.7)
Fruit and vegetable intake (g/d)	327.6 (197.6)	453.8 (256.0)	450.5 (253.7)	318.8 (196.7)	221.3 (126.8)	579.0 (256.8)
Medical history						
Statin use, *n* (%)	2573 (9.3%)	2314 (8.4%)	2264 (8.2%)	2476 (9.0%)	2432 (8.8%)	2296 (8.3%)
Post-menopausal, *n* (%)^d^	10,234 (71.8%)	11,316 (72.0%)	12,859 (73.6%)	8698 (69.1%)	10,503 (68.0%)	10,508 (75.7%)
Biological measurements						
BMI (kg/m^2^)	26.9 (4.4)	26.2 (4.4)	26.6 (4.6)	26.5 (4.3)	26.9 (4.5)	25.9 (4.3)
Waist circumference (cm)	89.1 (13.0)	86.3 (12.6)	86.8 (13.0)	88.8 (12.6)	88.4 (13.0)	87.0 (12.6)
SBP (mmHg)	137.2 (18.3)	135.9 (18.5)	135.9 (18.4)	136.7 (18.3)	136.1 (18.3)	136.6 (18.3)
LDL-C (mmol/L)^e^	3.66 (0.83)	3.59 (0.82)	3.62 (0.82)	3.63 (0.82)	3.67 (0.83)	3.57 (0.80)
HDL-C (mmol/L)^e^	1.58 (0.41)	1.45 (0.36)	1.56 (0.39)	1.44 (0.37)	1.52 (0.40)	1.49 (0.37)
Triglycerides (mmol/L)^e^	1.62 (0.96)	1.67 (0.96)	1.55 (0.90)	1.76 (1.02)	1.66 (0.98)	1.63 (0.94)
ApoB (mmol/L)^e^	1.05 (0.23)	1.03 (0.23)	1.04 (0.23)	1.05 (0.23)	1.06 (0.23)	1.03 (0.22)
HbA1c (mmol/mol)	34.7 (4.5)	34.7 (4.2)	34.8 (4.7)	34.7 (4.1)	34.6 (4.1)	34.8 (4.2)

Numbers are means (SD) unless otherwise specified as numbers (%), with % representing the column percentage estimated excluding participants with missing responses

See Additional file 1 Tables S4-S6 for participant characteristics across all quartiles.

*Abbreviations: Apo* apolipoprotein, *BMI* body mass index, *h/wk* hours per week, *HbA1c* glycated haemoglobin, *HDL-C* high-density lipoprotein cholesterol*, kg/m*^*2*^ kilogram per square metre, *kJ* kilojoules, *LDL-C* low-density lipoprotein cholesterol, *MET* metabolic equivalent task, *mmHg* millimetres of mercury, *mmol/L* millimoles per litre, *mmol/mol* millimoles per mol, *Q* quartile, *SBP* systolic blood pressure, *SD* standard deviation, *SFA* saturated fatty acid

^a^ Expressed as a percentage of energy intake except for fibre, which is expressed in grams per day. Ranges of carbohydrate intake within each quartile are shown in Table S7 (Additional file 1)

^b^ Participants categorised in the lowest quintile of the Townsend deprivation index (i.e. least deprived)

^c^ Excluding never drinkers

^d^ In women only

^e^ Measured from serum using standard clinical chemistry assays

### Associations of carbohydrate intakes with cardiovascular disease risk

**Fig. 2:**
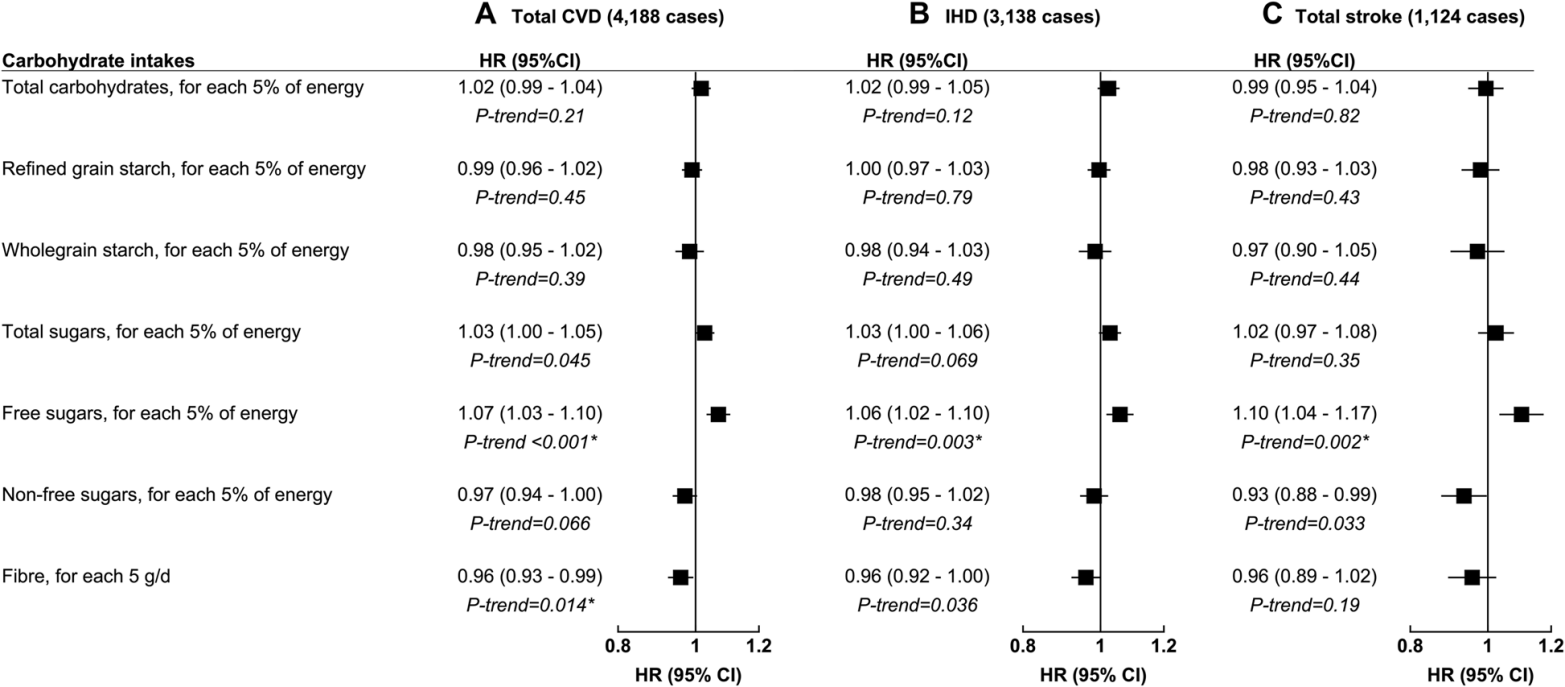
Hazard ratios (95% confidence intervals) for the associations between types and sources of carbohydrates and total CVD (**A**), IHD (**B**), and total stroke (**C**) risk in 110,497 UK Biobank participants. Models were stratified by age at recruitment and sex, and adjusted for recruitment region, ethnicity, Townsend deprivation index, education, alcohol intake, smoking status, physical activity, menopausal status, BMI, SBP, SFA intake, and daily energy intake. Models were also adjusted for fruit and vegetable intake, excepting for models with total sugars, non-free sugars, and fibre as the exposure. Full details regarding each covariate are provided in the statistical analysis section in the main text. *P*-trend values using continuous intakes with asterisks indicating statistical significance after using false discovery rate to correct for multiple testing. *P*-trend values ≥0.1 are displayed to two decimal places and *P*-trend values <0.1 are displayed to three decimal places. *Abbreviations: BMI* body mass index, *CI* confidence interval, *CVD* cardiovascular disease, *g/d* grams per day, *IHD* ischaemic heart disease, *SBP* systolic blood pressure, *SFA* saturated fatty acid

During a median follow-up of 9.4 years, there were 4188, 3138, and 1124 cases of incident total CVD, IHD, and total stroke, respectively. Intake of free sugars was positively associated with total CVD (HR per 5% of energy 1.07; 95% CI 1.03–1.10; *P*-trend<0.001), IHD (1.06; 1.02–1.10; *P*-trend=0.003), and total stroke (1.10; 1.04–1.17; *P*-trend=0.002) risks (Fig. 2). Fibre intake was inversely associated with risk of total CVD (HR per 5 g/d 0.96; 95% CI 0.93–0.99; *P*-trend=0.014). We observed similar directions of association in our analyses of intakes by quartiles, although associations were non-significant for IHD and total CVD in the highest quartile of free sugar intake and fibre intake, respectively (Table 2). Intakes of total carbohydrates, refined grain starch, wholegrain starch, and total sugars were not associated with CVD outcomes. AMI and ischaemic stroke had similar but stronger directions of association with free sugars compared with IHD and total stroke, respectively, whereas no significant associations were found for haemorrhagic stroke (Additional file 1 Table S9). We found no evidence of non-linear associations (FDR-adjusted *P-*values all <0.05). Minimally adjusted models and models with adjustment for key cardiometabolic risk factors are shown in Additional file 1 (Tables S10-S12). In the multivariable model (without adjustment for BMI), the association of free sugars with IHD was attenuated and became non-significant following further adjustment for triglycerides or HDL cholesterol (measured by clinical chemistry), while adjustment for cardiometabolic risk factors did not substantially attenuate the associations of free sugars with total stroke.

**Table 2:** Hazard ratios (95% confidence intervals) for the associations between types and sources of carbohydrates by quartiles and total CVD, IHD, and total stroke risk in 110,497 UK Biobank participants

Carbohydrate intakes	Hazard ratios (95% CI)			
	Q1	Q2	Q3	Q4
*Total CVD*				
Total carbohydrates				
Cases, *n* (%)	1049	1076	1039	1024
HR (95% CI)	Ref	1.07 (0.98–1.17)	1.06 (0.97–1.17)	1.05 (0.95–1.16)
Refined grain starch				
Cases, *n* (%)	1104	1110	1021	953
HR (95% CI)	Ref	1.05 (0.96–1.14)	1.00 (0.92–1.10)	0.99 (0.90–1.08)
Wholegrain starch				
Cases, *n* (%)	1050	1061	1028	1049
HR (95% CI)	Ref	1.03 (0.95–1.13)	1.00 (0.91–1.09)	0.99 (0.91–1.09)
Total sugars				
Cases, *n* (%)	1034	1004	1072	1078
HR (95% CI)	Ref	0.98 (0.89–1.07)	1.04 (0.95–1.14)	1.07 (0.97–1.17)
Free sugars				
Cases, *n* (%)	941	1012	1020	1215
HR (95% CI)	Ref	1.04 (0.95–1.13)	1.00 (0.91–1.09)	1.13 (1.03–1.23)
Non-free sugars				
Cases, *n* (%)	1152	1050	1003	983
HR (95% CI)	Ref	0.95 (0.87–1.04)	0.95 (0.87–1.04)	0.96 (0.87–1.06)
Fibre				
Cases, *n* (%)	1023	1043	1005	1117
HR (95% CI)	Ref	0.99 (0.91–1.09)	0.92 (0.84–1.02)	0.94 (0.85–1.05)
*IHD*				
Total carbohydrates				
Cases, *n* (%)	773	812	788	765
HR (95% CI)	Ref	1.10 (0.99–1.21)	1.11 (0.99–1.23)	1.08 (0.96–1.22)
Refined grain starch				
Cases, *n* (%)	815	814	784	725
HR (95% CI)	Ref	1.03 (0.93–1.14)	1.02 (0.93–1.13)	0.98 (0.88–1.09)
Wholegrain starch				
Cases, *n* (%)	787	794	753	804
HR (95% CI)	Ref	1.04 (0.94–1.15)	0.99 (0.90–1.10)	1.03 (0.93–1.15)
Total sugars				
Cases, *n* (%)	774	754	828	782
HR (95% CI)	Ref	0.99 (0.89–1.10)	1.10 (0.99–1.21)	1.07 (0.96–1.19)
Free sugars				
Cases, *n* (%)	707	749	778	904
HR (95% CI)	Ref	1.01 (0.91–1.12)	0.99 (0.89–1.10)	1.07 (0.97–1.19)
Non-free sugars				
Cases, *n* (%)	882	791	746	719
HR (95% CI)	Ref	0.96 (0.87–1.06)	0.97 (0.87–1.08)	1.00 (0.90–1.12)
Fibre				
Cases, *n* (%)	759	793	752	834
HR (95% CI)	Ref	1.02 (0.92–1.13)	0.93 (0.84–1.04)	0.94 (0.83–1.06)
*Total stroke*				
Total carbohydrates				
Cases, *n* (%)	294	285	268	277
HR (95% CI)	Ref	1.00 (0.85–1.19)	0.95 (0.80–1.14)	0.97 (0.79–1.18)
Refined grain starch				
Cases, *n* (%)	304	313	260	247
HR (95% CI)	Ref	1.10 (0.94–1.29)	0.99 (0.83–1.17)	1.03 (0.86–1.22)
Wholegrain starch				
Cases, *n* (%)	286	284	292	262
HR (95% CI)	Ref	0.99 (0.84–1.17)	1.00 (0.84–1.18)	0.88 (0.74–1.05)
Total sugars				
Cases, *n* (%)	275	270	264	315
HR (95% CI)	Ref	0.96 (0.81–1.13)	0.91 (0.76–1.08)	1.05 (0.88–1.25)
Free sugars				
Cases, *n* (%)	247	283	257	337
HR (95% CI)	Ref	1.15 (0.97–1.36)	1.02 (0.85–1.22)	1.33 (1.12–1.58)
Non-free sugars				
Cases, *n* (%)	293	277	274	280
HR (95% CI)	Ref	0.91 (0.77–1.07)	0.87 (0.73–1.04)	0.84 (0.69–1.01)
Fibre				
Cases, *n* (%)	279	274	269	302
HR (95% CI)	Ref	0.94 (0.79–1.12)	0.89 (0.74–1.07)	0.94 (0.77–1.15)

Models stratified by age at recruitment and sex, and adjusted for recruitment region, ethnicity, Townsend deprivation index, education, alcohol intake, smoking status, physical activity, menopausal status, BMI, SBP, SFA intake, and daily energy intake. Models were also adjusted for fruit and vegetable intake, excepting for models with total sugars, non-free sugars, and fibre as the exposure. Full details for each covariate are provided in the statistical analysis section in the main text

*Abbreviations: BMI* body mass index, *CI* confidence interval, *CVD* cardiovascular disease, *HR* hazard ratio, *Q* quartile, *Ref* reference, *SBP* systolic blood pressure, *SFA* saturated fatty acid

### Analyses of dietary substitution

Modelled isoenergetic replacement of 5% of energy from refined grain starch with wholegrain starch was associated with lower risks of total CVD (0.94; 0.91–0.98; *P*-trend=0.003) and IHD (0.94; 0.90–0.98; *P*-trend=0.006) (Table 3). Replacement of 5% of energy from free sugars with non-free sugars was associated with lower risks of total CVD (0.95; 0.92–0.98; *P*-trend=0.001) and total stroke (0.91; 0.86–0.97; *P*-trend=0.005).

**Table 3:** Hazard ratios (95% confidence intervals) for the associations between each isoenergetic replacement of 5% of energy from free sugars or refined grains with other carbohydrates and incidence of total CVD, IHD, and total stroke in 110,497 UK Biobank participants

Isoenergetic substitution model^**a**^	Total CVD (4188 cases)		IHD (3138 cases)		Total stroke (1124 cases)	
	HR (95% CI)	***P***-trend^**b**^	HR (95%CI)	***P***-trend^**b**^	HR (95%CI)	***P***-trend^**b**^
Substituting 5% of energy from refined grain starch for 5% of energy from wholegrain starch	0.94 (0.91–0.98)	0.003*	0.94 (0.90–0.98)	0.006*	0.94 (0.87–1.01)	0.11
Substituting 5% of energy from free sugars for 5% of energy from non-free sugars	0.95 (0.92–0.98)	0.001*	0.96 (0.92–0.99)	0.023	0.91 (0.86–0.97)	0.005*

* *P*-trend statistically significant after using false discovery rate to correct for multiple testing

^a^ Isoenergetic substitution models included the same adjustments as per Fig. 2 excluding intake of SFA intake and fruit and vegetable intake, and were further adjusted for energy from all macronutrients excluding the carbohydrate type or source being replaced. Full details for each covariate are provided in the statistical analysis section in the main text

^b^ *P*-trend using continuous intakes. *P*-trend values ≥0.1 are displayed to two decimal places and *P*-trend values <0.1 are displayed to three decimal places

*Abbreviations: CI* confidence interval, *CVD* cardiovascular disease, *HR* hazard ration, *IHD* ischaemic heart disease, *SFA* saturated fatty acid

### Sensitivity and subgroup analyses

Our findings remained similar after restricting to participants with ≥ three 24-h dietary assessments and ≥ 2 years of follow-up, as well as in sensitivity analyses using absolute intakes of refined grain foods and wholegrain foods as the exposure (Additional file 1 Tables S13-S15). No significant heterogeneity by sex, BMI, and smoking subgroups for associations between carbohydrate intakes and cardiovascular outcomes was observed (Additional file 1 Tables S16-S18).

### Associations of carbohydrate intakes with triglycerides in lipoprotein subclasses

Free sugars were most strongly associated with CVD outcomes in our study and were also associated with total triglycerides measured by clinical chemistry in our previous analyses [9]; therefore, we examined the associations of free sugars with plasma triglycerides within lipoprotein subclasses in a subsample of participants with NMR spectroscopy measurements (*n*=26,095). There was a high correlation for log-transformed total triglyceride concentrations measured by clinical chemistry and NMR spectroscopy (*r*=0.94). Free sugar intake was positively associated with total triglycerides (percentage difference in triglyceride concentration per 5% of energy intake 3.04; 95% CI 2.53–3.55) and triglycerides within all lipoprotein subclasses; the strongest associations were observed for triglycerides in chylomicrons and extremely large very low-density lipoprotein (VLDL; 10.12; 7.51–12.79) and very large VLDL (7.36; 6.11–8.62) (Table 4, see Additional file 1 Table S19 for minimally adjusted analyses). In sensitivity analyses restricted to participants fasting for ≥ 4 h at blood collection (*n*=11,076) and participants with all triglyceride measurements above the LOQ (*n*=21,865) the directions of association remained similar (Additional file 1 Tables S20-S21).

**Table 4:** Associations for each 5% of energy from free sugars and concentrations of total triglycerides and triglycerides in lipoprotein subclasses in 26,095 UK Biobank participants

Triglycerides	Geometric mean (95% CI), mmol/L	Percentage difference mean concentrations (95% CI)
Total triglycerides	1.142 (1.136, 1.148)	3.04 (2.53, 3.55)
Triglycerides in VLDL	0.760 (0.755, 0.765)	3.77 (3.14, 4.40)
Triglycerides in chylomicrons and extremely large VLDL	0.058 (0.057, 0.060)	10.12 (7.51, 12.79)
Triglycerides in very large VLDL	0.073 (0.073, 0.074)	7.36 (6.11, 8.62)
Triglycerides in large VLDL	0.135 (0.134, 0.136)	3.94 (3.21, 4.68)
Triglycerides in medium VLDL	0.241 (0.240, 0.243)	2.61 (2.08, 3.14)
Triglycerides in small VLDL	0.141 (0.140, 0.142)	2.50 (2.03, 2.97)
Triglycerides in very small VLDL	0.064 (0.064, 0.064)	1.86 (1.49, 2.24)
Triglycerides in IDL	0.095 (0.094, 0.095)	1.32 (1.02, 1.63)
Triglycerides in LDL	0.136 (0.136, 0.137)	1.45 (1.12, 1.77)
Triglycerides in large LDL	0.092 (0.092, 0.092)	1.27 (0.97, 1.58)
Triglycerides in medium LDL	0.030 (0.030, 0.030)	1.65 (1.29, 2.00)
Triglycerides in small LDL	0.013 (0.013, 0.013)	2.14 (1.74, 2.55)
Triglycerides in HDL	0.134 (0.133, 0.134)	2.21 (1.80, 2.62)
Triglycerides in large HDL	0.028 (0.028, 0.028)	1.52 (1.01, 2.03)
Triglycerides in medium HDL	0.049 (0.049, 0.050)	2.44 (1.98, 2.89)
Triglycerides in small HDL	0.048 (0.047, 0.048)	2.70 (2.28, 3.12)

Multivariable linear regression models with free sugar intake as an independent variable and triglyceride measurements as dependent variables were adjusted for age at recruitment, sex, recruitment region, ethnicity, Townsend deprivation index, education, alcohol intake, smoking status, physical activity, menopausal status, SBP, BMI, fruit and vegetable intake, SFA intake, daily energy intake, and fasting status. Full details for each covariate are provided in the statistical analysis section in the main text. Results are expressed as the percentage difference (95% CI) in triglyceride concentrations per 5% higher energy intake from free sugars and were calculated as follows: (e^β^−1)*100

*P*-trend values calculated using continuous intakes were all significant after using false discovery rate to correct for multiple testing. *P*-trend values ≥0.1 are displayed to two decimal places and *P*-trend values <0.1 are displayed to three decimal places

*Abbreviations: BMI* body mass index, *CI* confidence intervals, *CVD* cardiovascular disease, *HDL* high-density lipoprotein, *IDL* intermediate-density lipoprotein, *LDL* low-density lipoprotein cholesterol, *SBP* systolic blood pressure, *SFA* saturated fatty acid, *VLDL* very low-density lipoprotein cholesterol

## Discussion

In this large UK study, higher free sugar intake was significantly positively associated with risks of incident total CVD, IHD, and total stroke, while higher fibre intake was inversely associated with total CVD. Modelled replacement of refined grain starch with wholegrain starch was associated with lower risks of total CVD and IHD, and replacement of free sugars with non-free sugars was associated with lower risks of total CVD and total stroke. Moreover, higher free sugar intake was associated with higher concentrations of total triglycerides and triglycerides within all lipoprotein subclasses.

Few large observational studies of dietary carbohydrates and CVD risk have examined the types and sources of total carbohydrates in detail [2]. This study found no association between total carbohydrate intake and risk of CVD, which is consistent with most previous prospective studies [2, 3]. The findings of our study suggest that specific types of carbohydrate, particularly different sugars, may have diverging associations with CVD risk; we found that intake of free sugars was positively associated with total CVD and all CVD subtypes except for haemorrhagic stroke, while intake of non-free sugars was not associated with CVD outcomes. To the best of our knowledge, no prior study has examined the associations of free sugars, based on the definition revised in 2015 by the World Health Organization [6] and the UK Scientific Advisory Committee on Nutrition [2], with CVD risks, as most previous studies have only looked at added sugars or sucrose as a proxy for free sugars [16, 17]. In 2016, a meta-analysis of observational studies found that added sugars, all of which are free sugars but exclude sugars in juiced or pureed fruit and vegetables, were not associated with total CVD mortality; however, data were not available to assess the associations of added sugars and incident total CVD, and CVD subtypes were not examined separately [16]. SSBs are rich in free sugars, and a recent meta-analysis of 7 prospective cohort studies found that each one serving/d of SSBs was associated with significantly higher risks of total CVD (RR 1.08; 95% CI 1.02–1.14) and IHD (1.15; 1.09–1.22) but not total stroke (1.05; 0.95–1.16), although in subgroup analyses SSB intake was significantly associated with ischaemic stroke risk among women (1.33; 1.07–1.66) [15].

Free sugars were most strongly associated with total stroke risk in our whole cohort analyses; however, a majority of participants were women (58%), and although we observed no significant heterogeneity by sex in our subgroup analyses, we found that risk estimates for free sugars and total stroke tended to be larger for women (HR 1.15; 95% CI 1.05–1.26) compared with men (1.06; 95% CI 0.98–1.16). Moreover, SSBs were an important source of free sugar intake in our sample (11.4% of free sugar intake), which may partly account for our findings, although fruit juice, which has previously been found to have neutral or inverse associations with CVD risk, was a larger source of free sugars in this sample (15.9% of free sugar intake) [41]. It is possible that specific food sources of free sugars have diverging associations with CVD risk, but we were unable to examine major sources of free sugars separately due to the high number of participants who did not report consuming SSBs and fruit juice across completed 24-h dietary assessments. Further, we found that statistically modelled replacement of free sugars with non-free sugars was associated with lower risks of total CVD and total stroke, which has not been demonstrated previously. Prior observational evidence suggests that fruit, vegetables, and dairy products, which are major dietary sources of non-free sugars, are inversely associated with CVD risk [42–44], and that this may partly explain the observed beneficial association with modelled substitution of free sugars for non-free sugars, although we observed no significant inverse associations of non-free sugars in our main analyses.

Previous RCTs have demonstrated that reducing free sugar intake reduces total energy intake [2, 8], which may relate to lower body weight, and adiposity is an established risk factor for IHD and stroke [45]. However, adding BMI to our main multivariable models did not attenuate the observed associations between free sugars and incident CVD. There is limited evidence for the association of free sugars with other cardiometabolic risk factors (i.e. elevated blood pressure, and fasting glucose) [8, 46–48], although we observed that associations of free sugars with total CVD and IHD attenuated most after adjustment for serum triglycerides and HDL cholesterol. Our recent study in the UK Biobank found that each 5% higher energy from free sugars was associated with higher triglycerides (+0.15 mmol/l) and lower HDL cholesterol (−0.07 mmol/L) [9]. Moreover, MR studies support a causal effect of triglycerides on IHD risk [49, 50], but have strongly suggested that HDL cholesterol is not causal [49, 51], and it is possible that associations of triglycerides vary by the type of transporting lipoprotein particle [10]. For each 5% higher energy from free sugars we observed modestly higher concentrations of log-transformed total triglycerides (+3.04%), with the highest concentrations observed for triglycerides within larger VLDL particles (+10.12%). While many observational and genetic studies have suggested that VLDL particles are associated with higher risk of ischaemic heart disease [52–55], a recent study found that a large proportion of myocardial infarction risk related to apolipoprotein B-containing particles was explained by VLDL cholesterol, but not VLDL triglycerides [56]. Thus, it is possible that the observed associations between free sugars and triglycerides in apolipoprotein B-containing particles are related to the cholesterol rather than triglyceride content of these particles. Moreover, evidence from observational and genetic studies does not support a causal association of triglycerides with total stroke risk [49, 50, 57]. This suggests that triglycerides may not explain the higher risks of total stroke observed with higher free sugar intakes in our study. Our findings for free sugars and stroke attenuated minimally after adjustment for other cardiometabolic risk factors (e.g. adiposity and elevated blood pressure) [58]; further research is warranted to examine the plausible mechanisms for this association.

Starch from refined grains and wholegrains were not associated with CVD incidence in our study [59–62]. In contrast, previous studies have found that wholegrain food intake is associated with lower risks of IHD and stroke [18, 63], and while overall evidence is limited for refined grain foods, the Prospective Urban and Rural Epidemiology study including 137,000 individuals from five low- and middle-income countries recently reported that intakes of ≥350 g/d of refined grain foods were positively associated with major CVD events [19]. However, we observed that modelled replacement of refined grain starch with wholegrain starch was associated with lower risks of IHD and total CVD. Meta-analyses of RCTs show that substitution of refined grain foods with wholegrain foods lowers total cholesterol, LDL cholesterol, and HbA1c [64], which may explain the lower risks of IHD and CVD observed for our modelled substitution analyses [49, 50, 65]. Our study separates the starch in refined grain foods from other macronutrients, such as SFAs and free sugars, which have been found to have harmful associations with CVD risk, to better approximate the amount of wholegrain and refined grain consumed [27, 66]. It is possible that the higher dietary fibre content or potentially the mineral, vitamin, and phytochemical content of wholegrains may still account for some of the beneficial associations observed in this modelled substitution [63].

Lastly, our study confirms the established inverse association between dietary fibre and risk of total CVD [18, 67]. Specifically, we found a 4% lower risk of total CVD for each 5 g/d higher fibre intake, compared to a 9% lower risk of total CVD for each 7 g/d higher fibre intake reported in a recent meta-analysis [18]. However, associations of fibre intake with total CVD in analyses by fourths of intakes attenuated and became non-significant after adjustment for BMI, as well as after adjustment for LDL cholesterol or ApoB measured by clinical chemistry. Meta-analyses of RCTs have found that higher fibre intake lowers body weight and serum LDL cholesterol concentrations [18, 68], which may partly explain the beneficial associations observed for each 5 g/d higher fibre intake in this study. Moreover, highest consumers of fibre in our study (≥21 g/d) had lower mean BMI, waist circumference, and LDL cholesterol.

### Strengths and limitations

Strengths of this study include the large cohort size, prospective study design, and detailed dietary information that allowed the determination of several carbohydrate types and sources. Further, triglycerides measured by NMR spectroscopy at baseline were available for a large subsample of participants (*n*=26,095, 24%), allowing us to examine the novel associations of free sugars and triglycerides in different lipoprotein subclasses.

There are some limitations of the present study to consider. All self-reported dietary assessment techniques are prone to error; however, we have used the average of at least two (maximum 5) 24-h dietary assessments per participant and removed implausible intakes. In a recent validation study, correlations between the mean of two Oxford WebQ’s and estimated true intakes of total sugar and total energy were 0.40 and 0.38, respectively, and improved with further administrations [23]. Moreover, our findings remained similar in sensitivity analyses restricted to participants with ≥ three 24-h dietary assessments, and estimates for total carbohydrates, total sugars, free sugars, and fibre intakes were comparable with those observed for adults aged 19–64 years in the National Diet and Nutrition Survey (2017–2018) [69]. We were unable to account for changes in dietary intakes during the study follow-up period, which potentially increased random error and could have biased associations towards the null. Reverse causality is possible, although we removed individuals with CVD and diabetes at baseline and the findings of our study remained similar after excluding the first 2 years of follow-up. The nature of our analyses of free sugars and triglyceride measurements did not allow us to consider temporality because blood samples were taken several months prior to the completion of most 24-h dietary assessments. Further, our interpretation of sensitivity analyses for stroke was limited by the smaller number of cases after further exclusions. Residual confounding may have influenced our findings, given the moderate to large reductions in *χ*^2^ values after adjustment for some cardiometabolic risk factors in our models for total CVD and IHD, and, as with all observational studies, causality cannot be inferred. Lastly, participants recruited to the UK Biobank are mostly of white European ancestry and are typically healthier than the overall population [70], so our findings may not be generalisable to other populations.

## Conclusions

In summary, we found that associations between carbohydrate intakes and CVD may depend on the type and source of carbohydrate consumed, particularly for sugars. Free sugar intake was associated with higher risks of total CVD and CVD subtypes, particularly total stroke, which supports the global dietary recommendation to consume less than 5% of total energy from free sugars [6]. Free sugar intake was positively associated with triglycerides within all lipoprotein subclasses, which may partly explain the observed higher risk of IHD, while mechanisms for higher total stroke risk remain unclear. Higher fibre intake was associated with lower risks of total CVD, and replacement of refined grain starch and free sugars with wholegrain starch and non-free sugars, respectively, may be protective for CVD. Our findings support the importance of the type and source of carbohydrate consumed for cardiovascular health.

## Supplementary Information

**Additional file 1: Supplemental methods.** Assessment of covariates; Measurement and analyses of triglycerides in lipoprotein classes. **Table S1.** Description of types and sources of dietary carbohydrates. **Table S2.** Outcome definitions for incident CVD and exclusion criteria for prevalent CVD and diabetes. **Table S3.** Triglyceride concentrations measured by NMR spectroscopy flagged as being below the limit of quantification in 26,095 UK Biobank participants. **Table S4.** Baseline characteristics across quartiles of total carbohydrate intake in 110,497 UK Biobank participants. **Table S5.** Baseline characteristics across quartiles of free sugar intake in 110,497 UK Biobank participants. **Table S6.** Baseline characteristics across quartiles of fibre intake in 110,497 UK Biobank participants. **Table S7.** Carbohydrate intakes in grams per day and percentage of energy intake by quartiles of carbohydrate intakes. **Table S8.** Types and sources of carbohydrates in grams per day and percentage of energy intake by quartiles of total carbohydrate intake. **Table S9.** Hazard ratios (95% confidence intervals) for the associations between types and sources of carbohydrates and acute myocardial infarction, ischaemic stroke, and haemorrhagic stroke risk in 110,497 UK Biobank participants. **Table S10.** Hazard ratios (95% confidence intervals) for the associations between carbohydrate intakes and incidence of total CVD in 110,497 UK Biobank participants with adjustment for key cardiometabolic risk factors. **Table S11.** Hazard ratios (95% confidence intervals) for the associations between carbohydrate intakes and incidence of IHD in 110,497 UK Biobank participants with adjustment for key cardiometabolic risk factors. **Table S12.** Hazard ratios (95% confidence intervals) for the associations between carbohydrate intakes and incidence of total stroke in 110,497 UK Biobank participants with adjustment for key cardiometabolic risk factors. **Table S13.** Hazard ratios (95% confidence intervals) for the associations between carbohydrate intakes and total CVD, IHD and total stroke risk in sensitivity analyses restricting to participants with ≥ three 24-h dietary assessments (n=67,218). **Table S14.** Hazard ratios (95% confidence intervals) for the associations between carbohydrate intakes and total CVD, IHD and total stroke risk in sensitivity analyses restricting to participants with ≥ two years of follow-up (n=109,682). **Table S15.** Hazard ratios (95% confidence intervals) for the associations between intake of refined grain foods and wholegrain foods in grams and total CVD, IHD and total stroke risk (n=110,497). **Table S16.** Hazard ratios (95% confidence intervals) for the associations between types of carbohydrate and total CVD, IHD and total stroke risk in 110,497 UK Biobank participants by sex subgroups. **Table S17.** Hazard ratios (95% confidence intervals) for the associations between types of carbohydrate and total CVD, IHD and total stroke risk in 110,497 UK Biobank participants by BMI subgroups. **Table S18.** Hazard ratios (95% confidence intervals) for the associations between types of carbohydrate and total CVD, IHD and total stroke risk in 110,497 UK Biobank participants by smoking status subgroups. **Table S19.** Associations between each 5% of energy from free sugars and concentrations of total triglycerides and triglycerides in lipoprotein subclasses in minimally adjusted linear regression models in 26,095 UK Biobank participants. **Table S20.** Associations between each 5% of energy from free sugars and concentrations of total triglycerides and triglycerides in lipoprotein subclasses in sensitivity analyses restricting to participants fasting for ≥ 4 h prior to serum collection (n=11,076). **Table S21.** Associations between each 5% of energy from free sugars and concentrations of total triglycerides and triglycerides in lipoprotein subclasses in sensitivity analyses restricting to participants with all triglyceride measurements above the limit of quantification (n=21,865). **Fig. S1** Dietary assessments in subsample of 110,497 UK Biobank participants included in our main analyses. **Fig. S2** Top five food contributors to total carbohydrates and types of carbohydrates in 110,497 UK Biobank participants.

## Data Availability

Bona fide researchers can apply to use the UK Biobank dataset by registering and applying at http://ukbiobank.ac.uk/register-apply/.

## Abbreviations

AMI: Acute myocardial infarction
Apo: Apolipoprotein
BMI: Body mass index
CI: Confidence interval
CVD: Cardiovascular disease
g/d: Grams per day
HbA1c: Glycated haemoglobin
HDL: High-density lipoprotein
HR: Hazard ratio
ICD: International Classification of Diseases
IDL: Intermediate-density lipoprotein
IHD: Ischaemic heart disease
LDL: Low-density lipoprotein
LOQ: Limit of quantification
LR: Likelihood ratio
MET: Metabolic equivalent of task
NHS: National Health Service
NMR: Nuclear magnetic resonance
NSP: Non-starch polysaccharide
OPCS: Classification of Interventions and Procedures
Q: Quartile
RCT: Randomised controlled trial
ref: Reference
SBP: Systolic blood pressure
SD: Standard deviation
SFA: Saturated fatty acid
SSB: Sugar-sweetened beverage
VLDL: Very low-density lipoprotein
