# Supplementary material for: Associations between types and sources of dietary carbohydrates and cardiovascular disease risk: a prospective cohort study of UK Biobank participants
Source: BMC Med. 2023 Feb 14;21:34. doi: 10.1186/s12916-022-02712-7 (PMC9926727; doi:10.1186/s12916-022-02712-7)
Supplement: Supplementary file 1 — Additional file 1: Supplemental methods. Assessment of covariates; Measurement and analyses of triglycerides in lipoprotein classes. Table S1. Description of types and sources of dietary carbohydrates. Table S2. Outcome definitions for incident CVD and exclusion criteria for prevalent CVD and diabetes. Table S3. Triglyceride concentrations measured by NMR spectroscopy flagged as being below the limit of quantification in 26,095 UK Biobank participants. Table S4. Baseline characteristics across quartiles of total carbohydrate intake in 110,497 UK Biobank participants. Table S5. Baseline characteristics across quartiles of free sugar intake in 110,497 UK Biobank participants. Table S6. Baseline characteristics across quartiles of fibre intake in 110,497 UK Biobank participants. Table S7. Carbohydrate intakes in grams per day and percentage of energy intake by quartiles of carbohydrate intakes. Table S8. Types and sources of carbohydrates in grams per day and percentage of energy intake by quartiles of total carbohydrate intake. Table S9. Hazard ratios (95% confidence intervals) for the associations between types and sources of carbohydrates and acute myocardial infarction, ischaemic stroke, and haemorrhagic stroke risk in 110,497 UK Biobank participants. Table S10. Hazard ratios (95% confidence intervals) for the associations between carbohydrate intakes and incidence of total CVD in 110,497 UK Biobank participants with adjustment for key cardiometabolic risk factors. Table S11. Hazard ratios (95% confidence intervals) for the associations between carbohydrate intakes and incidence of IHD in 110,497 UK Biobank participants with adjustment for key cardiometabolic risk factors. Table S12. Hazard ratios (95% confidence intervals) for the associations between carbohydrate intakes and incidence of total stroke in 110,497 UK Biobank participants with adjustment for key cardiometabolic risk factors. Table S13. Hazard ratios (95% confidence intervals) for the associations bet [file 12916_2022_2712_MOESM1_ESM.docx]

**Additional File 1: Associations between types and sources of carbohydrates and cardiovascular disease risk: a prospective cohort study of UK Biobank participants**

Rebecca K. Kelly^1^, Tammy Y.N. Tong^1^, Cody Z. Watling^1^, Andrew Reynolds^2^, Carmen Piernas^3^, Julie A Schmidt^1,4^, Keren Papier^1^, Jennifer L Carter^5^, Timothy J Key^1^, Aurora Perez-Cornago^1^

^1^ Cancer Epidemiology Unit, Nuffield Department of Population Health, University of Oxford, Oxford, UK

^2^ Department of Medicine, University of Otago, Dunedin, 9016, New Zealand.

^3^ Nuffield Department of Primary Care Health Sciences, University of Oxford, Oxford, UK.

^4^ Department of Clinical Epidemiology, Department of Clinical Medicine, Aarhus University and Aarhus University Hospital, Olof Palmes Allé 43-45, 8200, Aarhus N, Denmark

^5^ Clinical Trial Service Unit and Epidemiological Studies Unit, Nuffield Department of Population Health, University of Oxford, Oxford, UK.

[Additional File 1: Supplemental methods 3](#_Toc118435723)

[Additional File 1: Tables 10](#_Toc118435724)

[Additional File 1: Figures 47](#_Toc118435725)

## Additional File 1: Supplemental methods

*Assessment of covariates*

Dietary covariates (i.e. total energy intake, fruit and vegetable intake, saturated fatty acid intake) were calculated from responses to ≥ two 24-hour dietary assessments. All other covariates were defined from questionnaire and interview data and physical measurements collected at the UK Biobank assessment centre visit at recruitment (2006-2010). For all covariates, participants were included in an ‘unknown’ category if the covariate was unknown or their response to the question was ‘do not know’ or ‘prefer not to answer’.

Age at recruitment

Age at recruitment was calculated using participants’ month and year of birth, which was verified or updated by participants. Day of birth was nominally assigned as 15 for each participant and age at recruitment was categorised as <45 years, 45-49 years, 50-54 years, 55-59 years, 60-64 years or ≥65 years.

Region of recruitment

The assessment centre recording for each participant was used to code region of recruitment as follows: London, North-West England, North-Eastern England, Yorkshire & the Humber, West Midlands, East Midlands, South-East England, South-West England, Wales, and Scotland. Specifically, assessments centres were assigned the following region: London (assessment centres: St Bartholomew’s Hospital, Hounslow, Croydon), North-West England (assessment centres: Stockport, Manchester, Liverpool, Bury), North-Eastern England (assessment centres: Newcastle, Middlesbrough), Yorkshire & the Humber (assessment centres: Leeds, Sheffield), West Midlands (assessment centres: Stoke, Birmingham) East Midlands (assessment centre: Nottingham), South-East England (assessment centres: Oxford, Reading), South-West England (assessment centre: Bristol), Wales (assessment centres: Swansea, Wrexham, Cardiff), and Scotland (assessment centres: Glasgow, Edinburgh).

Ethnicity

Participants were asked the questions ‘What is your ethnicity?’ and ‘What is your ethnic background?’ and answers were used to classify participants as white (British, Irish, Any other white background), mixed (white and Black Caribbean, white and Black African, white and Asian, Any other mixed background), Asian or Asian British (Indian, Pakistani, Bangladeshi, any other Asian background), black or black British (Caribbean, African, any other Black background), other (other ethnic group), or unknown.

Townsend deprivation index

Townsend deprivation index was calculated by assigning each participant a score corresponding to the output area from the preceding national census in which their postcode was located [1]. This was converted to quintiles to be used as an indicator of socioeconomic status in our analyses, with least deprived (quintile 1) to most deprived (quintile 5), or unknown.

Education

Participant were asked ‘which of the following qualifications do you have?’ at recruitment, which was used to classify participants highest level of education as follows: college or university degree/vocation (college or university degree, National Vocational Qualification or Higher National Diploma or Higher National Certificate or equivalent, or other professional qualifications), national examination at 17-18 years of age (A levels/AS levels or equivalent), national examination at 16 years of age (O levels/ General Certificate of Secondary Education (GCSEs) or equivalent, or Certificate of Secondary Education (CSEs) or equivalent), or unknown (including participants who answered ‘none of the above’).

Alcohol intake

Participants were asked the question ‘about how often do you drink alcohol?’ with possible responses as follows: ‘daily or almost daily’, ‘three or four times a week’, ‘once or twice a week’, ‘one to three times a month’, ‘special occasions only’, ‘never’, and ‘prefer not to answer’. Participants were also asked about their average weekly and monthly intake of different alcoholic beverages: beer or cider, champagne or white wine, fortified wine, red wine, spirits, and other alcoholic beverages. Participants were assigned 10 grams of alcohol per serving for all types of alcohol except for beer and cider, which were assigned 20 grams of alcohol per serving. Alcohol intake in grams per day (g/d) was calculated using responses to these questions, and participants were categorised based on sex as follows; 0.1-0.9 g/d, 1-4.9 g/d, 5-14.9 g/d, ≥15 g/d, or none for women, and 0.1-0.9 g/d, 1-4.9 g/d, 5-29.9 g/d, ≥30 g/d, or none for men. Participants who reported ‘never’ consuming alcohol were assigned to the ‘none’ category and participants who reported consuming alcohol on ‘special occasions only’ were assigned to the ‘0.1-0.9 g/d’ category.

Smoking status

Self-reported smoking status was used to classify participants as never smokers, former smokers, current smokers, or unknown. Current smokers were further classified according to the number of cigarettes they smoke on average each day as follows; light smokers (<15 cigarettes/d), medium to heavy smokers (≥15 cigarettes/d), smoker of unknown number of cigarettes, or unknown.

Physical activity

Participant’s answers to questions about daily walking, moderate physical activity and vigorous physical activity were used to estimate excess metabolic equivalents (METs) per week [2]. Excess METs refer to energy expenditure greater than that of an inactive person. Participants excess METs per week were used to categorise physical activity as low (<10 excess METs/wk), medium (10-49.9 excess METs/wk), high (≥50 excess METs/wk), or unknown.

Menopausal status

Participants were asked about their menopausal status at baseline with the question ‘Have you had your menopause (periods stopped)?’ with response options including, ‘yes’, ‘no’, ‘not sure had hysterectomy’, ‘not sure other reason’ or ‘prefer not to answer’. If participants responded with ‘not sure had hysterectomy’, ‘not sure other reason’ or ‘prefer not to answer’, information from other questions on bilateral oophorectomy, use of menopausal hormonal therapy (MHT), and age at recruitment was used to categorise participants. Participants were then categorised as premenopausal if they reported not having their menopause, were younger than 45 or did not report a bilateral oophorectomy. Participants were categorised as postmenopausal if they reported their periods had stopped, were over the age of 53, had a bilateral oophorectomy, or reported MHT use. Participants were categorised as unknown if they reported not having their period but having had a bilateral oophorectomy or having used MHT, or if they had missing information on menopausal status and could not be reassigned using any additional information.

Body Mass Index

All anthropometry measurements were determined by trained research staff. The SECA 240 height measure was used to measure participants standing height without shoes and to the closest centimetre (cm). Standard scales or the Tanita BC 418 body composition analyser was used to measure participant weight without shoes and outdoor clothing. If participants underwent bioimpedance analysis this value was used for weight instead of the measurement using standard scales. Body Mass Index (BMI) was calculated as weight (kg)/height (m2). Participants were categorised into the following BMI categories based on 2.5-unit increments as follows: <20, 20-22.49, 22.5-24.99, 25-27.49, 27.5-29.99, 30-32.49 32.5-34.99, ≥35 kg/m2.

Systolic blood pressure

Systolic blood pressure (SBP) measurements were taken by trained researched staff using an electronic blood pressure monitor or using a manual sphygmomanometer with an inflatable cuff and a stethoscope if automated measurements failed. All measurements were taken from participants while seated and from the left brachial artery, where possible. Deciles of systolic blood pressure were calculated using the mean of two SBP measurements in millimetres of mercury (mmHg).

Fruit and vegetable intake

Fruit and vegetable intake was calculated as a marker of healthy diet in quintiles of grams per day (g/d) from the sum of raw salad, green leafy vegetables, root vegetables, tomatoes, allium vegetables, other vegetables, peas and corn, citrus fruit, berries, apples and pears, and other fruits using responses to ≥ two 24-hour dietary assessments.

Saturated fatty acid intake

Quintiles of saturated fatty acid intake as a percentage of total energy intake were calculated using responses to ≥ two 24-hour dietary assessments.

Total energy intake

Sex-specific quintiles of total energy intake in kilojoules (kJ) per day were calculated using responses to ≥ two 24-hour dietary assessments.

Serum biomarkers measured by clinical chemistry

Serum low-density lipoprotein cholesterol (LDL-C), high-density lipoprotein cholesterol (HDL-C), triglycerides, and glycated haemoglobin (HbA1c) were measured from non-fasting blood samples collected from participants at baseline. Full details of the UK Biobank sample collection, handling and storage procedures are described elsewhere [3, 4]. For LDL-C, triglycerides and HbA1c, participants were categorised into deciles of serum concentrations from lowest (decile 1) to highest (decile 10). For HDL-C, participants were categorised into sex-specific quintiles of serum concentrations from lowest (quintile 1) to highest (quintile 5). Participants were categorised as unknown if they did not have the serum biomarker measurement at baseline because venepuncture could not be performed, or the measurement was excluded during assay quality control procedures.

Statin use

Statin use was determined by participant responses to the baseline medication interview. Firstly, a list of statin medications (HMG-CoA reductase inhibitors) prescribed in the UK was obtained from the National Institute for Clinical Excellence [5]. The UK biobank variable for treatments and medications (data field: 20003) was hand-searched for generic and branded statin medication names using the National Health Services ‘Medicines A to Z’ handbook, including; atorvastatin, cerivastatin, fluvastatin, lovastatin, pravastatin, rosuvastatin and simvastatin. Participants were categorised into three groups based on their responses to the medication interview at baseline as follows: non-statin users, statin users, or unknown.

Fasting status

Participant responses to the question ‘Time since last meal or drink (except plain water)’ was used to categorise fasting status for baseline blood collection as follows: < two hours, two hours, three hours, four hours, ≥ five hours, or unknown.

*Measurement and analyses of triglycerides in lipoprotein classes*

Non-fasting baseline plasma samples (aliquot 3) from ~118,000 UK Biobank participants were randomly selected for metabolic biomarker profiling using high-throughput nuclear magnetic resonance (NMR) spectroscopy [6]. Plasma samples were thawed (at +4°C), mixed, and centrifuged (3 min, 3400´g), aliquoted (at least 85 µL plasma), and transported (at +6°C), to Nightingale Health Laboratories (Helsinki, Finland) for NMR spectroscopy between June 2019 and April 2020. This method has allowed for simultaneous quantification of 249 metabolic biomarkers (168 absolute measures and 81 derived ratio measures), common lipids and lipids within lipoprotein subclasses.

Nightingale Health’s measurements are congruent with clinical chemistry measurements in cohort studies for some biomarkers, including total triglycerides [6, 7]. The NMR spectroscopy measurements in UK Biobank were performed on plasma samples from aliquot 3, whereas clinical chemistry measurements were performed on plasma samples from aliquot 1. Plasma samples from aliquot 3 are expected to be 5-10% diluted, as described in detail elsewhere [8], which may have some impacted the biomarker concentrations. The correlations between biomarkers measured by NMR spectroscopy and clinical chemistry in UK Biobank are expected to be lower due to dilution issues. The UK Biobank has performed quality control procedures for NMR measures, which are detailed elsewhere [8], and sample quality was reported in the UK Biobank showcase, including flags for samples that were below the limit of quantification (LOQ) [9].

We examined the cross-sectional associations of carbohydrate intakes with concentrations of plasma triglycerides in lipoprotein subclasses measured using NMR spectroscopy, because carbohydrate intakes (including free sugars) were most strongly associated with serum triglycerides measured using clinical chemistry in our previous analyses of macronutrient intakes and serum lipids in the UK Biobank

[10]. The Nightingale biomarker platform has allowed for quantification of concentrations of plasma triglycerides in five major lipoprotein classes based on particle density (chylomicrons, very low-density lipoprotein [VLDL], low-density lipoprotein [LDL], intermediate-density lipoprotein [IDL], and high-density lipoprotein [HDL]) further separated into subclasses based on particle size (extremely large, very large, large, medium, small, very small). In total, concentrations of 18 plasma triglyceride measurements (including total triglycerides and triglycerides in lipoprotein subclasses) were measured in the subsample of UK Biobank participants who had metabolic biomarker profiling (n=26,095, ~23.6% of our main sample). Triglyceride measurements with ≥20% of values below the LOQ (including missing values) were excluded (n=1; triglycerides from very large HDL) (see Table S2). We also set the values of triglyceride measurements that were flagged as below the LOQ to 50% of the minimum measurable value for that triglyceride measurement [11]. In total, 17 plasma triglyceride measurements (total triglycerides and triglycerides within 16 lipoprotein subclasses) were included in our analyses, which are described in the main methods section. Sensitivity analyses were performed by restricting to: (i) participants who were fasting for ≥ four hours at the time of blood collection at baseline (n=11,076), and (ii) participants with all plasma triglyceride measurement above the LOQ (N=21,865).

**References**

1. Townsend P, Phillimore P, Beattie A. Health and deprivation: inequality and the North. London, UK: Croom Helm Ltd; 1988.

2. Bradbury KE, Guo W, Cairns BJ, Armstrong ME, Key TJ. Association between physical activity and body fat percentage, with adjustment for BMI: a large cross-sectional analysis of UK Biobank. BMJ Open. 2017; 7:e011843 <https://doi.org/10.1136/bmjopen-2016-011843>.

3. Elliott P, Peakman TC. The UK Biobank sample handling and storage protocol for the collection, processing and archiving of human blood and urine. Int J Epidemiol 2008; 37:234-44 <https://doi.org/10.1093/ije/dym276>.

4. Fry S, Almond R, Moffat S, Gordon M, Singh P. UK Biobank biomarker project: companion document to accompany serum biomarker data. UK Biobank, Oxford. 2019. <https://biobank.ndph.ox.ac.uk/showcase/showcase/docs/serum_biochemistry.pdf>. Accessed 11 Jul 2022.

5. National Institute for Health and Care Excellence. Lipid-modifying drugs. National Institute for Health and Care Excellence, London. <https://www.nice.org.uk/advice/ktt3/resources/lipidmodifying-drugs-pdf-1632168482245>. Accessed Sep 10 2021.

6. UK Biobank. Nightingale Health metabolic biomarkers: phase 1 release. UK Biobank, Oxford. 2021. <https://biobank.ndph.ox.ac.uk/showcase/ukb/docs/nmrm_companion_doc.pdf>. Accessed 4 May 2022.

7. Tikkanen E, Jägerroos V, Holmes MV, Sattar N, Ala-Korpela M, Jousilahti P, et al. Metabolic biomarker discovery for risk of peripheral artery disease compared with coronary artery disease: lipoprotein and metabolite profiling of 31 657 individuals from 5 prospective cohorts. J Am Heart Assoc. 2021; 10:e021995 <https://doi.org/10.1161/jaha.121.021995>.

8. UK Biobank. Biomarker assay quality procedures: approaches used to minimise systematic and random errors (and the wider epidemiological implications) UK Biobank, Oxford. 2019. <https://biobank.ndph.ox.ac.uk/showcase/ukb/docs/nmrm_app4.pdf>. Accessed 10 Apr 2022.

9. UK Biobank. Flag explanations. UK Biobank, Oxford. 2021. <https://biobank.ndph.ox.ac.uk/showcase/ukb/docs/nmrm_app4.pdf>. Accessed 4 Jul 2022.

10. Kelly RK, Watling CZ, Tong TYN, Piernas C, Carter JL, Papier K, et al. Associations between macronutrients from different dietary sources and serum lipids in 24 639 UK Biobank study participants. Arterioscler Thromb Vasc Biol. 2021; 41:2190-200 <https://doi.org/10.1161/atvbaha.120.315628>.

11. Schmidt JA, Fensom GK, Rinaldi S, Scalbert A, Gunter MJ, Holmes MV, et al. NMR metabolite profiles in male meat-eaters, fish-eaters, vegetarians and vegans, and comparison with MS metabolite profiles. Metabolites. 2021; 11:121 <https://doi.org/10.3390/metabo11020121>.

12. Scientific Advisory Committee on Nutrition. Carbohydrates and health. Public Health England, London. 2015. <https://www.gov.uk/government/publications/sacn-carbohydrates-and-health-report>. Accessed 11 Jun 2022.

13. Perez-Cornago A, Pollard Z, Young H, van Uden M, Andrews C, Piernas C, et al. Description of the updated nutrition calculation of the Oxford WebQ questionnaire and comparison with the previous version among 207,144 participants in UK Biobank. Eur J Nutr. 2021; 60:4019-30 <https://doi.org/10.1007/s00394-021-02558-4>.

14. Piernas C, Perez-Cornago A, Gao M, Young H, Pollard Z, Mulligan A, et al. Describing a new food group classification system for UK biobank: analysis of food groups and sources of macro- and micronutrients in 208,200 participants. Eur J Nutr. 2021; 60:2879-90 <https://doi.org/10.1007/s00394-021-02535-x>.

## Additional File 1: Tables

#### **Table S1.** Description of types and sources of dietary carbohydrates [12-14].

| **Carbohydrate intakes** | **Description** |
| --- | --- |
| Total carbohydrates | Carbohydrates from all foods and beverages. |
| *Types of dietary carbohydrates* | |
| Total sugars | Sugars from all foods and beverages. |
| Free sugars | All monosaccharides and disaccharides added to foods by the manufacturer, cook or consumer, plus sugars naturally present in honey, syrups and unsweetened fruit juices. Lactose when naturally present in milk and milk products is excluded. |
| Non-free sugars | Sugars naturally occurring in milk and dairy products, fresh and most types of processed fruit and vegetables and potatoes except for juices and purees, and cereal grains. Calculated as total sugars minus free sugars. |
| Fibre | Non-starch polysaccharide, comprising cellulose and non-cellulose polysaccharides (e.g. pectins, glucans, arabinogalactans, arabinoxylans, gums and mucilages) by Englyst from all foods and beverages. |
| *Sources of dietary carbohydrates^a^* | |
| Refined grain starch | Starch content of food containing refined grain flour, white bread, other bread (e.g. naan, garlic bread), other cereal (sugar) (e.g. Kellogg’s Cornflakes cereal), white pasta & rice, pizza, samosa, pakora, grain dishes (added fat), savoury snacks, biscuits, other desserts & cakes & pastries, and savoury crackers. |
| Wholegrain starch | Starch content foods containing wholegrain flour, including wholemeal bread, bran cereal (e.g. bran flakes, All Bran), biscuit cereal (e.g. Weetabix, Shredded Wheat, Shreddies), oat cereal (non-sugar), oat cereal (sugar), muesli, wholemeal pasta, brown rice & other wholegrains. |
| ^a^Sources of dietary carbohydrates were calculated from 93 food groups. | |

#### **Table S2.** Outcome definitions for incident CVD and exclusion criteria for prevalent CVD and diabetes.

| **CVD outcome** | **Outcome definition using ICD-10** | **Outcome definition using OPCS-4 codes** | **Exclusion criteria using ICD-10** | **Exclusion criteria using OPCS-4^8^** | **Exclusion criteria using ICD-9** | **Exclusion criteria using touchscreen** | **Exclusion criteria using UKB interviews for outcomes^a^** | **Exclusion criteria using UKB interviews for** **procedures^b^** | **Exclusion criteria using UKB interview for medications reported at recruitment^c^** |
| --- | --- | --- | --- | --- | --- | --- | --- | --- | --- |
| Total CVD | I21, I22, I23, I24, I25, I60, I61, I63, I64 | K40, K41, K42, K43, K44, K45, K46, K49, K50, K75 | I48, I20, I21, I22, I23, I24, I25, G45, I60, I61, I62, I63, I64, I65, I66, I67, I68, I69 | K49, K50, K75, K40, K41, K42, K43, K44, K45, K46 | 427.31, 427.32, 410, 411, 412, 413, 414, 430, 431, 432, 433, 434, 435, 436, 437, 438 | UKB variable 'Has a doctor ever told you that you have the following conditions 6150?'  1) Heart attack 2) angina  3) stroke  UKB variable 'Has a doctor ever told you that you have diabetes 2443?’ 1) Yes  UKB variable | 1471 atrial fibrillation  1483 atrial flutter  1074 angina  1075 heart attack/myocardial infarction  1081 stroke  1082 transient ischaemic attack1083 subdural haemorrhage/haematoma  1086 subarachnoid haemorrhage | 1070 coronary angioplasty (PTCA) +/- stent  1095 coronary artery bypass graft (CABG) | 1140884600 1141189090 1140874686 1140874646 1140874650 1140874652 1140874658 1140874660 1140874664 1140874666 1140874674 1140874678 1140874680 1140874686 1140874690 1140874706 1140874712 1140874716 1140874718 1140874724 1140874726 1140874728 1140874732 1140874736 1140874740 1140874744 1140874746 1141152590 1141156984 1141157284 1140857494 1140857496 1140857500 1140857502 1140857506 1140857508 1140857518 1140857586 1140868902 1140868908 1141153254 1141153262 1141171646 1141171652 1141177600 1141177606 1141189090 1141189094 1141168660 1141168668 1141173882 1141173888 1141173786 1140883066 |
| IHD | I21, I22, I23, I24, I25 | K40, K41, K42, K43, K44, K45, K46, K49, K50, K75 |  |  |  |  |  |  |  |
| AMI | I21 | - |  |  |  |  |  |  |  |
| Total stroke | I60, I61, I63, I64 | - |  |  |  |  |  |  |  |
| Ischaemic stroke | I63 | - |  |  |  |  |  |  |  |
| Haemorrhagic stroke | I60, I61 | - |  |  |  |  |  |  |  |
| ^a^ Based on ICD-10 definition and UKB variable 20002  ^b^ Based on OPSC-4 definition and UKB variable 20004  ^c^ Based on BNF formulary of diabetic medications and UKB variable 20003  *Abbreviations: AMI* acute myocardial infarction, *BNF* British National Formulary, *CABG* coronary artery bypass graft, *CVD* cardiovascular disease, *ICD* international classification of diseases, *IHD* ischaemic heart disease, *OPCS* Classification of Interventions and Procedures, *PTCA* percutaneous transluminal coronary angioplasty, *UKB* UK Biobank. | | | | | | | | | |

#### **Table S3.** Triglyceride concentrations measured by NMR spectroscopy flagged as being below the limit of quantification in 26,095 UK Biobank participants.

| **Triglycerides** | | **Number of participants (%) with measurements below the LOQ** |
| --- | --- | --- |
| Total triglycerides | | - |
| Triglycerides in VLDL | | - |
|  | Triglycerides in extremely large VLDL | - |
|  | Triglycerides in very large VLDL | 987 (3.8%) |
|  | Triglycerides in large VLDL | 8 (<0.001%) |
|  | Triglycerides in medium VLDL | 1 (<0.001%) |
|  | Triglycerides in small VLDL | - |
|  | Triglycerides in very small VLDL | - |
| Triglycerides in IDL | | - |
| Triglycerides in LDL | | - |
|  | Triglycerides in large LDL | - |
|  | Triglycerides in medium LDL | 1 (<0.001%) |
|  | Triglycerides in small LDL | 3,877 (14.9%) |
| Triglycerides in HDL | | - |
|  | Triglycerides in very large HDL^a^ | 23,229 (89.0%) |
|  | Triglycerides in large HDL | 260 (0.01%) |
|  | Triglycerides in medium HDL | 14 (<0.001%) |
|  | Triglycerides in small HDL | 14 (<0.001%) |
| ^a^Triglyceride measurements with ≥20% of values below the LOQ.  *Abbreviations: HDL* high-density lipoprotein, *IDL* intermediate-density lipoprotein, *LDL* low-density lipoprotein, *LOQ* limit of quantification, *NMR* nuclear magnetic resonance, *VLDL* very low-density lipoprotein. | | |

#### **Table S4.** Baseline characteristics across quartiles of total carbohydrate intake in 110,497 UK Biobank participants.

| **Characteristics** | | **Total carbohydrate intake** | | | | ***P*-value^a^** |
| --- | --- | --- | --- | --- | --- | --- |
|  |  | **Q1** | **Q2** | **Q3** | **Q4** |  |
| N | | 27,625 | 27,624 | 27,624 | 27,624 |  |
| Total carbohydrate intake (% energy intake)^b^ | | 39.9 (4.6) | 47.4 (1.4) | 51.9 (1.3) | 58.3 (3.5) | <0.001 |
| Sociodemographic characteristics | |  |  |  |  |  |
|  | Age at recruitment | 55.7 (7.7) | 55.8 (7.8) | 55.8 (7.9) | 55.6 (8.0) | <0.001 |
|  | Female sex, n (%) | 15,111 (54.7%) | 15,759 (57.0%) | 16,404 (59.4%) | 16,700 (60.5%) | <0.001 |
|  | White ethnicity, n (%) | 26,929 (97.8%) | 26,851 (97.6%) | 26,806 (97.3%) | 26,313 (95.5%) | <0.001 |
|  | Most affluent, n (%)^c^ | 5,630 (21.2%) | 5,957 (23.0%) | 5,970 (23.1%) | 5,963 (21.4%) | <0.001 |
|  | College or university degree, n (%) | 20,760 (79.2%) | 20,547 (78.9%) | 20,322 (78.5%) | 20,006 (78.3%) | <0.001 |
| Lifestyle | |  |  |  |  |  |
|  | Alcohol (g/d)^d^ | 24.6 (20.7) | 16.4 (14.7) | 12.6 (11.9) | 9.9 (11.9) | <0.001 |
|  | Current smoker, n (%) | 2,755 (10.0%) | 1,901 (6.9%) | 1,495 (5.4%) | 1,344 (4.9%) | <0.001 |
|  | Physical activity (excess MET h/wk) | 37.0 (38.2) | 37.7 (38.9) | 38.7 (39.7) | 40.7 (41.7) | <0.001 |
|  | Energy intake (kJ/d) | 8782 (2033) | 8770 (1913) | 8588 (1850) | 8187 (1851) | <0.001 |
|  | SFA intake (% energy intake) | 12.8 (3.1) | 12.3 (2.8) | 11.6 (2.5) | 10.0 (2.4) | <0.001 |
|  | Fruit and vegetable intake (g/d) | 327.6 (197.6) | 364.3 (204.8) | 393.8 (213.7) | 453.8 (256.0) | <0.001 |
| Medical history | |  |  |  |  |  |
|  | Statin use, n (%) | 2,573 (9.3%) | 2,362 (8.6%) | 2,207 (8.0%) | 2,314 (8.4%) | <0.001 |
|  | Postmenopausal, n (%)^e^ | 10,234 (71.8%) | 10,848 (72.8%) | 11,313 (72.9%) | 11,316 (72.0%) | <0.001 |
| Biological measurements | |  |  |  |  |  |
|  | BMI (kg/m^2^) | 26.9 (4.4) | 26.4 (4.3) | 26.3 (4.4) | 26.2 (4.4) | <0.001 |
|  | Waist circumference (cm) | 89.1 (13.0) | 87.7 (12.6) | 86.8 (12.5) | 86.3 (12.6) | <0.001 |
|  | SBP (mmol/L) | 137.2 (18.3) | 136.3 (18.2) | 136.0 (18.2) | 135.9 (18.5) | <0.001 |
|  | LDL-C (mmol/L)^f^ | 3.66 (0.83) | 3.64 (0.82) | 3.62 (0.81) | 3.59 (0.82) | <0.001 |
|  | HDL-C (mmol/L)^f^ | 1.58 (0.41) | 1.52 (0.38) | 1.49 (0.37) | 1.45 (0.36) | <0.001 |
|  | Triglycerides (mmol/L)^f^ | 1.62 (0.96) | 1.63 (0.94) | 1.64 (0.94) | 1.67 (0.96) | <0.001 |
|  | ApoB (mmol/L)^f^ | 1.05 (0.23) | 1.04 (0.23) | 1.04 (0.23) | 1.03 (0.23) | <0.001 |
|  | HbA1c (mmol/mol) | 34.68 (4.48) | 34.76 (4.26) | 34.75 (4.06) | 34.71 (4.17) | 0.12 |
| Numbers are means (SD) unless otherwise specified as numbers (%), with % representing the column percentage estimated excluding participants with missing responses.  ^a^ *P*-values obtained from χ^2^ tests comparing the distribution between quartiles of total carbohydrate intake for categorical variables, and analysis of variance (ANOVA) to compare the means between quartiles of total carbohydrate intake for continuous variables. *P*-values ≥0.1 are displayed to two decimal places and *P*-values <0.1 are displayed to three decimal places.  ^b^ Ranges of total carbohydrate intake within each quartile are shown in Table S7 (Additional File 1).  ^c^ Participants categorised in the lowest quintile of the Townsend deprivation index (i.e. least deprived).  ^d^ Excluding never drinkers.  ^e^ In women only.  ^f^ Measured from serum using standard clinical chemistry assays.  *Abbreviations*: Apo apolipoprotein, BMI body mass index, h/wk hours per week, HbA1c glycated haemoglobin, HDL-C high-density lipoprotein cholesterol, kg/m2 kilogram per square metre, kJ/d kilojoules per day, LDL-C low-density lipoprotein cholesterol, MET metabolic equivalent task, mmHg millimetres of mercury, mmol/L millimoles per litre, mmol/mol millimoles per mol, Q quartile, SBP systolic blood pressure, SD standard deviation, SFA saturated fatty acid. | | | | | | |

#### **Table S5.** Baseline characteristics across quartiles of total carbohydrate intake in 110,497 UK Biobank participants.

| **Characteristics** | | **Free sugar intake** | | | | ***P*-value^a^** |
| --- | --- | --- | --- | --- | --- | --- |
|  |  | **Q1** | **Q2** | **Q3** | **Q4** |  |
| N | | 27,625 | 27,624 | 27,624 | 27,624 |  |
| Free sugar (% energy intake)^b^ | | 11.3 (2.1) | 15.7 (1.0) | 19.1 (1.1) | 25.3 (4.1) | <0.001 |
| Sociodemographic characteristics | |  |  |  |  |  |
|  | Age at recruitment | 54.6 (7.8) | 55.6 (7.8) | 56.2 (7.7) | 56.5 (7.8) | <0.001 |
|  | Female sex, n (%) | 16,552 (59.9%) | 16,672 (60.4%) | 16,213 (58.7%) | 14,537 (52.6%) | <0.001 |
|  | White ethnicity, n (%) | 26,423 (96.0%) | 26,811 (97.4%) | 26,859 (97.5%) | 26,806 (97.4%) | <0.001 |
|  | Most affluent, n (%)^c^ | 5,569 (21.0%) | 5,929 (22.4%) | 6,052 (23.3%) | 5,970 (21.9%) | <0.001 |
|  | College or university degree, n (%) | 18,971 (74.1%) | 20,222 (78.0%) | 20,987 (80.4%) | 21,455 (82.3%) | <0.001 |
| Lifestyle | |  |  |  |  |  |
|  | Alcohol (g/d)^d^ | 19.2 (19.6) | 16.1 (15.3) | 15.1 (14.6) | 14.6 (15.2) | <0.001 |
|  | Current smoker, n (%) | 2,941 (10.7%) | 1,765 (6.4%) | 1,497 (5.4%) | 1,292 (4.7%) | <0.001 |
|  | Physical activity (excess MET h/wk) | 34.5 (38.4) | 36.1 (37.4) | 38.7 (38.7) | 44.8 (43.1) | 0.001 |
|  | Energy intake (kJ/d) | 7387 (1612) | 8260 (1619) | 8850 (1693) | 9831 (1905) | <0.001 |
|  | SFA intake (% energy intake) | 12.2 (3.1) | 11.9 (2.9) | 11.6 (2.8) | 10.9 (2.7) | <0.001 |
|  | Fruit and vegetable intake (g/d) | 221.3 (126.8) | 327.3 (145.4) | 411.9 (170.5) | 579.0 (256.8) | <0.001 |
| Medical history | |  |  |  |  |  |
|  | Statin use, n (%) | 2,432 (8.8%) | 2,420 (8.8%) | 2,308 (8.4%) | 2,296 (8.3%) | 0.029 |
|  | Postmenopausal, n (%)^e^ | 10,503 (68.0%) | 11,274 (71.9%) | 11,426 (74.3%) | 10,508 (75.7%) | <0.001 |
| Biological measurements | |  |  |  |  |  |
|  | BMI (kg/m^2^) | 26.9 (4.5) | 26.6 (4.4) | 26.2 (4.3) | 25.9 (4.3) | <0.001 |
|  | Waist circumference (cm) | 88.4 (13.0) | 87.6 (12.8) | 87.0 (12.6) | 87.0 (12.6) | <0.001 |
|  | SBP (mmol/L) | 136.1 (18.3) | 136.1 (18.3) | 136.5 (18.3) | 136.6 (18.3) | <0.001 |
|  | LDL-C (mmol/L)^f^ | 3.67 (0.83) | 3.65 (0.82) | 3.62 (0.81) | 3.57 (0.80) | 0.16 |
|  | HDL-C (mmol/L)^f^ | 1.52 (0.40) | 1.52 (0.39) | 1.52 (0.38) | 1.49 (0.37) | <0.001 |
|  | Triglycerides (mmol/L)^f^ | 1.66 (0.98) | 1.64 (0.94) | 1.63 (0.94) | 1.63 (0.94) | <0.001 |
|  | ApoB (mmol/L)^f^ | 1.06 (0.23) | 1.05 (0.23) | 1.04 (0.23) | 1.03 (0.22) | <0.001 |
|  | HbA1c (mmol/mol) | 34.6 (4.1) | 34.7 (4.4) | 34.7 (4.2) | 34.8 (4.2) | 0.023 |
| Numbers are means (SD) unless otherwise specified as numbers (%), with % representing the column percentage estimated excluding participants with missing responses.  ^a^ *P*-values obtained from χ^2^ tests comparing the distribution between quartiles of free sugar intake for categorical variables, and analysis of variance (ANOVA) to compare the means between quartiles of free sugar intake for continuous variables. *P*-values ≥0.1 are displayed to two decimal places and *P*-values <0.1 are displayed to three decimal places.  ^b^ Ranges of free sugar intake within each quartile are shown in Table S7 (Additional File 1).  ^c^ Participants categorised in the lowest quintile of the Townsend deprivation index (i.e. least deprived).  ^d^ Excluding never drinkers.  ^e^ In women only.  ^f^ Measured from serum using standard clinical chemistry assays.  *Abbreviations*: Apo apolipoprotein, BMI body mass index, h/wk hours per week, HbA1c glycated haemoglobin, HDL-C high-density lipoprotein cholesterol, kg/m2 kilogram per square metre, kJ/d kilojoules per day, LDL-C low-density lipoprotein cholesterol, MET metabolic equivalent task, mmHg millimetres of mercury, mmol/L millimoles per litre, mmol/mol millimoles per mol, Q quartile, SBP systolic blood pressure, SD standard deviation, SFA saturated fatty acid. | | | | | | |

#### **Table S6.** Baseline characteristics across quartiles of fibre intake in 110,497 UK Biobank participants.

| **Characteristics** | | **Fibre intake** | | | | ***P*-value^a^** |
| --- | --- | --- | --- | --- | --- | --- |
|  |  | **Q1** | **Q2** | **Q3** | **Q4** |  |
| N | | 27,625 | 27,624 | 27,624 | 27,624 |  |
| Fibre intake (g/d)^b^ | | 5.9 (1.7) | 9.5 (0.8) | 12.5 (0.9) | 17.9 (3.6) | <0.001 |
| Sociodemographic characteristics | |  |  |  |  |  |
|  | Age at recruitment | 55.9 (7.6) | 55.9 (7.7) | 55.9 (7.8) | 55.2 (8.1) | <0.001 |
|  | Female sex, n (%) | 18,388 (66.6%) | 16,645 (60.3%) | 15,430 (55.9%) | 13,511 (48.9%) | <0.001 |
|  | White ethnicity, n (%) | 26,732 (97.1%) | 26,877 (97.6%) | 26,842 (97.4%) | 26,448 (96.1%) | <0.001 |
|  | Most affluent, n (%)^c^ | 5,679 (21.4%) | 6,051 (22.9%) | 6,025 (22.8%) | 5,765 (21.7%) | <0.001 |
|  | College or university degree, n (%) | 20,550 (79.1%) | 20,642 (79.3%) | 20,703 (79.6%) | 19,740 (76.8%) | <0.001 |
| Lifestyle | |  |  |  |  |  |
|  | Alcohol (g/d)^d^ | 16.0 (15.9) | 16.0 (15.1) | 16.1 (15.6) | 17.0 (19.0) | <0.001 |
|  | Current smoker, n (%) | 1,801 (6.5%) | 1,513 (5.5%) | 1,701 (6.2%) | 2,480 (9.0%) | <0.001 |
|  | Physical activity (excess MET h/wk) | 38.0 (38.5) | 38.5 (39.0) | 38.3 (38.9) | 39.3 (42.1) | <0.001 |
|  | Energy intake (kJ/d) | 8088 (1850) | 8575 (1862) | 8770 (1894) | 8895 (2005) | <0.001 |
|  | SFA intake (% energy intake) | 11.4 (3.1) | 11.7 (2.9) | 11.8 (2.8) | 11.6 (2.9) | <0.001 |
|  | Fruit and vegetable intake (g/d) | 450.5 (253.7) | 400.6 (217.3) | 369.7 (203.2) | 318.8 (196.7) | <0.001 |
| Medical history | |  |  |  |  |  |
|  | Statin use, n (%) | 2,264 (8.2%) | 2,329 (8.4%) | 2,387 (8.6%) | 2,476 (9.0%) | 0.21 |
|  | Postmenopausal, n (%)^e^ | 12,859 (73.6%) | 11,599 (73.6%) | 10,555 (72.3%) | 8,698 (69.1%) | <0.001 |
| Biological measurements | |  |  |  |  |  |
|  | BMI (kg/m^2^) | 26.6 (4.6) | 26.3 (4.4) | 26.3 (4.3) | 26.5 (4.3) | <0.001 |
|  | Waist circumference (cm) | 86.8 (13.0) | 87.0 (12.6) | 87.4 (12.6) | 88.8 (12.6) | <0.001 |
|  | SBP (mmol/L) | 135.9 (18.4) | 136.3 (18.3) | 136.5 (18.2) | 136.7 (18.3) | <0.001 |
|  | LDL-C (mmol/L)^f^ | 3.62 (0.82) | 3.62 (0.82) | 3.63 (0.81) | 3.63 (0.82) | <0.001 |
|  | HDL-C (mmol/L)^f^ | 1.56 (0.39) | 1.54 (0.39) | 1.51 (0.38) | 1.44 (0.37) | <0.001 |
|  | Triglycerides (mmol/L)^f^ | 1.55 (0.90) | 1.60 (0.92) | 1.65 (0.95) | 1.76 (1.02) | <0.001 |
|  | ApoB (mmol/L)^f^ | 1.04 (0.23) | 1.04 (0.23) | 1.04 (0.23) | 1.05 (0.23) | <0.001 |
|  | HbA1c (mmol/mol) | 34.8 (4.7) | 34.7 (4.0) | 34.7 (4.2) | 34.7 (4.1) | <0.001 |
| Numbers are means (SD) unless otherwise specified as numbers (%), with % representing the column percentage estimated excluding participants with missing responses.  ^a^ *P*-values obtained from χ^2^ tests comparing the distribution between quartiles of fibre intake for categorical variables, and analysis of variance (ANOVA) to compare the means between quartiles of fibre intake for continuous variables. *P*-values ≥0.1 are displayed to two decimal places and *P*-values <0.1 are displayed to three decimal places.  ^b^ Ranges of fibre intake within each quartile are shown in Table S7 (Additional File 1).  ^c^ Participants categorised in the lowest quintile of the Townsend deprivation index (i.e. least deprived).  ^d^ Excluding never drinkers.  ^e^ In women only.  ^f^ Measured from serum using standard clinical chemistry assays.  *Abbreviations*: Apo apolipoprotein, BMI body mass index, h/wk hours per week, HbA1c glycated haemoglobin, HDL-C high-density lipoprotein cholesterol, kg/m2 kilogram per square metre, kJ/d kilojoules per day, LDL-C low-density lipoprotein cholesterol, MET metabolic equivalent task, mmHg millimetres of mercury, mmol/L millimoles per litre, mmol/mol millimoles per mol, Q quartile, SBP systolic blood pressure, SD standard deviation, SFA saturated fatty acid. | | | | | | |

#### **Table S7.** Carbohydrate intakes in grams per day and percentage of energy intake by quartiles of carbohydrate intakes.

| **Carbohydrate intakes** | | **Mean (SD)** | | | |
| --- | --- | --- | --- | --- | --- |
|  |  | **Q1 (Lowest)** | **Q2** | **Q3** | **Q4 (Highest)** |
| Total carbohydrates | |  |  |  |  |
|  | N | 27,625 | 27,624 | 27,624 | 27,624 |
|  | Mean (SD), g/d | 209.82 (55.19) | 247.90 (54.65) | 265.61 (57.66) | 283.68 (63.93) |
|  | Mean (SD), % energy intake | 39.89 (4.57) | 47.41 (1.38) | 51.90 (1.30) | 58.27 (3.48) |
|  | Range, % energy intake | 0.49 - 44.84 | 44.85 - 49.69 | 49.70 - 54.22 | 54.23 - 87.16 |
| Refined grain starch | |  |  |  |  |
|  | N | 27,625 | 27,624 | 27,624 | 27,624 |
|  | Mean (SD), g/d | 22.34 (11.71) | 47.70 (12.66) | 69.26 (17.24) | 103.60 (30.94) |
|  | Mean (SD), % energy intake | 4.34 (1.94) | 9.08 (1.13) | 13.10 (1.26) | 19.90 (3.92) |
|  | Range, % energy intake | 0.00 - 7.07 | 7.08 - 11.01 | 11.02 - 15.41 | 15.42 - 50.83 |
| Wholegrain starch | |  |  |  |  |
|  | N | 27,625 | 27,624 | 27,624 | 27,624 |
|  | Mean (SD), g/d | 3.54 (4.56) | 18.05 (5.67) | 30.41 (7.90) | 52.27 (18.16) |
|  | Mean (SD), % energy intake | 0.64 (0.79) | 3.48 (0.73) | 6.05 (0.81) | 10.87 (3.07) |
|  | Range, % energy intake | 0.00 - 2.16 | 2.17 - 4.71 | 4.72 - 7.56 | 7.57 - 48.89 |
| Total sugars | |  |  |  |  |
|  | N | 27,625 | 27,624 | 27,624 | 27,624 |
|  | Mean (SD), g/d | 87.21 (24.77) | 114.81 (25.94) | 133.19 (29.96) | 160.41 (40.28) |
|  | Mean (SD), % energy intake | 16.65 (2.69) | 22.09 (1.15) | 26.14 (1.25) | 32.94 (4.11) |
|  | Range, % energy intake | 0.44 - 20.01 | 20.02 - 24.05 | 24.06 - 28.44 | 28.45 - 73.29 |
| Free sugars | |  |  |  |  |
|  | N | 27,625 | 27,624 | 27,624 | 27,624 |
|  | Mean (SD), g/d | 29.15 (11.28) | 49.10 (11.82) | 65.45 (15.33) | 94.99 (28.83) |
|  | Mean (SD), % energy intake | 5.88 (1.66) | 9.51 (0.81) | 12.45 (0.93) | 17.92 (3.64) |
|  | Range, % energy intake | 0.00 - 8.06 | 8.07 - 10.90 | 10.91 - 14.18 | 14.19 - 59.16 |
| Non-free sugars | |  |  |  |  |
|  | N | 27,625 | 27,624 | 27,624 | 27,624 |
|  | Mean (SD), g/d | 36.78 (11.87) | 55.65 (12.88) | 70.07 (15.75) | 94.43 (25.99) |
|  | Mean (SD), % energy intake | 6.80 (1.61) | 10.67 (0.92) | 14.07 (1.10) | 20.51 (4.09) |
|  | Range, % energy intake | 0.40 - 9.04 | 9.05 - 12.26 | 12.27 - 16.12 | 16.13 - 60.42 |
| Fibre | |  |  |  |  |
|  | N | 27,625 | 27,624 | 27,624 | 27,624 |
|  | Mean (SD), g/d | 11.34 (2.07) | 11.34 (2.07) | 15.69 (0.95) | 19.11 (1.08) |
|  | Range, g/d | 0.25 - 13.98 | 0.25 - 13.98 | 13.99 - 17.31 | 17.32 - 21.10 |
| Numbers are means (SD).  *Abbreviations: g/d* grams per day, *Q* quartile, *SD* standard deviation. | | | | | |

#### **Table S8.** Types and sources of carbohydrates in grams per day and percentage of energy intake by quartiles of total carbohydrate intake.

| **Types and sources of carbohydrate intake** | | **Total carbohydrate intake** | | | |
| --- | --- | --- | --- | --- | --- |
|  |  | **Q1 (Lowest)** | **Q2** | **Q3** | **Q4 (Highest)** |
| N |  | 27,625 | 27,624 | 27,624 | 27,624 |
| Refined grain starch | |  |  |  |  |
|  | g/d | 52.43 (32.32) | 61.70 (34.72) | 64.24 (36.28) | 64.53 (37.98) |
|  | % of total energy intake | 9.70 (5.31) | 11.52 (5.76) | 12.24 (6.16) | 12.95 (6.86) |
| Wholegrain starch | |  |  |  |  |
|  | g/d | 18.78 (17.41) | 25.14 (19.36) | 28.61 (20.79) | 31.73 (22.81) |
|  | % of total energy intake | 3.66 (3.33) | 4.94 (3.70) | 5.74 (4.05) | 6.69 (4.66) |
| Total sugars | |  |  |  |  |
|  | g/d | 99.49 (32.51) | 118.95 (34.19) | 130.61 (36.85) | 146.58 (43.49) |
|  | % of total energy intake | 19.08 (4.59) | 22.87 (4.50) | 25.64 (4.77) | 30.24 (6.17) |
| Free sugars | |  |  |  |  |
|  | g/d | 50.22 (25.72) | 58.28 (27.50) | 62.69 (29.48) | 67.50 (34.77) |
|  | % of total energy intake | 9.35 (3.91) | 10.90 (4.13) | 11.98 (4.50) | 13.54 (5.76) |
| Non-free sugars | |  |  |  |  |
|  | g/d | 49.26 (20.91) | 60.68 (22.82) | 67.92 (24.89) | 79.08 (30.91) |
|  | % of total energy intake | 9.73 (4.04) | 11.97 (4.39) | 13.66 (4.78) | 16.70 (6.22) |
| Fibre | |  |  |  |  |
|  | g/d | 15.43 (5.02) | 17.55 (5.09) | 18.60 (5.36) | 19.89 (6.08) |
| Numbers are means (SD).  *Abbreviations: g/d* grams per day, *Q* quartile, *SD* standard deviation. | | | | | |

#### **Table S9.** Hazard ratios (95% confidence intervals) for the associations between types and sources of carbohydrates and acute myocardial infarction, ischaemic stroke, and haemorrhagic stroke risk in 110,497 UK Biobank participants.

| **Models** | | | **Hazard ratios (95% CI)** | | | | |  |
| --- | --- | --- | --- | --- | --- | --- | --- | --- |
|  |  |  | **Q1** | **Q2** | **Q3** | **Q4** | **For each 5%  of energy^a^** | ***P*-trend^b^** |
| *AMI* | | |  |  |  |  |  |  |
|  | Total carbohydrates | |  |  |  |  |  |  |
|  |  | Cases, n (%) | 336 | 345 | 353 | 348 | 1382 |  |
|  |  | HR (95% CI) | Ref | 1.05 (0.90 - 1.22) | 1.11 (0.95 - 1.30) | 1.11 (0.93 - 1.33) | 1.03 (0.99 - 1.08) | 0.13 |
|  | Refined grain starch | |  |  |  |  |  |  |
|  |  | Cases, n (%) | 345 | 359 | 338 | 340 | 1382 |  |
|  |  | HR (95% CI) | Ref | 1.06 (0.91 - 1.23) | 1.02 (0.87 - 1.19) | 1.05 (0.89 - 1.23) | 1.01 (0.96 - 1.05) | 0.74 |
|  | Wholegrain starch | |  |  |  |  |  |  |
|  |  | Cases, n (%) | 372 | 336 | 335 | 339 | 1382 |  |
|  |  | HR (95% CI) | Ref | 0.94 (0.81 - 1.09) | 0.95 (0.81 - 1.10) | 0.94 (0.81 - 1.10) | 0.95 (0.88 - 1.01) | 0.110 |
|  | Total sugars | |  |  |  |  |  |  |
|  |  | Cases, n (%) | 348 | 302 | 372 | 360 | 1382 |  |
|  |  | HR (95% CI) | Ref | 0.88 (0.75 - 1.02) | 1.09 (0.93 - 1.26) | 1.08 (0.92 - 1.27) | 1.03 (0.99 - 1.08) | 0.16 |
|  | Free sugars | |  |  |  |  |  |  |
|  |  | Cases, n (%) | 302 | 318 | 325 | 437 | 1382 |  |
|  |  | HR (95% CI) | Ref | 0.99 (0.85 - 1.17) | 0.95 (0.81 - 1.12) | 1.17 (1.00 - 1.36) | 1.10 (1.04 - 1.16) | 0.000* |
|  | Non-free sugars | |  |  |  |  |  |  |
|  |  | Cases, n (%) | 408 | 359 | 312 | 303 | 1382 |  |
|  |  | HR (95% CI) | Ref | 0.94 (0.81 - 1.09) | 0.87 (0.74 - 1.02) | 0.89 (0.75 - 1.06) | 0.93 (0.88 - 0.99) | 0.021 |
|  | Fibre | |  |  |  |  |  |  |
|  |  | Cases, n (%) | 366 | 332 | 320 | 364 | 1382 |  |
|  |  | HR (95% CI) | Ref | 0.89 (0.76 - 1.04) | 0.82 (0.69 - 0.97) | 0.83 (0.69 - 1.00) | 0.94 (0.89 - 1.00) | 0.057 |
| *Ischaemic stroke* | | |  |  |  |  |  |  |
|  | Total carbohydrates | |  |  |  |  |  |  |
|  |  | Cases, n (%) | 201 | 195 | 193 | 188 | 777 |  |
|  |  | HR (95% CI) | Ref | 1.05 (0.85 - 1.28) | 1.08 (0.87 - 1.34) | 1.08 (0.85 - 1.37) | 1.04 (0.98 - 1.10) | 0.18 |
|  | Refined grain starch | |  |  |  |  |  |  |
|  |  | Cases, n (%) | 207 | 212 | 176 | 182 | 777 |  |
|  |  | HR (95% CI) | Ref | 1.10 (0.90 - 1.33) | 0.99 (0.81 - 1.22) | 1.14 (0.93 - 1.41) | 1.01 (0.95 - 1.07) | 0.78 |
|  | Wholegrain starch | |  |  |  |  |  |  |
|  |  | Cases, n (%) | 194 | 199 | 207 | 177 | 777 |  |
|  |  | HR (95% CI) | Ref | 1.02 (0.84 - 1.25) | 1.05 (0.86 - 1.28) | 0.89 (0.72 - 1.11) | 0.99 (0.90 - 1.08) | 0.75 |
|  | Total sugars | |  |  |  |  |  |  |
|  |  | Cases, n (%) | 187 | 197 | 172 | 221 | 777 |  |
|  |  | HR (95% CI) | Ref | 1.04 (0.85 - 1.27) | 0.89 (0.72 - 1.11) | 1.15 (0.93 - 1.42) | 1.04 (0.98 - 1.10) | 0.23 |
|  | Free sugars | |  |  |  |  |  |  |
|  |  | Cases, n (%) | 166 | 197 | 178 | 236 | 777 |  |
|  |  | HR (95% CI) | Ref | 1.17 (0.95 - 1.44) | 1.03 (0.83 - 1.28) | 1.37 (1.11 - 1.68) | 1.12 (1.04 - 1.21) | 0.003* |
|  | Non-free sugars | |  |  |  |  |  |  |
|  |  | Cases, n (%) | 206 | 194 | 187 | 190 | 777 |  |
|  |  | HR (95% CI) | Ref | 0.91 (0.74 - 1.11) | 0.86 (0.70 - 1.07) | 0.86 (0.68 - 1.08) | 0.93 (0.87 - 1.01) | 0.085 |
|  | Fibre | |  |  |  |  |  |  |
|  |  | Cases, n (%) | 197 | 176 | 188 | 216 | 777 |  |
|  |  | HR (95% CI) | Ref | 0.84 (0.68 - 1.03) | 0.86 (0.69 - 1.07) | 0.92 (0.72 - 1.17) | 0.96 (0.88 - 1.04) | 0.30 |
| *Haemorrhagic stroke* | | |  |  |  |  |  |  |
|  | Total carbohydrates | |  |  |  |  |  |  |
|  |  | Cases, n (%) | 80 | 75 | 66 | 81 | 302 |  |
|  |  | HR (95% CI) | Ref | 0.89 (0.64 - 1.24) | 0.74 (0.52 - 1.05) | 0.78 (0.54 - 1.14) | 0.89 (0.81 - 0.98) | 0.013 |
|  | Refined grain starch | |  |  |  |  |  |  |
|  |  | Cases, n (%) | 83 | 89 | 71 | 59 | 302 |  |
|  |  | HR (95% CI) | Ref | 1.17 (0.86 - 1.58) | 1.00 (0.73 - 1.39) | 0.90 (0.63 - 1.27) | 0.95 (0.86 - 1.06) | 0.363 |
|  | Wholegrain starch | |  |  |  |  |  |  |
|  |  | Cases, n (%) | 83 | 73 | 71 | 75 | 302 |  |
|  |  | HR (95% CI) | Ref | 0.87 (0.63 - 1.19) | 0.80 (0.58 - 1.10) | 0.79 (0.57 - 1.10) | 0.93 (0.80 - 1.07) | 0.30 |
|  | Total sugars | |  |  |  |  |  |  |
|  |  | Cases, n (%) | 80 | 59 | 76 | 87 | 302 |  |
|  |  | HR (95% CI) | Ref | 0.69 (0.49 - 0.97) | 0.83 (0.60 - 1.16) | 0.84 (0.60 - 1.17) | 0.99 (0.90 - 1.09) | 0.85 |
|  | Free sugars | |  |  |  |  |  |  |
|  |  | Cases, n (%) | 79 | 71 | 73 | 79 | 302 |  |
|  |  | HR (95% CI) | Ref | 0.95 (0.68 - 1.31) | 0.97 (0.70 - 1.34) | 1.03 (0.74 - 1.43) | 1.01 (0.90 - 1.14) | 0.87 |
|  | Non-free sugars | |  |  |  |  |  |  |
|  |  | Cases, n (%) | 74 | 66 | 72 | 90 | 302 |  |
|  |  | HR (95% CI) | Ref | 0.85 (0.60 - 1.19) | 0.86 (0.61 - 1.22) | 0.94 (0.65 - 1.34) | 0.99 (0.88 - 1.11) | 0.88 |
|  | Fibre | |  |  |  |  |  |  |
|  |  | Cases, n (%) | 73 | 79 | 65 | 85 | 302 |  |
|  |  | HR (95% CI) | Ref | 1.07 (0.77 - 1.49) | 0.85 (0.59 - 1.22) | 1.02 (0.69 - 1.50) | 0.98 (0.86 - 1.12) | 0.78 |
| Models stratified by age at recruitment and sex, and adjusted for recruitment region, ethnicity, Townsend deprivation index, education, alcohol intake, smoking status, physical activity, menopausal status, BMI, SBP, SFA intake, and daily energy intake. Models were also adjusted for fruit and vegetable intake, excepting for models with total sugars, non-free sugars, and fibre as the exposure. Full details for each covariate are provided in the statistical analysis section in the main text.  ^a^ Carbohydrate intakes were expressed for each 5% of energy, excepting for fibre expressed for each 5 g/d.  ^b^ *P*-trend using continuous intakes with asterisks indicating statistical significance after using false discovery rate to correct for multiple testing. *P*-trend values ≥0.1 are displayed to two decimal places and *P*-trend values <0.1 are displayed to three decimal places.  *Abbreviations: AMI* acute myocardial infarction, *BMI* body mass index, *CI* confidence intervals, *CVD* cardiovascular disease, *g/d* grams per day, *HbA1c* glycated haemoglobin, *HDL-C* high-density lipoprotein cholesterol, *HR* hazard ratio, *IHD* ischaemic heart disease, *LDL-C* low-density lipoprotein cholesterol, *Q* quartile, *Ref* reference, *SBP* systolic blood pressure, *SFA* saturated fatty acid. | | | | | | | | |

#### **Table S10.** Hazard ratios (95% confidence intervals) for the associations between carbohydrate intakes and incidence of total CVD in 110,497 UK Biobank participants with adjustment for key cardiometabolic risk factors.

| **Models** | | | **Hazard ratios (95% CI)** | | | | | ***P*-trend^b^** | **χ2 (% change)^c^** |
| --- | --- | --- | --- | --- | --- | --- | --- | --- | --- |
|  |  |  | **Q1** | **Q2** | **Q3** | **Q4** | **For each 5%  of energy^a^** |  |  |
| Total carbohydrates | | |  |  |  |  |  |  |  |
|  | Mean intake (SD), % of energy | | 39.89 (4.57) | 47.41 (1.38) | 51.90 (1.30) | 58.27 (3.48) |  |  |  |
|  | Cases, n (%) | | 1,049 | 1,076 | 1,039 | 1,042 | 4188 |  |  |
|  | Minimally adjusted model^d^ | | Ref | 1.04 (0.95 - 1.13) | 1.02 (0.94 - 1.11) | 1.03 (0.94 - 1.12) | 1.01 (0.99 - 1.03) | 0.45 | 0.74 (Ref) |
|  | Multivariable adjusted model^e^ | | Ref | 1.05 (0.96 - 1.15) | 1.03 (0.94 - 1.14) | 1.01 (0.91 - 1.12) | 1.01 (0.98 - 1.03) | 0.66 | 1.65 (121.84) |
|  | Multivariable adjusted model with single adjustment for^f^ | | |  |  |  |  |  |  |
|  |  | BMI (final model) | Ref | 1.07 (0.98 - 1.17) | 1.06 (0.97 - 1.17) | 1.05 (0.95 - 1.16) | 1.02 (0.99 - 1.04) | 0.21 | 2.69 (262.43) |
|  |  | Waist circumference | Ref | 1.07 (0.98 - 1.17) | 1.06 (0.97 - 1.16) | 1.04 (0.94 - 1.15) | 1.01 (0.99 - 1.04) | 0.26 | 2.48 (233.72) |
|  |  | SBP | Ref | 1.06 (0.97 - 1.16) | 1.05 (0.96 - 1.15) | 1.02 (0.93 - 1.14) | 1.01 (0.99 - 1.04) | 0.42 | 2.14 (188.28) |
|  |  | LDL-C | Ref | 1.06 (0.97 - 1.16) | 1.05 (0.95 - 1.15) | 1.03 (0.93 - 1.14) | 1.01 (0.99 - 1.04) | 0.39 | 1.81 (143.91) |
|  |  | ApoB | Ref | 1.06 (0.97 - 1.16) | 1.05 (0.96 - 1.15) | 1.04 (0.93 - 1.15) | 1.01 (0.99 - 1.04) | 0.32 | 2.07 (178.65) |
|  |  | HDL-C | Ref | 1.03 (0.95 - 1.13) | 1.00 (0.92 - 1.10) | 0.97 (0.87 - 1.07) | 0.99 (0.97 - 1.02) | 0.62 | 1.83 (146.74) |
|  |  | Triglycerides | Ref | 1.04 (0.96 - 1.14) | 1.02 (0.93 - 1.12) | 0.99 (0.90 - 1.10) | 1.00 (0.98 - 1.03) | 0.93 | 1.48 (99.44) |
|  |  | HbA1c | Ref | 1.05 (0.96 - 1.15) | 1.04 (0.95 - 1.14) | 1.01 (0.92 - 1.12) | 1.01 (0.98 - 1.03) | 0.60 | 1.62 (117.76) |
| Refined grain starch | | |  |  |  |  |  |  |  |
|  | Mean intake (SD), % of energy | | 4.34 (1.94) | 9.08 (1.13) | 13.10 (1.26) | 19.90 (3.92) |  |  |  |
|  | Cases, n (%) | | 1,104 | 1,110 | 1,021 | 953 | 4188 |  |  |
|  | Minimally adjusted model^d^ | | Ref | 1.05 (0.97 - 1.15) | 1.03 (0.94 - 1.12) | 1.04 (0.96 - 1.14) | 1.01 (0.98 - 1.04) | 0.47 | 1.76 (Ref) |
|  | Multivariable adjusted model^e^ | | Ref | 1.05 (0.97 - 1.14) | 1.01 (0.93 - 1.11) | 1.00 (0.91 - 1.10) | 0.99 (0.97 - 1.02) | 0.69 | 1.69 (-4.44) |
|  | Multivariable adjusted model with single adjustment for^f^ | | |  |  |  |  |  |  |
|  |  | BMI (final model) | Ref | 1.05 (0.96 - 1.14) | 1.00 (0.92 - 1.10) | 0.99 (0.90 - 1.08) | 0.99 (0.96 - 1.02) | 0.45 | 2.06 (16.86) |
|  |  | Waist circumference | Ref | 1.05 (0.96 - 1.14) | 1.01 (0.92 - 1.10) | 0.99 (0.90 - 1.08) | 0.99 (0.96 - 1.02) | 0.46 | 2.01 (14.20) |
|  |  | SBP | Ref | 1.05 (0.97 - 1.14) | 1.01 (0.93 - 1.10) | 1.00 (0.91 - 1.10) | 0.99 (0.97 - 1.02) | 0.67 | 1.75 (-0.88) |
|  |  | LDL-C | Ref | 1.05 (0.96 - 1.14) | 1.01 (0.92 - 1.10) | 1.00 (0.91 - 1.10) | 0.99 (0.97 - 1.02) | 0.69 | 1.43 (-18.85) |
|  |  | ApoB | Ref | 1.05 (0.96 - 1.14) | 1.01 (0.92 - 1.10) | 1.00 (0.91 - 1.10) | 0.99 (0.97 - 1.02) | 0.69 | 1.44 (-18.20) |
|  |  | HDL-C | Ref | 1.04 (0.96 - 1.13) | 1.00 (0.92 - 1.09) | 0.98 (0.89 - 1.07) | 0.99 (0.96 - 1.01) | 0.38 | 2.04 (15.44) |
|  |  | Triglycerides | Ref | 1.05 (0.96 - 1.14) | 1.00 (0.92 - 1.10) | 0.99 (0.91 - 1.09) | 0.99 (0.97 - 1.02) | 0.56 | 1.75 (-0.93) |
|  |  | HbA1c | Ref | 1.05 (0.97 - 1.14) | 1.01 (0.93 - 1.10) | 1.00 (0.91 - 1.09) | 0.99 (0.97 - 1.02) | 0.61 | 1.80 (1.86) |
| Wholegrain starch | | |  |  |  |  |  |  |  |
|  | Mean intake (SD), % of energy | | 0.64 (0.79) | 3.48 (0.73) | 6.05 (0.81) | 10.87 (3.07) |  |  |  |
|  | Cases, n (%) | | 1,050 | 1,061 | 1,028 | 1,049 | 4188 |  |  |
|  | Minimally adjusted model^d^ | | Ref | 0.98 (0.90 - 1.07) | 0.93 (0.85 - 1.01) | 0.90 (0.83 - 0.98) | 0.95 (0.91 - 0.98) | <0.001* | 7.20 (Ref) |
|  | Multivariable adjusted model^e^ | | Ref | 1.02 (0.94 - 1.11) | 0.97 (0.89 - 1.06) | 0.95 (0.87 - 1.04) | 0.96 (0.93 - 1.00) | 0.049 | 3.01 (-58.20) |
|  | Multivariable adjusted model with single adjustment for^f^ | | |  |  |  |  |  |  |
|  |  | BMI (final model) | Ref | 1.03 (0.95 - 1.13) | 1.00 (0.91 - 1.09) | 0.99 (0.91 - 1.09) | 0.98 (0.95 - 1.02) | 0.39 | 1.07 (-85.17) |
|  |  | Waist circumference | Ref | 1.03 (0.95 - 1.13) | 1.00 (0.91 - 1.09) | 0.99 (0.90 - 1.08) | 0.98 (0.94 - 1.02) | 0.30 | 1.25 (-82.58) |
|  |  | SBP | Ref | 1.03 (0.95 - 1.13) | 0.99 (0.91 - 1.08) | 0.97 (0.89 - 1.06) | 0.97 (0.93 - 1.01) | 0.139 | 1.97 (-72.72) |
|  |  | LDL-C | Ref | 1.03 (0.95 - 1.13) | 0.98 (0.90 - 1.07) | 0.97 (0.89 - 1.06) | 0.97 (0.94 - 1.01) | 0.149 | 2.12 (-70.56) |
|  |  | ApoB | Ref | 1.04 (0.95 - 1.13) | 0.99 (0.91 - 1.08) | 0.98 (0.89 - 1.07) | 0.98 (0.94 - 1.01) | 0.21 | 1.85 (-74.34) |
|  |  | HDL-C | Ref | 1.03 (0.94 - 1.12) | 0.98 (0.90 - 1.07) | 0.96 (0.88 - 1.05) | 0.97 (0.93 - 1.01) | 0.088 | 2.30 (-68.11) |
|  |  | Triglycerides | Ref | 1.03 (0.94 - 1.12) | 0.98 (0.90 - 1.07) | 0.96 (0.88 - 1.05) | 0.97 (0.93 - 1.01) | 0.089 | 2.38 (-66.89) |
|  |  | HbA1c | Ref | 1.02 (0.94 - 1.12) | 0.98 (0.90 - 1.07) | 0.96 (0.87 - 1.05) | 0.96 (0.93 - 1.00) | 0.069 | 2.60 (-63.85) |
| Total sugars | | |  |  |  |  |  |  |  |
|  | Mean intake (SD), % of energy | | 16.65 (2.69) | 22.09 (1.15) | 26.14 (1.25) | 32.94 (4.11) |  |  |  |
|  | Cases, n (%) | | 1,034 | 1,004 | 1,072 | 1,078 | 4188 |  |  |
|  | Minimally adjusted model^d^ | | Ref | 0.94 (0.86 - 1.03) | 1.00 (0.92 - 1.09) | 1.04 (0.96 - 1.14) | 1.02 (1.00 - 1.05) | 0.087 | 5.41 (Ref) |
|  | Multivariable adjusted model^e^ | | Ref | 0.96 (0.88 - 1.05) | 1.02 (0.94 - 1.12) | 1.04 (0.95 - 1.14) | 1.02 (0.99 - 1.05) | 0.15 | 3.30 (-39.05) |
|  | Multivariable adjusted model with single adjustment for^f^ | | |  |  |  |  |  |  |
|  |  | BMI (final model) | Ref | 0.98 (0.89 - 1.07) | 1.04 (0.95 - 1.14) | 1.07 (0.97 - 1.17) | 1.03 (1.00 - 1.05) | 0.045 | 4.46 (-17.58) |
|  |  | Waist circumference | Ref | 0.97 (0.89 - 1.06) | 1.04 (0.95 - 1.14) | 1.06 (0.97 - 1.17) | 1.03 (1.00 - 1.05) | 0.050 | 4.32 (-20.08) |
|  |  | SBP | Ref | 0.97 (0.89 - 1.06) | 1.03 (0.94 - 1.12) | 1.05 (0.96 - 1.15) | 1.02 (1.00 - 1.05) | 0.10 | 3.59 (-33.66) |
|  |  | LDL-C | Ref | 0.97 (0.88 - 1.05) | 1.03 (0.94 - 1.12) | 1.05 (0.95 - 1.15) | 1.02 (1.00 - 1.05) | 0.10 | 3.55 (-34.40) |
|  |  | ApoB | Ref | 0.97 (0.89 - 1.06) | 1.03 (0.94 - 1.12) | 1.05 (0.96 - 1.15) | 1.02 (1.00 - 1.05) | 0.10 | 3.49 (-35.52) |
|  |  | HDL-C | Ref | 0.96 (0.88 - 1.04) | 1.01 (0.92 - 1.10) | 1.02 (0.93 - 1.12) | 1.01 (0.99 - 1.04) | 0.35 | 2.39 (-55.76) |
|  |  | Triglycerides | Ref | 0.96 (0.88 - 1.05) | 1.02 (0.93 - 1.11) | 1.04 (0.94 - 1.14) | 1.02 (0.99 - 1.04) | 0.19 | 2.86 (-47.11) |
|  |  | HbA1c | Ref | 0.96 (0.88 - 1.05) | 1.03 (0.94 - 1.12) | 1.05 (0.96 - 1.15) | 1.02 (1.00 - 1.05) | 0.11 | 3.70 (-31.56) |
| Free sugars | | |  |  |  |  |  |  |  |
|  | Mean intake (SD), % of energy | | 5.88 (1.66) | 9.51 (0.81) | 12.45 (0.93) | 17.92 (3.64) |  |  |  |
|  | Cases, n (%) | | 941 | 1,012 | 1,020 | 1,215 | 4188 |  |  |
|  | Minimally adjusted model^d^ | | Ref | 1.01 (0.92 - 1.10) | 0.97 (0.89 - 1.06) | 1.15 (1.05 - 1.25) | 1.08 (1.05 - 1.12) | <0.001* | 17.99 (Ref) |
|  | Multivariable adjusted model^e^ | | Ref | 1.02 (0.94 - 1.12) | 0.98 (0.90 - 1.07) | 1.11 (1.01 - 1.21) | 1.06 (1.03 - 1.09) | <0.001* | 9.31 (-48.27) |
|  | Multivariable adjusted model with single adjustment for^f^ | | |  |  |  |  |  |  |
|  |  | BMI (final model) | Ref | 1.04 (0.95 - 1.13) | 1.00 (0.91 - 1.09) | 1.13 (1.03 - 1.23) | 1.07 (1.03 - 1.10) | <0.001* | 10.61 (-41.03) |
|  |  | Waist circumference | Ref | 1.03 (0.95 - 1.13) | 0.99 (0.91 - 1.09) | 1.12 (1.02 - 1.23) | 1.06 (1.03 - 1.10) | <0.001* | 9.57 (-46.79) |
|  |  | SBP | Ref | 1.03 (0.94 - 1.12) | 0.98 (0.90 - 1.08) | 1.11 (1.01 - 1.21) | 1.06 (1.03 - 1.10) | <0.001* | 8.98 (-50.10) |
|  |  | LDL-C | Ref | 1.02 (0.94 - 1.12) | 0.98 (0.89 - 1.07) | 1.10 (1.01 - 1.21) | 1.06 (1.03 - 1.09) | <0.001* | 8.78 (-51.18) |
|  |  | ApoB | Ref | 1.03 (0.94 - 1.12) | 0.98 (0.89 - 1.07) | 1.10 (1.01 - 1.20) | 1.06 (1.02 - 1.09) | 0.001* | 8.38 (-53.42) |
|  |  | HDL-C | Ref | 1.02 (0.93 - 1.12) | 0.97 (0.89 - 1.07) | 1.09 (0.99 - 1.19) | 1.05 (1.02 - 1.09) | 0.002* | 7.01 (-61.03) |
|  |  | Triglycerides | Ref | 1.02 (0.93 - 1.12) | 0.97 (0.89 - 1.06) | 1.09 (0.99 - 1.19) | 1.05 (1.02 - 1.09) | 0.002* | 7.46 (-58.56) |
|  |  | HbA1c | Ref | 1.03 (0.94 - 1.12) | 0.98 (0.90 - 1.08) | 1.11 (1.02 - 1.22) | 1.06 (1.03 - 1.10) | <0.001* | 9.49 (-47.22) |
| Non-free sugars | | |  |  |  |  |  |  |  |
|  | Mean intake (SD), % of energy | | 6.80 (1.61) | 10.67 (0.92) | 14.07 (1.10) | 20.51 (4.09) |  |  |  |
|  | Cases, n (%) | | 1,152 | 1,050 | 1,003 | 983 | 4188 |  |  |
|  | Minimally adjusted model^d^ | | Ref | 0.90 (0.83 - 0.98) | 0.89 (0.82 - 0.97) | 0.92 (0.84 - 1.01) | 0.96 (0.93 - 0.99) | 0.010* | 8.50 (Ref) |
|  | Multivariable adjusted model^e^ | | Ref | 0.94 (0.86 - 1.02) | 0.93 (0.85 - 1.02) | 0.95 (0.86 - 1.04) | 0.96 (0.93 - 1.00) | 0.031 | 2.89 (-65.99) |
|  | Multivariable adjusted model with single adjustment for^f^ | | |  |  |  |  |  |  |
|  |  | BMI (final model) | Ref | 0.95 (0.87 - 1.04) | 0.95 (0.87 - 1.04) | 0.96 (0.87 - 1.06) | 0.97 (0.94 - 1.00) | 0.066 | 1.80 (-78.80) |
|  |  | Waist circumference | Ref | 0.96 (0.88 - 1.04) | 0.95 (0.87 - 1.04) | 0.97 (0.88 - 1.07) | 0.97 (0.94 - 1.01) | 0.10 | 1.52 (-82.11) |
|  |  | SBP | Ref | 0.95 (0.87 - 1.03) | 0.94 (0.86 - 1.03) | 0.96 (0.87 - 1.06) | 0.97 (0.94 - 1.00) | 0.055 | 2.17 (-74.45) |
|  |  | LDL-C | Ref | 0.95 (0.87 - 1.03) | 0.95 (0.86 - 1.04) | 0.96 (0.87 - 1.06) | 0.97 (0.94 - 1.00) | 0.085 | 1.98 (-76.75) |
|  |  | ApoB | Ref | 0.95 (0.87 - 1.04) | 0.95 (0.87 - 1.04) | 0.97 (0.88 - 1.07) | 0.97 (0.94 - 1.01) | 0.11 | 1.67 (-80.41) |
|  |  | HDL-C | Ref | 0.94 (0.86 - 1.02) | 0.93 (0.85 - 1.02) | 0.94 (0.86 - 1.04) | 0.96 (0.93 - 1.00) | 0.026 | 3.04 (-64.26) |
|  |  | Triglycerides | Ref | 0.95 (0.87 - 1.03) | 0.94 (0.86 - 1.03) | 0.96 (0.87 - 1.06) | 0.97 (0.94 - 1.00) | 0.077 | 2.24 (-73.61) |
|  |  | HbA1c | Ref | 0.94 (0.86 - 1.03) | 0.94 (0.86 - 1.03) | 0.95 (0.87 - 1.05) | 0.97 (0.94 - 1.00) | 0.048 | 2.51 (-70.45) |
| Fibre | | |  |  |  |  |  |  |  |
|  | Mean intake (SD), g/d | | 11.34 (2.07) | 11.34 (2.07) | 15.69 (0.95) | 19.11 (1.08) |  |  |  |
|  | Cases, n (%) | | 1,023 | 1,043 | 1,005 | 1,117 | 4188 |  |  |
|  | Minimally adjusted model^d^ | | Ref | 0.96 (0.88 - 1.04) | 0.88 (0.80 - 0.96) | 0.90 (0.83 - 0.98) | 0.95 (0.93 - 0.98) | <0.001 | 11.00 (Ref) |
|  | Multivariable adjusted model^e^ | | Ref | 0.98 (0.90 - 1.08) | 0.90 (0.82 - 0.99) | 0.90 (0.81 - 1.00) | 0.94 (0.91 - 0.97) | <0.001 | 7.20 (-34.58) |
|  | Multivariable adjusted model with single adjustment for^f^ | | |  |  |  |  |  |  |
|  |  | BMI (final model) | Ref | 0.99 (0.91 - 1.09) | 0.92 (0.84 - 1.02) | 0.94 (0.85 - 1.05) | 0.96 (0.93 - 0.99) | 0.014* | 3.68 (-66.55) |
|  |  | Waist circumference | Ref | 0.99 (0.91 - 1.09) | 0.92 (0.84 - 1.02) | 0.94 (0.85 - 1.04) | 0.96 (0.93 - 0.99) | 0.013* | 3.84 (-65.14) |
|  |  | SBP | Ref | 1.00 (0.91 - 1.09) | 0.91 (0.83 - 1.00) | 0.92 (0.83 - 1.02) | 0.95 (0.92 - 0.98) | 0.003* | 5.60 (-49.08) |
|  |  | LDL-C | Ref | 1.00 (0.91 - 1.09) | 0.92 (0.83 - 1.01) | 0.93 (0.84 - 1.03) | 0.95 (0.92 - 0.99) | 0.007* | 4.76 (-56.76) |
|  |  | ApoB | Ref | 1.00 (0.91 - 1.09) | 0.92 (0.84 - 1.02) | 0.94 (0.84 - 1.04) | 0.96 (0.92 - 0.99) | 0.011* | 4.15 (-62.27) |
|  |  | HDL-C | Ref | 0.98 (0.90 - 1.08) | 0.90 (0.82 - 0.99) | 0.90 (0.82 - 1.00) | 0.94 (0.91 - 0.98) | 0.001* | 6.60 (-40.05) |
|  |  | Triglycerides | Ref | 0.99 (0.90 - 1.08) | 0.91 (0.83 - 1.00) | 0.92 (0.83 - 1.02) | 0.95 (0.92 - 0.98) | 0.003* | 5.64 (-48.73) |
|  |  | HbA1c | Ref | 0.99 (0.90 - 1.08) | 0.90 (0.82 - 0.99) | 0.91 (0.82 - 1.01) | 0.94 (0.91 - 0.98) | 0.001* | 6.61 (-39.96) |
| ^a^ Carbohydrate intakes were expressed for each 5% of energy, excepting for fibre expressed for each 5 g/d.  ^b^ *P*-trend using continuous intakes with asterisks indicating statistical significance after using false discovery rate to correct for multiple testing. *P*-trend values ≥0.1 are displayed to two decimal places and *P*-trend values <0.1 are displayed to three decimal places.  ^c^ χ2 value for likelihood ratio test comparing model with and without the exposure and percentage change in χ2 value calculated with the minimally adjusted model as the reference group.  ^d^ Minimally adjusted model stratified by age at recruitment and sex and adjusted for recruitment region.  ^e^ Minimally adjusted model with additional adjustment for ethnicity, Townsend deprivation index, education, alcohol intake, smoking status, physical activity, menopausal status, SFA intake, and daily energy intake. Models were also adjusted for fruit and vegetable intake, excepting for models with total sugars, non-free sugars, and fibre as the exposure.  ^f^ Multivariable adjusted model with single adjustment for one of the following: BMI, waist circumference, SBP, LDL-C, ApoB, Triglycerides, HDL-C, or HbA1c. Models adjusting for LDL-C or ApoB were also adjusted for statin use. Full details for each covariate are provided in the statistical analysis section in the main text.  *Abbreviations: Apo* apolipoprotein, *BMI* body mass index, *CI* confidence intervals, *CVD* cardiovascular disease, *g/d* grams per day, *HbA1c* glycated haemoglobin, *HDL-C* high-density lipoprotein cholesterol, *HR* hazard ratio, *LDL-C* low-density lipoprotein cholesterol, *Q* quartile, *Ref* reference, *SBP* systolic blood pressure, *SFA* saturated fatty acid. | | | | | | | | | |

#### **Table S11.** Hazard ratios (95% confidence intervals) for the associations between carbohydrate intakes and incidence of IHD in 110,497 UK Biobank participants with adjustment for key cardiometabolic risk factors.

| **Models** | | | **Hazard ratios (95% CI)** | | | | | ***P*-trend^b^** | **χ2 (% change)^c^** |
| --- | --- | --- | --- | --- | --- | --- | --- | --- | --- |
|  |  |  | **Q1** | **Q2** | **Q3** | **Q4** | **For each 5%  of energy^a^** |  |  |
| Total carbohydrates | | |  |  |  |  |  |  |  |
|  | Mean intake (SD), % of energy | | 39.89 (4.57) | 47.41 (1.38) | 51.90 (1.30) | 58.27 (3.48) |  |  |  |
|  | Cases, n (%) | | 773 | 812 | 788 | 765 | 3138 |  |  |
|  | Minimally adjusted model^d^ | | Ref | 1.07 (0.97 - 1.18) | 1.06 (0.96 - 1.17) | 1.06 (0.96 - 1.17) | 1.01 (0.99 - 1.04) | 0.23 | 2.20 (Ref) |
|  | Multivariable adjusted model^e^ | | Ref | 1.08 (0.97 - 1.19) | 1.07 (0.96 - 1.19) | 1.03 (0.92 - 1.16) | 1.01 (0.98 - 1.04) | 0.46 | 2.60 (18.25) |
|  | Multivariable adjusted model with single adjustment for^f^ | | |  |  |  |  |  |  |
|  |  | BMI (final model) | Ref | 1.10 (0.99 - 1.21) | 1.11 (0.99 - 1.23) | 1.08 (0.96 - 1.22) | 1.02 (0.99 - 1.05) | 0.12 | 4.38 (99.38) |
|  |  | Waist circumference | Ref | 1.09 (0.99 - 1.21) | 1.10 (0.99 - 1.22) | 1.07 (0.95 - 1.21) | 1.02 (0.99 - 1.05) | 0.16 | 4.00 (82.11) |
|  |  | SBP | Ref | 1.09 (0.98 - 1.20) | 1.09 (0.98 - 1.21) | 1.05 (0.93 - 1.18) | 1.02 (0.99 - 1.05) | 0.29 | 3.38 (53.98) |
|  |  | LDL-C | Ref | 1.09 (0.98 - 1.20) | 1.09 (0.98 - 1.21) | 1.06 (0.94 - 1.20) | 1.02 (0.99 - 1.05) | 0.20 | 3.25 (48.10) |
|  |  | ApoB | Ref | 1.09 (0.99 - 1.21) | 1.09 (0.98 - 1.22) | 1.07 (0.95 - 1.20) | 1.02 (0.99 - 1.05) | 0.17 | 3.61 (64.24) |
|  |  | HDL-C | Ref | 1.05 (0.95 - 1.16) | 1.03 (0.93 - 1.15) | 0.98 (0.87 - 1.10) | 1.00 (0.97 - 1.03) | 0.78 | 2.06 (-6.35) |
|  |  | Triglycerides | Ref | 1.06 (0.96 - 1.18) | 1.06 (0.95 - 1.18) | 1.01 (0.90 - 1.14) | 1.01 (0.98 - 1.03) | 0.73 | 2.16 (-1.68) |
|  |  | HbA1c | Ref | 1.08 (0.97 - 1.19) | 1.07 (0.96 - 1.20) | 1.04 (0.92 - 1.17) | 1.01 (0.98 - 1.04) | 0.42 | 2.65 (20.54) |
| Refined grain starch | | |  |  |  |  |  |  |  |
|  | Mean intake (SD), % of energy | | 4.34 (1.94) | 9.08 (1.13) | 13.10 (1.26) | 19.90 (3.92) |  |  |  |
|  | Cases, n (%) | | 815 | 814 | 784 | 725 | 3138 |  |  |
|  | Minimally adjusted model^d^ | | Ref | 1.04 (0.95 - 1.15) | 1.06 (0.96 - 1.17) | 1.05 (0.95 - 1.16) | 1.02 (0.99 - 1.05) | 0.21 | 1.59 (Ref) |
|  | Multivariable adjusted model^e^ | | Ref | 1.03 (0.94 - 1.14) | 1.04 (0.94 - 1.14) | 1.00 (0.90 - 1.11) | 1.00 (0.97 - 1.03) | 0.93 | 0.91 (-42.53) |
|  | Multivariable adjusted model with single adjustment for^f^ | | |  |  |  |  |  |  |
|  |  | BMI (final model) | Ref | 1.03 (0.93 - 1.14) | 1.02 (0.93 - 1.13) | 0.98 (0.88 - 1.09) | 1.00 (0.97 - 1.03) | 0.79 | 1.10 (-30.72) |
|  |  | Waist circumference | Ref | 1.03 (0.93 - 1.14) | 1.03 (0.93 - 1.13) | 0.98 (0.89 - 1.09) | 1.00 (0.97 - 1.03) | 0.80 | 1.09 (-31.74) |
|  |  | SBP | Ref | 1.03 (0.94 - 1.14) | 1.03 (0.93 - 1.14) | 1.00 (0.90 - 1.11) | 1.00 (0.97 - 1.03) | 0.96 | 0.92 (-41.97) |
|  |  | LDL-C | Ref | 1.03 (0.93 - 1.13) | 1.03 (0.93 - 1.14) | 1.00 (0.90 - 1.11) | 1.00 (0.97 - 1.03) | 0.91 | 0.60 (-62.17) |
|  |  | ApoB | Ref | 1.03 (0.93 - 1.13) | 1.03 (0.93 - 1.14) | 1.00 (0.90 - 1.11) | 1.00 (0.97 - 1.03) | 0.93 | 0.61 (-61.94) |
|  |  | HDL-C | Ref | 1.02 (0.93 - 1.13) | 1.02 (0.92 - 1.12) | 0.97 (0.88 - 1.08) | 0.99 (0.96 - 1.02) | 0.67 | 1.11 (-29.96) |
|  |  | Triglycerides | Ref | 1.03 (0.93 - 1.14) | 1.02 (0.93 - 1.13) | 0.99 (0.89 - 1.10) | 1.00 (0.97 - 1.03) | 0.91 | 0.79 (-50.38) |
|  |  | HbA1c | Ref | 1.03 (0.94 - 1.14) | 1.03 (0.93 - 1.14) | 0.99 (0.90 - 1.11) | 1.00 (0.97 - 1.03) | 0.97 | 0.95 (-40.07) |
| Wholegrain starch | | |  |  |  |  |  |  |  |
|  | Mean intake (SD), % of energy | | 0.64 (0.79) | 3.48 (0.73) | 6.05 (0.81) | 10.87 (3.07) |  |  |  |
|  | Cases, n (%) | | 787 | 794 | 753 | 804 | 3138 |  |  |
|  | Minimally adjusted model^d^ | | Ref | 0.99 (0.90 - 1.10) | 0.92 (0.83 - 1.01) | 0.93 (0.84 - 1.03) | 0.94 (0.90 - 0.98) | 0.01 | 4.53 (Ref) |
|  | Multivariable adjusted model^e^ | | Ref | 1.03 (0.93 - 1.14) | 0.96 (0.87 - 1.07) | 0.98 (0.88 - 1.09) | 0.96 (0.92 - 1.00) | 0.07 | 1.74 (-61.46) |
|  | Multivariable adjusted model with single adjustment for^f^ | | |  |  |  |  |  |  |
|  |  | BMI (final model) | Ref | 1.04 (0.94 - 1.15) | 0.99 (0.90 - 1.10) | 1.03 (0.93 - 1.15) | 0.98 (0.94 - 1.03) | 0.49 | 1.39 (-69.35) |
|  |  | Waist circumference | Ref | 1.04 (0.94 - 1.15) | 0.99 (0.89 - 1.10) | 1.03 (0.93 - 1.14) | 0.98 (0.94 - 1.03) | 0.39 | 1.28 (-71.73) |
|  |  | SBP | Ref | 1.04 (0.94 - 1.15) | 0.98 (0.88 - 1.08) | 1.00 (0.90 - 1.11) | 0.97 (0.93 - 1.01) | 0.162 | 1.35 (-70.14) |
|  |  | LDL-C | Ref | 1.04 (0.94 - 1.15) | 0.98 (0.88 - 1.08) | 1.01 (0.91 - 1.12) | 0.97 (0.93 - 1.02) | 0.23 | 1.63 (-64.03) |
|  |  | ApoB | Ref | 1.05 (0.95 - 1.16) | 0.99 (0.89 - 1.09) | 1.02 (0.92 - 1.13) | 0.98 (0.94 - 1.02) | 0.32 | 1.51 (-66.75) |
|  |  | HDL-C | Ref | 1.03 (0.93 - 1.14) | 0.97 (0.88 - 1.08) | 0.99 (0.90 - 1.10) | 0.97 (0.92 - 1.01) | 0.126 | 1.39 (-69.39) |
|  |  | Triglycerides | Ref | 1.03 (0.94 - 1.14) | 0.97 (0.88 - 1.08) | 1.00 (0.90 - 1.10) | 0.97 (0.92 - 1.01) | 0.131 | 1.40 (-69.01) |
|  |  | HbA1c | Ref | 1.03 (0.93 - 1.14) | 0.97 (0.88 - 1.08) | 0.99 (0.89 - 1.09) | 0.96 (0.92 - 1.01) | 0.095 | 1.47 (-67.47) |
| Total sugars | | |  |  |  |  |  |  |  |
|  | Mean intake (SD), % of energy | | 16.65 (2.69) | 22.09 (1.15) | 26.14 (1.25) | 32.94 (4.11) |  |  |  |
|  | Cases, n (%) | | 774 | 754 | 828 | 782 | 3138 |  |  |
|  | Minimally adjusted model^d^ | | Ref | 0.96 (0.86 - 1.06) | 1.06 (0.96 - 1.17) | 1.05 (0.95 - 1.16) | 1.02 (0.99 - 1.05) | 0.13 | 5.02 (Ref) |
|  | Multivariable adjusted model^e^ | | Ref | 0.97 (0.88 - 1.08) | 1.07 (0.97 - 1.19) | 1.04 (0.94 - 1.16) | 1.02 (0.99 - 1.05) | 0.20 | 4.22 (-16.07) |
|  | Multivariable adjusted model with single adjustment for^f^ | | |  |  |  |  |  |  |
|  |  | BMI (final model) | Ref | 0.99 (0.89 - 1.10) | 1.10 (0.99 - 1.21) | 1.07 (0.96 - 1.19) | 1.03 (1.00 - 1.06) | 0.069 | 5.67 (12.81) |
|  |  | Waist circumference | Ref | 0.99 (0.89 - 1.09) | 1.10 (0.99 - 1.21) | 1.07 (0.96 - 1.19) | 1.03 (1.00 - 1.06) | 0.077 | 5.58 (11.08) |
|  |  | SBP | Ref | 0.98 (0.89 - 1.08) | 1.08 (0.98 - 1.20) | 1.05 (0.95 - 1.17) | 1.02 (0.99 - 1.05) | 0.15 | 4.62 (-8.07) |
|  |  | LDL-C | Ref | 0.98 (0.88 - 1.08) | 1.08 (0.98 - 1.20) | 1.05 (0.95 - 1.17) | 1.02 (0.99 - 1.05) | 0.13 | 4.67 (-6.98) |
|  |  | ApoB | Ref | 0.98 (0.89 - 1.08) | 1.08 (0.98 - 1.20) | 1.05 (0.95 - 1.17) | 1.02 (0.99 - 1.05) | 0.13 | 4.69 (-6.69) |
|  |  | HDL-C | Ref | 0.96 (0.87 - 1.07) | 1.05 (0.95 - 1.17) | 1.02 (0.91 - 1.13) | 1.01 (0.98 - 1.04) | 0.48 | 3.18 (-36.75) |
|  |  | Triglycerides | Ref | 0.97 (0.88 - 1.08) | 1.07 (0.96 - 1.18) | 1.04 (0.93 - 1.15) | 1.02 (0.99 - 1.05) | 0.28 | 3.55 (-29.38) |
|  |  | HbA1c | Ref | 0.98 (0.88 - 1.08) | 1.08 (0.98 - 1.19) | 1.05 (0.95 - 1.17) | 1.02 (0.99 - 1.05) | 0.16 | 4.65 (-7.39) |
| Free sugars | | |  |  |  |  |  |  |  |
|  | Mean intake (SD), % of energy | | 5.88 (1.66) | 9.51 (0.81) | 12.45 (0.93) | 17.92 (3.64) |  |  |  |
|  | Cases, n (%) | | 707 | 749 | 778 | 904 | 3138 |  |  |
|  | Minimally adjusted model^d^ | | Ref | 0.98 (0.89 - 1.09) | 0.97 (0.88 - 1.08) | 1.10 (0.99 - 1.21) | 1.07 (1.04 - 1.11) | <0.001* | 7.96 (Ref) |
|  | Multivariable adjusted model^e^ | | Ref | 0.99 (0.90 - 1.10) | 0.97 (0.88 - 1.08) | 1.05 (0.95 - 1.17) | 1.05 (1.01 - 1.09) | 0.010* | 2.81 (-64.69) |
|  | Multivariable adjusted model with single adjustment for^f^ | | |  |  |  |  |  |  |
|  |  | BMI (final model) | Ref | 1.01 (0.91 - 1.12) | 0.99 (0.89 - 1.10) | 1.07 (0.97 - 1.19) | 1.06 (1.02 - 1.10) | 0.003* | 3.30 (-58.52) |
|  |  | Waist circumference | Ref | 1.00 (0.90 - 1.11) | 0.99 (0.89 - 1.09) | 1.06 (0.96 - 1.18) | 1.05 (1.02 - 1.09) | 0.005* | 2.76 (-65.30) |
|  |  | SBP | Ref | 1.00 (0.90 - 1.10) | 0.98 (0.88 - 1.08) | 1.05 (0.95 - 1.17) | 1.05 (1.01 - 1.09) | 0.009* | 2.63 (-66.90) |
|  |  | LDL-C | Ref | 0.99 (0.89 - 1.10) | 0.97 (0.87 - 1.07) | 1.05 (0.94 - 1.16) | 1.05 (1.01 - 1.09) | 0.015* | 2.59 (-67.44) |
|  |  | ApoB | Ref | 0.99 (0.90 - 1.10) | 0.97 (0.87 - 1.07) | 1.04 (0.94 - 1.16) | 1.04 (1.01 - 1.08) | 0.019* | 2.34 (-70.55) |
|  |  | HDL-C | Ref | 0.99 (0.89 - 1.10) | 0.96 (0.87 - 1.07) | 1.03 (0.93 - 1.14) | 1.04 (1.00 - 1.08) | 0.048 | 1.68 (-78.85) |
|  |  | Triglycerides | Ref | 0.99 (0.89 - 1.09) | 0.96 (0.86 - 1.06) | 1.03 (0.92 - 1.14) | 1.04 (1.00 - 1.08) | 0.043 | 1.95 (-75.53) |
|  |  | HbA1c | Ref | 1.00 (0.90 - 1.11) | 0.98 (0.88 - 1.08) | 1.06 (0.95 - 1.17) | 1.05 (1.01 - 1.09) | 0.009* | 2.87 (-63.90) |
| Non-free sugars | | |  |  |  |  |  |  |  |
|  | Mean intake (SD), % of energy | | 6.80 (1.61) | 10.67 (0.92) | 14.07 (1.10) | 20.51 (4.09) |  |  |  |
|  | Cases, n (%) | | 882 | 791 | 746 | 719 | 3138 |  |  |
|  | Minimally adjusted model^d^ | | Ref | 0.91 (0.83 - 1.00) | 0.91 (0.82 - 1.00) | 0.95 (0.86 - 1.05) | 0.97 (0.94 - 1.00) | 0.073 | 4.81 (Ref) |
|  | Multivariable adjusted model^e^ | | Ref | 0.95 (0.86 - 1.05) | 0.95 (0.86 - 1.06) | 0.98 (0.88 - 1.10) | 0.98 (0.94 - 1.01) | 0.20 | 1.55 (-67.71) |
|  | Multivariable adjusted model with single adjustment for^f^ | | |  |  |  |  |  |  |
|  |  | BMI (final model) | Ref | 0.96 (0.87 - 1.06) | 0.97 (0.87 - 1.08) | 1.00 (0.90 - 1.12) | 0.98 (0.95 - 1.02) | 0.34 | 1.06 (-77.87) |
|  |  | Waist circumference | Ref | 0.97 (0.88 - 1.07) | 0.98 (0.88 - 1.08) | 1.01 (0.91 - 1.14) | 0.99 (0.95 - 1.02) | 0.44 | 1.06 (-77.86) |
|  |  | SBP | Ref | 0.96 (0.87 - 1.05) | 0.96 (0.87 - 1.07) | 1.00 (0.89 - 1.11) | 0.98 (0.94 - 1.02) | 0.29 | 1.25 (-74.06) |
|  |  | LDL-C | Ref | 0.96 (0.87 - 1.06) | 0.97 (0.88 - 1.08) | 1.01 (0.90 - 1.13) | 0.99 (0.95 - 1.02) | 0.46 | 1.27 (-73.58) |
|  |  | ApoB | Ref | 0.97 (0.88 - 1.07) | 0.98 (0.88 - 1.08) | 1.02 (0.91 - 1.14) | 0.99 (0.95 - 1.03) | 0.53 | 1.15 (-76.17) |
|  |  | HDL-C | Ref | 0.95 (0.86 - 1.04) | 0.95 (0.86 - 1.05) | 0.98 (0.88 - 1.10) | 0.97 (0.94 - 1.01) | 0.18 | 1.61 (-66.48) |
|  |  | Triglycerides | Ref | 0.96 (0.87 - 1.06) | 0.96 (0.87 - 1.07) | 1.01 (0.90 - 1.13) | 0.98 (0.95 - 1.02) | 0.40 | 1.46 (-69.72) |
|  |  | HbA1c | Ref | 0.95 (0.86 - 1.05) | 0.96 (0.86 - 1.06) | 0.99 (0.89 - 1.11) | 0.98 (0.94 - 1.02) | 0.27 | 1.48 (-69.13) |
| Fibre | | |  |  |  |  |  |  |  |
|  | Mean intake (SD), g/d | | 11.34 (2.07) | 11.34 (2.07) | 15.69 (0.95) | 19.11 (1.08) |  |  |  |
|  | Cases, n (%) | | 759 | 793 | 752 | 834 | 3138 |  |  |
|  | Minimally adjusted model^d^ | | Ref | 0.99 (0.89 - 1.09) | 0.89 (0.80 - 0.98) | 0.90 (0.82 - 1.00) | 0.95 (0.92 - 0.98) | 0.003* | 8.30 (Ref) |
|  | Multivariable adjusted model^e^ | | Ref | 1.01 (0.91 - 1.12) | 0.91 (0.81 - 1.01) | 0.89 (0.79 - 1.01) | 0.94 (0.90 - 0.98) | 0.002* | 7.41 (-10.70) |
|  | Multivariable adjusted model with single adjustment for^f^ | | |  |  |  |  |  |  |
|  |  | BMI (final model) | Ref | 1.02 (0.92 - 1.13) | 0.93 (0.84 - 1.04) | 0.94 (0.83 - 1.06) | 0.96 (0.92 - 1.00) | 0.036 | 3.70 (-55.40) |
|  |  | Waist circumference | Ref | 1.03 (0.92 - 1.14) | 0.93 (0.84 - 1.04) | 0.94 (0.83 - 1.06) | 0.96 (0.92 - 1.00) | 0.032 | 3.99 (-51.96) |
|  |  | SBP | Ref | 1.02 (0.92 - 1.14) | 0.92 (0.82 - 1.03) | 0.91 (0.81 - 1.03) | 0.95 (0.91 - 0.99) | 0.007* | 6.11 (-26.39) |
|  |  | LDL-C | Ref | 1.03 (0.93 - 1.14) | 0.93 (0.83 - 1.04) | 0.93 (0.83 - 1.05) | 0.96 (0.92 - 1.00) | 0.028 | 4.52 (-45.56) |
|  |  | ApoB | Ref | 1.03 (0.93 - 1.14) | 0.94 (0.84 - 1.05) | 0.94 (0.84 - 1.06) | 0.96 (0.92 - 1.00) | 0.039 | 4.06 (-51.10) |
|  |  | HDL-C | Ref | 1.01 (0.91 - 1.12) | 0.91 (0.81 - 1.02) | 0.90 (0.80 - 1.01) | 0.94 (0.91 - 0.98) | 0.003* | 6.70 (-19.22) |
|  |  | Triglycerides | Ref | 1.02 (0.92 - 1.13) | 0.92 (0.82 - 1.03) | 0.92 (0.81 - 1.03) | 0.95 (0.91 - 0.99) | 0.009 | 5.61 (-32.34) |
|  |  | HbA1c | Ref | 1.01 (0.91 - 1.13) | 0.91 (0.82 - 1.02) | 0.90 (0.80 - 1.02) | 0.94 (0.91 - 0.98) | 0.003* | 6.84 (-17.58) |
| ^a^ Carbohydrate intakes were expressed for each 5% of energy, excepting for fibre expressed for each 5 g/d.  ^b^ *P*-trend using continuous intakes with asterisks indicating statistical significance after using false discovery rate to correct for multiple testing. *P*-trend values ≥0.1 are displayed to two decimal places and *P*-trend values <0.1 are displayed to three decimal places.  ^c^ χ2 value for likelihood ratio test comparing model with and without the exposure and percentage change in χ2 value calculated with the minimally adjusted model as the reference group.  ^d^ Minimally adjusted model stratified by age at recruitment and sex and adjusted for recruitment region.  ^e^ Minimally adjusted model with additional adjustment for ethnicity, Townsend deprivation index, education, alcohol intake, smoking status, physical activity, menopausal status, SFA intake, and daily energy intake. Models were also adjusted for fruit and vegetable intake, excepting for models with total sugars, non-free sugars, and fibre as the exposure.  ^f^ Multivariable adjusted model with single adjustment for one of the following: BMI, waist circumference, SBP, LDL-C, ApoB, Triglycerides, HDL-C, or HbA1c. Models adjusting for LDL-C or ApoB were also adjusted for statin use. Full details for each covariate are provided in the statistical analysis section in the main text.  *Abbreviations: Apo* apolipoprotein, *BMI* body mass index, *CI* confidence intervals, *g/d* grams per day, *HbA1c* glycated haemoglobin, *HDL-C* high-density lipoprotein cholesterol, HR hazard ratio, *IHD* ischaemic heart disease, *LDL-C* low-density lipoprotein cholesterol, *Q* quartile, *Ref* reference, *SBP* systolic blood pressure, *SFA* saturated fatty acid. | | | | | | | | | |

#### **Table S12.** Hazard ratios (95% confidence intervals) for the associations between carbohydrate intakes and incidence of total stroke in 110,497 UK Biobank participants with adjustment for key cardiometabolic risk factors.

| **Models** | | | **Hazard ratios (95% CI)** | | | | | ***P*-trend^b^** | **χ2 (% change)^c^** |
| --- | --- | --- | --- | --- | --- | --- | --- | --- | --- |
|  |  |  | **Q1** | **Q2** | **Q3** | **Q4** | **For each 5%  of energy^a^** |  |  |
| Total carbohydrates | | |  |  |  |  |  |  |  |
|  | Mean intake (SD), % of energy | | 39.89 (4.57) | 47.41 (1.38) | 51.90 (1.30) | 58.27 (3.48) |  |  |  |
|  | Cases, n (%) | | 294 | 285 | 268 | 277 | 1124 |  |  |
|  | Minimally adjusted model^d^ | | Ref | 0.96 (0.81 - 1.13) | 0.90 (0.76 - 1.06) | 0.95 (0.80 - 1.12) | 0.99 (0.95 - 1.03) | 0.50 | 1.54 (Ref) |
|  | Multivariable adjusted model^e^ | | Ref | 1.00 (0.84 - 1.18) | 0.94 (0.79 - 1.12) | 0.95 (0.78 - 1.15) | 0.99 (0.94 - 1.04) | 0.66 | 0.69 (-55.17) |
|  | Multivariable adjusted model with single adjustment for^f^ | | |  |  |  |  |  |  |
|  |  | BMI (final model) | Ref | 1.00 (0.85 - 1.19) | 0.95 (0.80 - 1.14) | 0.97 (0.79 - 1.18) | 0.99 (0.95 - 1.04) | 0.82 | 0.46 (-70.07) |
|  |  | Waist circumference | Ref | 1.00 (0.85 - 1.19) | 0.95 (0.80 - 1.14) | 0.96 (0.79 - 1.17) | 0.99 (0.95 - 1.04) | 0.81 | 0.47 (-69.08) |
|  |  | SBP | Ref | 1.00 (0.85 - 1.19) | 0.95 (0.80 - 1.14) | 0.96 (0.79 - 1.17) | 0.99 (0.95 - 1.04) | 0.78 | 0.49 (-68.07) |
|  |  | LDL-C | Ref | 1.00 (0.84 - 1.18) | 0.94 (0.78 - 1.12) | 0.95 (0.78 - 1.15) | 0.99 (0.94 - 1.04) | 0.64 | 0.74 (-51.64) |
|  |  | ApoB | Ref | 1.00 (0.84 - 1.18) | 0.94 (0.79 - 1.12) | 0.95 (0.78 - 1.16) | 0.99 (0.94 - 1.04) | 0.67 | 0.69 (-54.78) |
|  |  | HDL-C | Ref | 0.99 (0.84 - 1.17) | 0.93 (0.78 - 1.12) | 0.94 (0.77 - 1.14) | 0.99 (0.94 - 1.03) | 0.57 | 0.84 (-45.21) |
|  |  | Triglycerides | Ref | 0.99 (0.84 - 1.17) | 0.94 (0.78 - 1.12) | 0.94 (0.77 - 1.15) | 0.99 (0.94 - 1.04) | 0.62 | 0.74 (-52.06) |
|  |  | HbA1c | Ref | 0.99 (0.84 - 1.18) | 0.94 (0.79 - 1.13) | 0.95 (0.78 - 1.16) | 0.99 (0.94 - 1.04) | 0.68 | 0.61 (-60.51) |
| Refined grain starch | | |  |  |  |  |  |  |  |
|  | Mean intake (SD), % of energy | | 4.34 (1.94) | 9.08 (1.13) | 13.10 (1.26) | 19.90 (3.92) |  |  |  |
|  | Cases, n (%) | | 304 | 313 | 260 | 247 | 1124 |  |  |
|  | Minimally adjusted model^d^ | | Ref | 1.09 (0.93 - 1.28) | 0.99 (0.84 - 1.17) | 1.06 (0.89 - 1.25) | 0.99 (0.94 - 1.04) | 0.70 | 1.95 (Ref) |
|  | Multivariable adjusted model^e^ | | Ref | 1.10 (0.94 - 1.29) | 0.99 (0.84 - 1.17) | 1.04 (0.87 - 1.24) | 0.98 (0.93 - 1.03) | 0.50 | 2.04 (4.51) |
|  | Multivariable adjusted model with single adjustment for^f^ | | |  |  |  |  |  |  |
|  |  | BMI (final model) | Ref | 1.10 (0.94 - 1.29) | 0.99 (0.83 - 1.17) | 1.03 (0.86 - 1.22) | 0.98 (0.93 - 1.03) | 0.43 | 2.09 (7.24) |
|  |  | Waist circumference | Ref | 1.10 (0.94 - 1.29) | 0.99 (0.83 - 1.17) | 1.03 (0.86 - 1.23) | 0.98 (0.93 - 1.03) | 0.44 | 2.09 (7.20) |
|  |  | SBP | Ref | 1.10 (0.94 - 1.29) | 0.99 (0.83 - 1.17) | 1.04 (0.87 - 1.24) | 0.98 (0.93 - 1.04) | 0.51 | 2.08 (6.69) |
|  |  | LDL-C | Ref | 1.10 (0.94 - 1.29) | 0.99 (0.83 - 1.17) | 1.03 (0.87 - 1.23) | 0.98 (0.93 - 1.03) | 0.48 | 2.03 (4.12) |
|  |  | ApoB | Ref | 1.10 (0.94 - 1.29) | 0.99 (0.83 - 1.17) | 1.04 (0.87 - 1.24) | 0.98 (0.93 - 1.03) | 0.49 | 2.03 (4.10) |
|  |  | HDL-C | Ref | 1.10 (0.94 - 1.29) | 0.99 (0.83 - 1.17) | 1.03 (0.86 - 1.23) | 0.98 (0.93 - 1.03) | 0.46 | 2.03 (4.01) |
|  |  | Triglycerides | Ref | 1.10 (0.94 - 1.29) | 0.99 (0.84 - 1.17) | 1.04 (0.87 - 1.24) | 0.98 (0.93 - 1.03) | 0.49 | 2.02 (3.56) |
|  |  | HbA1c | Ref | 1.10 (0.94 - 1.29) | 0.99 (0.83 - 1.17) | 1.03 (0.87 - 1.23) | 0.98 (0.93 - 1.03) | 0.48 | 2.05 (4.81) |
| Wholegrain starch | | |  |  |  |  |  |  |  |
|  | Mean intake (SD), % of energy | | 0.64 (0.79) | 3.48 (0.73) | 6.05 (0.81) | 10.87 (3.07) |  |  |  |
|  | Cases, n (%) | | 286 | 284 | 292 | 262 | 1124 |  |  |
|  | Minimally adjusted model^d^ | | Ref | 0.94 (0.80 - 1.11) | 0.93 (0.79 - 1.10) | 0.82 (0.69 - 0.97) | 0.94 (0.88 - 1.01) | 0.117 | 5.89 (Ref) |
|  | Multivariable adjusted model^e^ | | Ref | 0.98 (0.83 - 1.16) | 0.98 (0.83 - 1.16) | 0.86 (0.72 - 1.02) | 0.96 (0.89 - 1.03) | 0.28 | 4.03 (-31.49) |
|  | Multivariable adjusted model with single adjustment for^f^ | | |  |  |  |  |  |  |
|  |  | BMI (final model) | Ref | 0.99 (0.84 - 1.17) | 1.00 (0.84 - 1.18) | 0.88 (0.74 - 1.05) | 0.97 (0.90 - 1.05) | 0.44 | 3.14 (-46.73) |
|  |  | Waist circumference | Ref | 0.99 (0.84 - 1.17) | 1.00 (0.84 - 1.18) | 0.88 (0.73 - 1.04) | 0.97 (0.90 - 1.05) | 0.42 | 3.20 (-45.68) |
|  |  | SBP | Ref | 0.99 (0.84 - 1.17) | 1.00 (0.84 - 1.18) | 0.87 (0.73 - 1.04) | 0.97 (0.90 - 1.04) | 0.41 | 3.31 (-43.75) |
|  |  | LDL-C | Ref | 0.98 (0.83 - 1.16) | 0.98 (0.83 - 1.16) | 0.86 (0.72 - 1.02) | 0.96 (0.89 - 1.03) | 0.28 | 4.07 (-30.81) |
|  |  | ApoB | Ref | 0.99 (0.83 - 1.16) | 0.98 (0.83 - 1.16) | 0.86 (0.72 - 1.02) | 0.96 (0.89 - 1.04) | 0.29 | 4.03 (-31.60) |
|  |  | HDL-C | Ref | 0.98 (0.83 - 1.16) | 0.98 (0.83 - 1.16) | 0.86 (0.72 - 1.02) | 0.96 (0.89 - 1.04) | 0.30 | 3.92 (-33.35) |
|  |  | Triglycerides | Ref | 0.98 (0.83 - 1.16) | 0.98 (0.83 - 1.16) | 0.85 (0.72 - 1.02) | 0.96 (0.89 - 1.03) | 0.27 | 4.10 (-30.39) |
|  |  | HbA1c | Ref | 0.99 (0.84 - 1.16) | 0.99 (0.83 - 1.17) | 0.86 (0.72 - 1.02) | 0.96 (0.89 - 1.04) | 0.30 | 3.93 (-33.31) |
| Total sugars | | |  |  |  |  |  |  |  |
|  | Mean intake (SD), % of energy | | 16.65 (2.69) | 22.09 (1.15) | 26.14 (1.25) | 32.94 (4.11) |  |  |  |
|  | Cases, n (%) | | 275 | 270 | 264 | 315 | 1124 |  |  |
|  | Minimally adjusted model^d^ | | Ref | 0.92 (0.78 - 1.09) | 0.87 (0.73 - 1.03) | 1.02 (0.87 - 1.20) | 1.02 (0.97 - 1.07) | 0.46 | 5.05 (Ref) |
|  | Multivariable adjusted model^e^ | | Ref | 0.95 (0.80 - 1.13) | 0.90 (0.75 - 1.07) | 1.03 (0.87 - 1.23) | 1.02 (0.97 - 1.07) | 0.45 | 3.15 (-37.59) |
|  | Multivariable adjusted model with single adjustment for^f^ | | |  |  |  |  |  |  |
|  |  | BMI (final model) | Ref | 0.96 (0.81 - 1.13) | 0.91 (0.76 - 1.08) | 1.05 (0.88 - 1.25) | 1.02 (0.97 - 1.08) | 0.35 | 3.13 (-37.99) |
|  |  | Waist circumference | Ref | 0.96 (0.81 - 1.14) | 0.91 (0.76 - 1.08) | 1.05 (0.88 - 1.25) | 1.02 (0.97 - 1.08) | 0.35 | 3.14 (-37.83) |
|  |  | SBP | Ref | 0.95 (0.80 - 1.13) | 0.90 (0.76 - 1.07) | 1.04 (0.87 - 1.24) | 1.02 (0.97 - 1.07) | 0.41 | 3.17 (-37.16) |
|  |  | LDL-C | Ref | 0.95 (0.80 - 1.13) | 0.90 (0.75 - 1.07) | 1.03 (0.87 - 1.23) | 1.02 (0.97 - 1.07) | 0.46 | 3.19 (-36.86) |
|  |  | ApoB | Ref | 0.95 (0.80 - 1.13) | 0.90 (0.75 - 1.07) | 1.03 (0.87 - 1.23) | 1.02 (0.97 - 1.07) | 0.45 | 3.21 (-36.44) |
|  |  | HDL-C | Ref | 0.95 (0.80 - 1.12) | 0.89 (0.75 - 1.07) | 1.03 (0.86 - 1.23) | 1.02 (0.97 - 1.07) | 0.49 | 3.13 (-38.01) |
|  |  | Triglycerides | Ref | 0.95 (0.80 - 1.12) | 0.90 (0.75 - 1.07) | 1.03 (0.87 - 1.23) | 1.02 (0.97 - 1.07) | 0.46 | 3.24 (-35.84) |
|  |  | HbA1c | Ref | 0.95 (0.80 - 1.13) | 0.90 (0.76 - 1.07) | 1.04 (0.87 - 1.24) | 1.02 (0.97 - 1.07) | 0.41 | 3.13 (-38.00) |
| Free sugars | | |  |  |  |  |  |  |  |
|  | Mean intake (SD), % of energy | | 5.88 (1.66) | 9.51 (0.81) | 12.45 (0.93) | 17.92 (3.64) |  |  |  |
|  | Cases, n (%) | | 247 | 283 | 257 | 337 | 1124 |  |  |
|  | Minimally adjusted model^d^ | | Ref | 1.11 (0.94 - 1.32) | 0.98 (0.82 - 1.17) | 1.32 (1.12 - 1.56) | 1.11 (1.05 - 1.18) | <0.001* | 16.50 (Ref) |
|  | Multivariable adjusted model^e^ | | Ref | 1.14 (0.96 - 1.35) | 1.01 (0.84 - 1.20) | 1.31 (1.10 - 1.56) | 1.10 (1.03 - 1.17) | 0.003* | 13.35 (-19.13) |
|  | Multivariable adjusted model with single adjustment for^f^ | | |  |  |  |  |  |  |
|  |  | BMI (final model) | Ref | 1.15 (0.97 - 1.36) | 1.02 (0.85 - 1.22) | 1.33 (1.12 - 1.58) | 1.10 (1.04 - 1.17) | 0.002* | 14.02 (-15.03) |
|  |  | Waist circumference | Ref | 1.15 (0.96 - 1.36) | 1.02 (0.85 - 1.22) | 1.32 (1.11 - 1.57) | 1.10 (1.03 - 1.17) | 0.002* | 13.75 (-16.70) |
|  |  | SBP | Ref | 1.14 (0.96 - 1.35) | 1.01 (0.84 - 1.20) | 1.31 (1.10 - 1.55) | 1.10 (1.03 - 1.17) | 0.004* | 13.12 (-20.50) |
|  |  | LDL-C | Ref | 1.14 (0.96 - 1.35) | 1.01 (0.84 - 1.20) | 1.31 (1.10 - 1.56) | 1.10 (1.03 - 1.17) | 0.003* | 13.40 (-18.83) |
|  |  | ApoB | Ref | 1.14 (0.96 - 1.35) | 1.01 (0.84 - 1.20) | 1.31 (1.10 - 1.56) | 1.10 (1.03 - 1.17) | 0.003* | 13.34 (-19.20) |
|  |  | HDL-C | Ref | 1.14 (0.96 - 1.35) | 1.01 (0.84 - 1.20) | 1.30 (1.10 - 1.55) | 1.09 (1.03 - 1.16) | 0.004* | 12.97 (-21.39) |
|  |  | Triglycerides | Ref | 1.14 (0.96 - 1.35) | 1.00 (0.84 - 1.20) | 1.31 (1.10 - 1.55) | 1.10 (1.03 - 1.17) | 0.003* | 13.28 (-19.52) |
|  |  | HbA1c | Ref | 1.14 (0.96 - 1.35) | 1.01 (0.84 - 1.21) | 1.31 (1.10 - 1.56) | 1.10 (1.03 - 1.17) | 0.003* | 13.43 (-18.63) |
| Non-free sugars | | |  |  |  |  |  |  |  |
|  | Mean intake (SD), % of energy | | 6.80 (1.61) | 10.67 (0.92) | 14.07 (1.10) | 20.51 (4.09) |  |  |  |
|  | Cases, n (%) | | 293 | 277 | 274 | 280 | 1124 |  |  |
|  | Minimally adjusted model^d^ | | Ref | 0.86 (0.73 - 1.01) | 0.82 (0.70 - 0.98) | 0.83 (0.70 - 0.98) | 0.94 (0.88 - 0.99) | 0.025 | 6.58 (Ref) |
|  | Multivariable adjusted model^e^ | | Ref | 0.90 (0.76 - 1.06) | 0.86 (0.72 - 1.03) | 0.83 (0.69 - 1.00) | 0.93 (0.87 - 0.99) | 0.027 | 4.17 (-36.60) |
|  | Multivariable adjusted model with single adjustment for^f^ | | |  |  |  |  |  |  |
|  |  | BMI (final model) | Ref | 0.91 (0.77 - 1.07) | 0.87 (0.73 - 1.04) | 0.84 (0.69 - 1.01) | 0.93 (0.88 - 0.99) | 0.033 | 3.69 (-43.84) |
|  |  | Waist circumference | Ref | 0.91 (0.77 - 1.07) | 0.87 (0.73 - 1.04) | 0.84 (0.70 - 1.02) | 0.94 (0.88 - 1.00) | 0.039 | 3.47 (-47.26) |
|  |  | SBP | Ref | 0.90 (0.76 - 1.07) | 0.87 (0.73 - 1.03) | 0.84 (0.69 - 1.01) | 0.93 (0.88 - 1.00) | 0.035 | 3.79 (-42.42) |
|  |  | LDL-C | Ref | 0.90 (0.76 - 1.06) | 0.86 (0.72 - 1.02) | 0.83 (0.69 - 1.00) | 0.93 (0.87 - 0.99) | 0.025 | 4.33 (-34.21) |
|  |  | ApoB | Ref | 0.90 (0.76 - 1.06) | 0.86 (0.72 - 1.02) | 0.83 (0.69 - 1.00) | 0.93 (0.87 - 0.99) | 0.027 | 4.20 (-36.20) |
|  |  | HDL-C | Ref | 0.90 (0.76 - 1.06) | 0.86 (0.72 - 1.03) | 0.83 (0.69 - 1.00) | 0.93 (0.87 - 0.99) | 0.026 | 4.22 (-35.78) |
|  |  | Triglycerides | Ref | 0.90 (0.76 - 1.06) | 0.86 (0.72 - 1.03) | 0.83 (0.69 - 1.01) | 0.93 (0.87 - 0.99) | 0.028 | 4.13 (-37.25) |
|  |  | HbA1c | Ref | 0.90 (0.76 - 1.07) | 0.87 (0.73 - 1.03) | 0.84 (0.69 - 1.01) | 0.93 (0.88 - 0.99) | 0.032 | 3.86 (-41.25) |
| Fibre | | |  |  |  |  |  |  |  |
|  | Mean intake (SD), g/d | | 11.34 (2.07) | 11.34 (2.07) | 15.69 (0.95) | 19.11 (1.08) |  |  |  |
|  | Cases, n (%) | | 279 | 274 | 269 | 302 | 1124 |  |  |
|  | Minimally adjusted model^d^ | | Ref | 0.90 (0.77 - 1.07) | 0.84 (0.71 - 1.00) | 0.90 (0.76 - 1.05) | 0.95 (0.90 - 1.00) | 0.059 | 4.05 (Ref) |
|  | Multivariable adjusted model^e^ | | Ref | 0.93 (0.79 - 1.11) | 0.88 (0.73 - 1.06) | 0.92 (0.75 - 1.13) | 0.95 (0.89 - 1.01) | 0.12 | 1.86 (-54.15) |
|  | Multivariable adjusted model with single adjustment for^f^ | | |  |  |  |  |  |  |
|  |  | BMI (final model) | Ref | 0.94 (0.79 - 1.12) | 0.89 (0.74 - 1.07) | 0.94 (0.77 - 1.15) | 0.96 (0.89 - 1.02) | 0.19 | 1.51 (-62.64) |
|  |  | Waist circumference | Ref | 0.94 (0.79 - 1.12) | 0.89 (0.74 - 1.07) | 0.94 (0.77 - 1.15) | 0.96 (0.89 - 1.02) | 0.20 | 1.49 (-63.17) |
|  |  | SBP | Ref | 0.94 (0.79 - 1.12) | 0.89 (0.74 - 1.07) | 0.94 (0.77 - 1.15) | 0.96 (0.89 - 1.02) | 0.18 | 1.54 (-61.90) |
|  |  | LDL-C | Ref | 0.93 (0.79 - 1.11) | 0.88 (0.73 - 1.06) | 0.92 (0.75 - 1.12) | 0.95 (0.89 - 1.01) | 0.11 | 1.91 (-52.89) |
|  |  | ApoB | Ref | 0.94 (0.79 - 1.11) | 0.88 (0.73 - 1.06) | 0.92 (0.75 - 1.13) | 0.95 (0.89 - 1.01) | 0.12 | 1.81 (-55.30) |
|  |  | HDL-C | Ref | 0.93 (0.78 - 1.11) | 0.88 (0.73 - 1.06) | 0.92 (0.75 - 1.13) | 0.95 (0.89 - 1.01) | 0.12 | 1.84 (-54.50) |
|  |  | Triglycerides | Ref | 0.93 (0.78 - 1.11) | 0.88 (0.73 - 1.06) | 0.92 (0.75 - 1.13) | 0.95 (0.89 - 1.01) | 0.12 | 1.83 (-54.79) |
|  |  | HbA1c | Ref | 0.93 (0.78 - 1.11) | 0.88 (0.73 - 1.06) | 0.93 (0.76 - 1.13) | 0.95 (0.89 - 1.02) | 0.13 | 1.86 (-54.09) |
| ^a^ Carbohydrate intakes were expressed for each 5% of energy, excepting for fibre expressed for each 5 g/d.  ^b^ *P*-trend using continuous intakes with asterisks indicating statistical significance after using false discovery rate to correct for multiple testing. *P*-trend values ≥0.1 are displayed to two decimal places and *P*-trend values <0.1 are displayed to three decimal places.  ^c^ χ2 value for likelihood ratio test comparing model with and without the exposure and percentage change in χ2 value calculated with the minimally adjusted model as the reference group.  ^d^ Minimally adjusted model stratified by age at recruitment and sex and adjusted for recruitment region.  ^e^ Minimally adjusted model with additional adjustment for ethnicity, Townsend deprivation index, education, alcohol intake, smoking status, physical activity, menopausal status, SFA intake, and daily energy intake. Models were also adjusted for fruit and vegetable intake, excepting for models with total sugars, non-free sugars, and fibre as the exposure.  ^f^ Multivariable adjusted model with single adjustment for one of the following: BMI, waist circumference, SBP, LDL-C, ApoB, Triglycerides, HDL-C, or HbA1c. Models adjusting for LDL-C or ApoB were also adjusted for statin use. Full details for each covariate are provided in the statistical analysis section in the main text.  *Abbreviations: Apo* apolipoprotein, *BMI* body mass index, *CI* confidence intervals, *g/d* grams per day, *HbA1c* glycated haemoglobin, *HDL-C* high-density lipoprotein cholesterol, HR hazard ratio, *LDL-C* low-density lipoprotein cholesterol, *Q* quartile, *Ref* reference, *SBP* systolic blood pressure, *SFA* saturated fatty acid. | | | | | | | | | |

#### **Table S13.** Hazard ratios (95% confidence intervals) for the associations between carbohydrate intakes and total CVD, IHD and total stroke risk in sensitivity analyses restricting to participants with ≥ three 24-hour dietary assessments (n=67,218).

| **Carbohydrate intakes** | | **Hazard ratio (95% CI)** | | | | |
| --- | --- | --- | --- | --- | --- | --- |
|  |  | **Total CVD** |  | **IHD** |  | **Total stroke** |
| Total N | | 67216 |  | 67216 |  | 67216 |
| N cases | | 2445 |  | 1793 |  | 685 |
| Total carbohydrate | |  |  |  |  |  |
|  | For each 5% of energy | 1.02 (0.98 - 1.05) |  | 1.03 (0.99 - 1.08) |  | 0.98 (0.92 - 1.05) |
|  | *P*-trend^a^ | 0.31 |  | 0.12 |  | 0.60 |
| Refined grain starch | |  |  |  |  |  |
|  | For each 5% of energy | 1.00 (0.96 - 1.04) |  | 1.00 (0.96 - 1.05) |  | 1.00 (0.93 - 1.07) |
|  | *P*-trend^a^ | 0.96 |  | 0.94 |  | 0.92 |
| Wholegrain starch | |  |  |  |  |  |
|  | For each 5% of energy | 0.99 (0.94 - 1.05) |  | 1.02 (0.96 - 1.08) |  | 0.93 (0.84 - 1.03) |
|  | *P*-trend^a^ | 0.78 |  | 0.54 |  | 0.14 |
| Total sugars | |  |  |  |  |  |
|  | For each 5% of energy | 1.02 (0.98 - 1.05) |  | 1.02 (0.98 - 1.06) |  | 1.01 (0.95 - 1.08) |
|  | *P*-trend^a^ | 0.38 |  | 0.39 |  | 0.68 |
| Free sugars | |  |  |  |  |  |
|  | For each 5% of energy | 1.06 (1.01 - 1.11) |  | 1.04 (0.99 - 1.10) |  | 1.11 (1.02 - 1.21) |
|  | *P*-trend^a^ | 0.012 |  | 0.12 |  | 0.011 |
| Non-free sugars | |  |  |  |  |  |
|  | For each 5% of energy | 0.97 (0.93 - 1.01) |  | 0.99 (0.94 - 1.04) |  | 0.92 (0.84 - 1.00) |
|  | *P*-trend^a^ | 0.16 |  | 0.66 |  | 0.045 |
| Fibre | |  |  |  |  |  |
|  | For each 5 g/d | 0.97 (0.92 - 1.01) |  | 0.99 (0.94 - 1.05) |  | 0.92 (0.84 - 1.00) |
|  | *P*-trend^a^ | 0.15 |  | 0.74 |  | 0.054 |
| Models stratified by age at recruitment and sex, and adjusted for recruitment region, ethnicity, Townsend deprivation index, education, alcohol intake, smoking status, physical activity, menopausal status, BMI, SBP, SFA intake, and daily energy intake. Models were also adjusted for fruit and vegetable intake, excepting for models with total sugars, non-free sugars, and fibre as the exposure. Full details for each covariate are provided in the statistical analysis section in the main text.  ^a^ *P*-trend using continuous intakes with asterisks indicating statistical significance after using false discovery rate to correct for multiple testing. *P*-trend values ≥0.1 are displayed to two decimal places and *P*-trend values <0.1 are displayed to three decimal places.  *Abbreviations: BMI* body mass index, *CI* confidence intervals, *CVD* cardiovascular disease, *HR* hazard ratio, *IHD* ischaemic heart disease, *SBP* systolic blood pressure, *SFA* saturated fatty acid. | | | | | | |

#### **Table S14.** Hazard ratios (95% confidence intervals) for the associations between carbohydrate intakes and total CVD, IHD and total stroke risk in sensitivity analyses restricting to participants with ≥ two years of follow-up (n=109,682).

| **Carbohydrate intakes** | | **Hazard ratio (95% CI)** | | | | |
| --- | --- | --- | --- | --- | --- | --- |
|  |  | **Total CVD** |  | **IHD** |  | **Total stroke** |
| Total N | | 109682 |  | 109682 |  | 109682 |
| N cases | | 3479 |  | 2579 |  | 957 |
| Total carbohydrate | |  |  |  |  |  |
|  | For each 5% of energy | 1.02 (0.99 - 1.05) |  | 1.02 (0.99 - 1.05) |  | 1.01 (0.96 - 1.06) |
|  | *P*-trend^a^ | 0.21 |  | 0.21 |  | 0.74 |
| Refined grain starch | |  |  |  |  |  |
|  | For each 5% of energy | 1.00 (0.97 - 1.03) |  | 1.00 (0.97 - 1.04) |  | 1.00 (0.94 - 1.05) |
|  | *P*-trend^a^ | 0.88 |  | 0.96 |  | 0.88 |
| Wholegrain starch | |  |  |  |  |  |
|  | For each 5% of energy | 0.98 (0.94 - 1.02) |  | 0.99 (0.94 - 1.04) |  | 0.96 (0.88 - 1.04) |
|  | *P*-trend^a^ | 0.40 |  | 0.57 |  | 0.31 |
| Total sugars | |  |  |  |  |  |
|  | For each 5% of energy | 1.03 (1.00 - 1.06) |  | 1.03 (0.99 - 1.06) |  | 1.03 (0.98 - 1.09) |
|  | *P*-trend^a^ | 0.07 |  | 0.13 |  | 0.29 |
| Free sugars | |  |  |  |  |  |
|  | For each 5% of energy | 1.06 (1.02 - 1.10) |  | 1.04 (1.00 - 1.09) |  | 1.11 (1.04 - 1.19) |
|  | *P*-trend^a^ | 0.001* |  | 0.041 |  | 0.002* |
| Non-free sugars | |  |  |  |  |  |
|  | For each 5% of energy | 0.98 (0.94 - 1.01) |  | 0.99 (0.95 - 1.04) |  | 0.93 (0.87 - 1.00) |
|  | *P*-trend^a^ | 0.22 |  | 0.75 |  | 0.037 |
| Fibre | |  |  |  |  |  |
|  | For each 5 g/d | 0.96 (0.93 - 1.00) |  | 0.97 (0.93 - 1.01) |  | 0.93 (0.87 - 1.01) |
|  | *P*-trend^a^ | 0.043 |  | 0.16 |  | 0.069 |
| Models stratified by age at recruitment and sex, and adjusted for recruitment region, ethnicity, Townsend deprivation index, education, alcohol intake, smoking status, physical activity, menopausal status, BMI, SBP, SFA intake, and daily energy intake. Models were also adjusted for fruit and vegetable intake, excepting for models with total sugars, non-free sugars, and fibre as the exposure. Full details for each covariate are provided in the statistical analysis section in the main text.  ^a^ *P*-trend using continuous intakes with asterisks indicating statistical significance after using false discovery rate to correct for multiple testing. *P*-trend values ≥0.1 are displayed to two decimal places and *P*-trend values <0.1 are displayed to three decimal places.  *Abbreviations: BMI* body mass index, *CI* confidence intervals, *CVD* cardiovascular disease, *HR* hazard ratio, *IHD* ischaemic heart disease, *SBP* systolic blood pressure, *SFA* saturated fatty acid. | | | | | | |

#### **Table S15.** Hazard ratios (95% confidence intervals) for the associations between intake of refined grain foods and wholegrain foods in grams and total CVD, IHD and total stroke risk (n=110,497).

| **Carbohydrate intakes** | | **Hazard ratio (95% CI)** | | | | |
| --- | --- | --- | --- | --- | --- | --- |
|  |  | **Total CVD** |  | **IHD** |  | **Total stroke** |
| Total N | | 110497 |  | 110497 |  | 110497 |
| N cases | | 4188 |  | 3138 |  | 1124 |
| Refined grain foods^b^ | |  |  |  |  |  |
|  | For each 30 g/d | 0.99 (0.98 - 1.00) |  | 0.99 (0.98 - 1.01) |  | 0.98 (0.96 - 1.01) |
|  | *P*-trend^a^ | 0.048 |  | 0.23 |  | 0.18 |
| Wholegrain foods^c^ | |  |  |  |  |  |
|  | For each 30 g/d | 0.99 (0.98 - 1.00) |  | 0.99 (0.98 - 1.01) |  | 0.99 (0.96 - 1.01) |
|  | *P*-trend^a^ | 0.22 |  | 0.38 |  | 0.29 |
| Models stratified by age at recruitment and sex, and adjusted for recruitment region, ethnicity, Townsend deprivation index, education, alcohol intake, smoking status, physical activity, menopausal status, BMI, SBP, SFA intake, fruit and vegetable intake, and daily energy intake. Full details for each covariate are provided in the statistical analysis section in the main text.  ^a^ *P*-trend using continuous intakes with asterisks indicating statistical significance after using false discovery rate to correct for multiple testing. *P*-trend values ≥0.1 are displayed to two decimal places and *P*-trend values <0.1 are displayed to three decimal places.  ^b^ Calculated as the total food weight consumed from white bread, other bread (e.g. naan, garlic bread), other cereal (sugar) (e.g. Kellogg’s Cornflakes cereal), white pasta & rice, pizza, samosa, pakora, grain dishes (added fat), savoury snacks, biscuits, other desserts & cakes & pastries, and savoury crackers.  ^c^ Calculated as the total food weight consumed from wholegrain flour, including wholemeal bread, bran cereal (e.g. bran flakes, All Bran), biscuit cereal (e.g. Weetabix, Shredded Wheat, Shreddies), oat cereal (non-sugar), oat cereal (sugar), muesli, wholemeal pasta, brown rice & other wholegrains.  *Abbreviations: BMI* body mass index, *CI* confidence intervals, *CVD* cardiovascular disease, *HR* hazard ratio, *IHD* ischaemic heart disease, *SBP* systolic blood pressure, *SFA* saturated fatty acid. | | | | | | |

#### **Table S16.** Hazard ratios (95% confidence intervals) for the associations between types of carbohydrate and total CVD, IHD and total stroke risk in 110,497 UK Biobank participants by sex subgroups.

| **Carbohydrate intakes** | | **Hazard ratio (95% CI)** | | | | | | | | | | |
| --- | --- | --- | --- | --- | --- | --- | --- | --- | --- | --- | --- | --- |
|  |  | **Total CVD** | | |  | **IHD** | | |  | **Total stroke** | | |
|  |  | **Women** |  | **Men** |  | **Women** |  | **Men** |  | **Women** |  | **Men** |
| Total N | | 63974 |  | 46523 |  | 63974 |  | 46523 |  | 63974 |  | 46523 |
| N cases | | 1456 |  | 2732 |  | 953 |  | 2185 |  | 534 |  | 590 |
| Total carbohydrate | |  |  |  |  |  |  |  |  |  |  |  |
|  | For each 5% of energy | 1.03 (0.99 - 1.08) |  | 1.01 (0.98 - 1.04) |  | 1.07 (1.01 - 1.13) |  | 1.01 (0.97 - 1.04) |  | 0.97 (0.90 - 1.04) |  | 1.02 (0.95 - 1.09) |
|  | *P*-trend^a^ | 0.17 |  | 0.50 |  | 0.025 |  | 0.61 |  | 0.39 |  | 0.57 |
|  | Test of difference between subgroups^b^ | χ2=4.52, p=0.21 | | |  | χ2=7.70, p=0.053 | | |  | χ2=3.98, p=0.26 | | |
| Refined grain starch | |  |  |  |  |  |  |  |  |  |  |  |
|  | For each 5% of energy | 0.98 (0.93 - 1.02) |  | 1.00 (0.97 - 1.03) |  | 0.99 (0.94 - 1.05) |  | 1.00 (0.96 - 1.04) |  | 0.95 (0.88 - 1.02) |  | 1.01 (0.94 - 1.08) |
|  | *P*-trend^a^ | 0.32 |  | 0.92 |  | 0.77 |  | 0.99 |  | 0.18 |  | 0.79 |
|  | Test of difference between subgroups^b^ | χ2=2.90, p=0.41 | | |  | χ2=3.45, p=0.33 | | |  | χ2=1.93, p=0.59 | | |
| Wholegrain starch | |  |  |  |  |  |  |  |  |  |  |  |
|  | For each 5% of energy | 1.02 (0.95 - 1.09) |  | 0.96 (0.92 - 1.01) |  | 1.06 (0.97 - 1.15) |  | 0.96 (0.91 - 1.01) |  | 0.95 (0.85 - 1.07) |  | 0.98 (0.89 - 1.08) |
|  | *P*-trend^a^ | 0.53 |  | 0.123 |  | 0.18 |  | 0.091* |  | 0.40 |  | 0.70 |
|  | Test of difference between subgroups^b^ | χ2=6.02, p=0.11 | | |  | χ2=10.31, p=0.016 | | |  | χ2=0.42, p=0.94 | | |
| Total sugars | |  |  |  |  |  |  |  |  |  |  |  |
|  | For each 5% of energy | 1.02 (0.98 - 1.07) |  | 1.03 (1.00 - 1.06) |  | 1.02 (0.96 - 1.07) |  | 1.03 (1.00 - 1.07) |  | 1.04 (0.97 - 1.11) |  | 1.01 (0.94 - 1.09) |
|  | *P*-trend^a^ | 0.29 |  | 0.082 |  | 0.54 |  | 0.072 |  | 0.30 |  | 0.73 |
|  | Test of difference between subgroups^b^ | χ2=1.17, p=0.76 | | |  | χ2=4.16, p=0.24 | | |  | χ2=2.84, p=0.42 | | |
| Free sugars | |  |  |  |  |  |  |  |  |  |  |  |
|  | For each 5% of energy | 1.08 (1.02 - 1.15) |  | 1.06 (1.02 - 1.11) |  | 1.04 (0.97 - 1.12) |  | 1.07 (1.02 - 1.11) |  | 1.15 (1.05 - 1.26) |  | 1.06 (0.98 - 1.16) |
|  | *P*-trend^a^ | 0.006* |  | 0.002* |  | 0.26 |  | 0.003* |  | 0.002* |  | 0.15 |
|  | Test of difference between subgroups^b^ | χ2=0.20, p=0.98 | | |  | χ2=1.17, p=0.76 | | |  | χ2=0.50, p=0.92 | | |
| Non-free sugars | |  |  |  |  |  |  |  |  |  |  |  |
|  | For each 5% of energy | 0.96 (0.91 - 1.02) |  | 0.97 (0.93 - 1.01) |  | 0.99 (0.92 - 1.05) |  | 0.98 (0.93 - 1.02) |  | 0.93 (0.86 - 1.02) |  | 0.93 (0.85 - 1.03) |
|  | *P*-trend^a^ | 0.17 |  | 0.177 |  | 0.65 |  | 0.34 |  | 0.12 |  | 0.15 |
|  | Test of difference between subgroups^b^ | χ2=0.55, p=0.91 | | |  | χ2=1.70, p=0.64 | | |  | χ2=2.31, p=0.51 | | |
| Fibre | |  |  |  |  |  |  |  |  |  |  |  |
|  | For each 5 g/d | 1.00 (0.94 - 1.06) |  | 0.94 (0.90 - 0.98) |  | 1.02 (0.94 - 1.10) |  | 0.94 (0.89 - 0.98) |  | 0.97 (0.87 - 1.07) |  | 0.94 (0.86 - 1.03) |
|  | *P*-trend^a^ | 0.97 |  | 0.003* |  | 0.63 |  | 0.005* |  | 0.55 |  | 0.20 |
|  | Test of difference between subgroups^b^ | χ2=5.39, p=0.15 | | |  | χ2=8.15, p=0.043 | | |  | χ2=2.11, p=0.55 | | |
| Models stratified by age at recruitment, and adjusted for recruitment region, ethnicity, Townsend deprivation index, education, alcohol intake, smoking status, physical activity, menopausal status, BMI, SBP, SFA intake, and daily energy intake. Models were also adjusted for fruit and vegetable intake, excepting for models with total sugars, non-free sugars, and fibre as the exposure. Full details for each covariate are provided in the statistical analysis section in the main text.  *P*-values ≥0.1 are displayed to two decimal places and *P*-values <0.1 are displayed to three decimal places.  ^a^ *P*-trend using continuous intakes with asterisks indicating statistical significance after using false discovery rate to correct for multiple testing.  ^b^ Tests of difference between subgroups were performed by adding appropriate interaction terms to the multivariable Cox models and testing for statistical significance of interaction across strata using likelihood ratio tests with asterisks indicating statistical significance after using false discovery rate to correct for multiple testing.  *Abbreviations: BMI* body mass index, *CI* confidence intervals, *CVD* cardiovascular disease, *HR* hazard ratio, *IHD* ischaemic heart disease, *SBP* systolic blood pressure, *SFA* saturated fatty acid. | | | | | | | | | | | | |

#### **Table S17.** Hazard ratios (95% confidence intervals) for the associations between types of carbohydrate and total CVD, IHD and total stroke risk in 110,497 UK Biobank participants by BMI subgroups.

| **Carbohydrate intakes** | | **Hazard ratio (95% CI)** | | | | | | | | | | |
| --- | --- | --- | --- | --- | --- | --- | --- | --- | --- | --- | --- | --- |
|  |  | **Total CVD** | | |  | **IHD** | | |  | **Total stroke** | | |
|  |  | **BMI <26 kg/m^2^** |  | **BMI ≥26 kg/m^2^** |  | **BMI <26 kg/m^2^** |  | **BMI ≥26 kg/m^2^** |  | **BMI <26 kg/m^2^** |  | **BMI ≥26 kg/m^2^** |
| Total N | | 56997 |  | 53298 |  | 56997 |  | 53298 |  | 56997 |  | 53298 |
| N cases | | 1703 |  | 2473 |  | 1226 |  | 1904 |  | 504 |  | 616 |
| Total carbohydrate | |  |  |  |  |  |  |  |  |  |  |  |
|  | For each 5% of energy | 1.00 (0.96 - 1.05) |  | 1.02 (0.99 - 1.05) |  | 1.01 (0.97 - 1.06) |  | 1.02 (0.99 - 1.06) |  | 0.98 (0.91 - 1.06) |  | 1.00 (0.94 - 1.07) |
|  | *P*-trend^a^ | 0.89 |  | 0.26 |  | 0.58 |  | 0.23 |  | 0.65 |  | 0.98 |
|  | Test of difference between subgroups^b^ | χ2=0.10, p=0.99 | | |  | χ2=0.29, p=0.96 | | |  | χ2=0.62, p=0.89 | | |
| Refined grain starch | |  |  |  |  |  |  |  |  |  |  |  |
|  | For each 5% of energy | 0.97 (0.93 - 1.02) |  | 1.00 (0.97 - 1.04) |  | 0.97 (0.92 - 1.02) |  | 1.01 (0.98 - 1.05) |  | 0.97 (0.90 - 1.05) |  | 0.99 (0.92 - 1.06) |
|  | *P*-trend^a^ | 0.21 |  | 0.86 |  | 0.26 |  | 0.50 |  | 0.48 |  | 0.71 |
|  | Test of difference between subgroups^b^ | χ2=3.07, p=0.38 | | |  | χ2=3.17, p=0.37 | | |  | χ2=0.69, p=0.88 | | |
| Wholegrain starch | |  |  |  |  |  |  |  |  |  |  |  |
|  | For each 5% of energy | 0.98 (0.93 - 1.04) |  | 0.97 (0.93 - 1.03) |  | 0.96 (0.90 - 1.03) |  | 0.99 (0.93 - 1.05) |  | 1.03 (0.93 - 1.15) |  | 0.91 (0.82 - 1.02) |
|  | *P*-trend^a^ | 0.53 |  | 0.32 |  | 0.26 |  | 0.72 |  | 0.55 |  | 0.092 |
|  | Test of difference between subgroups^b^ | χ2=0.26, p=0.97 | | |  | χ2=0.72, p=0.87 | | |  | χ2=3.37, p=0.338 | | |
| Total sugars | |  |  |  |  |  |  |  |  |  |  |  |
|  | For each 5% of energy | 1.04 (1.00 - 1.08) |  | 1.01 (0.98 - 1.05) |  | 1.04 (0.99 - 1.10) |  | 1.01 (0.98 - 1.05) |  | 1.04 (0.96 - 1.12) |  | 1.01 (0.94 - 1.08) |
|  | *P*-trend^a^ | 0.066 |  | 0.45 |  | 0.079 |  | 0.52 |  | 0.33 |  | 0.82 |
|  | Test of difference between subgroups^b^ | χ2=1.36, p=0.71 | | |  | χ2=1.92, p=0.59 | | |  | χ2=2.31, p=0.51 | | |
| Free sugars | |  |  |  |  |  |  |  |  |  |  |  |
|  | For each 5% of energy | 1.09 (1.04 - 1.14) |  | 1.05 (1.01 - 1.09) |  | 1.08 (1.02 - 1.15) |  | 1.03 (0.99 - 1.08) |  | 1.10 (1.01 - 1.21) |  | 1.10 (1.01 - 1.19) |
|  | *P*-trend^a^ | 0.001* |  | 0.026 |  | 0.008 |  | 0.16 |  | 0.038 |  | 0.028 |
|  | Test of difference between subgroups^b^ | χ2=2.29, p=0.51 | | |  | χ2=2.84, p=0.42 | | |  | χ2=0.79, p=0.85 | | |
| Non-free sugars | |  |  |  |  |  |  |  |  |  |  |  |
|  | For each 5% of energy | 0.97 (0.92 - 1.03) |  | 0.96 (0.92 - 1.01) |  | 0.98 (0.92 - 1.04) |  | 0.98 (0.93 - 1.03) |  | 0.97 (0.88 - 1.06) |  | 0.90 (0.83 - 0.98) |
|  | *P*-trend^a^ | 0.32 |  | 0.100 |  | 0.55 |  | 0.43 |  | 0.51 |  | 0.021 |
|  | Test of difference between subgroups^b^ | χ2=7.05, p=0.070 | | |  | χ2=1.35, p=0.72 | | |  | χ2=8.78, p=0.032 | | |
| Fibre | |  |  |  |  |  |  |  |  |  |  |  |
|  | For each 5 g/d | 0.95 (0.90 - 1.00) |  | 0.96 (0.92 - 1.00) |  | 0.93 (0.88 - 1.00) |  | 0.97 (0.92 - 1.02) |  | 0.99 (0.90 - 1.09) |  | 0.92 (0.84 - 1.01) |
|  | *P*-trend^a^ | 0.052 |  | 0.066 |  | 0.035 |  | 0.22 |  | 0.85 |  | 0.086 |
|  | Test of difference between subgroups^b^ | χ2=0.32, p=0.96 | | |  | χ2=0.77, p=0.86 | | |  | χ2=2.13, p=0.55 | | |
| Models stratified by age at recruitment and sex, and adjusted for recruitment region, ethnicity, Townsend deprivation index, education, alcohol intake, smoking status, physical activity, menopausal status, SBP, SFA intake, and daily energy intake. Models were also adjusted for fruit and vegetable intake, excepting for models with total sugars, non-free sugars, and fibre as the exposure. Full details for each covariate are provided in the statistical analysis section in the main text.  *P*-values ≥0.1 are displayed to two decimal places and *P*-values <0.1 are displayed to three decimal places.  ^a^ *P*-trend using continuous intakes with asterisks indicating statistical significance after using false discovery rate to correct for multiple testing.  ^b^ Tests of difference between subgroups were performed by adding appropriate interaction terms to the multivariable Cox models and testing for statistical significance of interaction across strata using likelihood ratio tests with asterisks indicating statistical significance after using false discovery rate to correct for multiple testing.  *Abbreviations: BMI* body mass index, *CI* confidence intervals, *CVD* cardiovascular disease, *HR* hazard ratio, *IHD* ischaemic heart disease, *SBP* systolic blood pressure, *SFA* saturated fatty acid. | | | | | | | | | | | | |

#### **Table S18.** Hazard ratios (95% confidence intervals) for the associations between types of carbohydrate and total CVD, IHD and total stroke risk in 110,497 UK Biobank participants by smoking status subgroups.

| **Carbohydrate intakes** | | **Hazard ratio (95% CI)** | | | | | | | | | | |
| --- | --- | --- | --- | --- | --- | --- | --- | --- | --- | --- | --- | --- |
|  |  | **Total CVD** | | |  | **IHD** | | |  | **Total stroke** | | |
|  |  | **Never smoker** |  | **Ever smoker** |  | **Never smoker** |  | **Ever smoker** |  | **Never smoker** |  | **Ever smoker** |
| Total N | | 64632 |  | 45652 |  | 64632 |  | 45652 |  | 64632 |  | 45652 |
| N cases | | 2083 |  | 2096 |  | 1539 |  | 1591 |  | 577 |  | 546 |
| Total carbohydrate | |  |  |  |  |  |  |  |  |  |  |  |
|  | For each 5% of energy | 1.02 (0.98 - 1.06) |  | 1.01 (0.98 - 1.05) |  | 1.02 (0.98 - 1.07) |  | 1.02 (0.98 - 1.06) |  | 1.02 (0.95 - 1.09) |  | 0.97 (0.91 - 1.04) |
|  | *P*-trend^a^ | 0.30 |  | 0.52 |  | 0.30 |  | 0.28 |  | 0.67 |  | 0.45 |
|  | Test of difference between subgroups^b^ | χ2=2.39, p=0.50 | | |  | χ2=3.01, p=0.39 | | |  | χ2=0.26, p=0.97 | | |
| Refined grain starch | |  |  |  |  |  |  |  |  |  |  |  |
|  | For each 5% of energy | 0.98 (0.94 - 1.02) |  | 1.00 (0.96 - 1.04) |  | 0.98 (0.94 - 1.03) |  | 1.01 (0.97 - 1.05) |  | 0.98 (0.91 - 1.06) |  | 0.98 (0.91 - 1.05) |
|  | *P*-trend^a^ | 0.30 |  | 0.99 |  | 0.41 |  | 0.65 |  | 0.66 |  | 0.51 |
|  | Test of difference between subgroups^b^ | χ2=4.29, p=0.23 | | |  | χ2=4.72, p=0.19 | | |  | χ2=0.96, p=0.81 | | |
| Wholegrain starch | |  |  |  |  |  |  |  |  |  |  |  |
|  | For each 5% of energy | 1.01 (0.96 - 1.07) |  | 0.94 (0.89 - 0.99) |  | 1.03 (0.97 - 1.10) |  | 0.92 (0.86 - 0.98) |  | 0.94 (0.85 - 1.05) |  | 0.99 (0.89 - 1.10) |
|  | *P*-trend^a^ | 0.63 |  | 0.028 |  | 0.28 |  | 0.013* |  | 0.29 |  | 0.80 |
|  | Test of difference between subgroups^b^ | χ2=4.10, p=0.25 | | |  | χ2=6.10, p=0.11 | | |  | χ2=2.72, p=0.44 | | |
| Total sugars | |  |  |  |  |  |  |  |  |  |  |  |
|  | For each 5% of energy | 1.03 (0.99 - 1.07) |  | 1.02 (0.99 - 1.06) |  | 1.02 (0.98 - 1.07) |  | 1.03 (0.99 - 1.07) |  | 1.07 (1.00 - 1.14) |  | 0.98 (0.92 - 1.05) |
|  | *P*-trend^a^ | 0.10 |  | 0.26 |  | 0.31 |  | 0.14 |  | 0.068 |  | 0.63 |
|  | Test of difference between subgroups^b^ | χ2=1.47, p=0.69 | | |  | χ2=6.60, p=0.09 | | |  | χ2=4.55, p=0.21 | | |
| Free sugars | |  |  |  |  |  |  |  |  |  |  |  |
|  | For each 5% of energy | 1.08 (1.03 - 1.14) |  | 1.07 (1.02 - 1.12) |  | 1.06 (1.00 - 1.11) |  | 1.08 (1.02 - 1.13) |  | 1.19 (1.09 - 1.30) |  | 1.04 (0.95 - 1.14) |
|  | *P*-trend^a^ | 0.001* |  | 0.002* |  | 0.052 |  | 0.004* |  | <0.001* |  | 0.36* |
|  | Test of difference between subgroups^b^ | χ2=1.82, p=0.61 | | |  | χ2=2.71, p=0.44 | | |  | χ2=2.86, p=0.41 | | |
| Non-free sugars | |  |  |  |  |  |  |  |  |  |  |  |
|  | For each 5% of energy | 0.96 (0.92 - 1.01) |  | 0.96 (0.91 - 1.00) |  | 0.97 (0.92 - 1.03) |  | 0.97 (0.92 - 1.02) |  | 0.93 (0.85 - 1.01) |  | 0.92 (0.84 - 1.01) |
|  | *P*-trend^a^ | 0.082 |  | 0.063 |  | 0.29 |  | 0.25 |  | 0.090 |  | 0.074 |
|  | Test of difference between subgroups^b^ | χ2=3.20, p=0.36 | | |  | χ2=2.46, p=0.48 | | |  | χ2=1.30, p=0.73 | | |
| Fibre | |  |  |  |  |  |  |  |  |  |  |  |
|  | For each 5 g/d | 0.95 (0.90 - 1.00) |  | 0.94 (0.90 - 0.99) |  | 0.95 (0.89 - 1.00) |  | 0.95 (0.90 - 1.00) |  | 0.95 (0.87 - 1.05) |  | 0.93 (0.85 - 1.03) |
|  | *P*-trend^a^ | 0.043 |  | 0.011* |  | 0.062 |  | 0.044 |  | 0.31 |  | 0.15 |
|  | Test of difference between subgroups^b^ | χ2=2.24, p=0.52 | | |  | χ2=7.68, p=0.053 | | |  | χ2=4.84, p=0.18 | | |
| Models stratified by age at recruitment and sex, and adjusted for recruitment region, ethnicity, Townsend deprivation index, education, alcohol intake, physical activity, menopausal status, BMI, SBP, SFA intake, and daily energy intake. Models were also adjusted for fruit and vegetable intake, excepting for models with total sugars, non-free sugars, and fibre as the exposure. Full details for each covariate are provided in the statistical analysis section in the main text.  *P*-values ≥0.1 are displayed to two decimal places and *P*-values <0.1 are displayed to three decimal places.  ^a^ *P*-trend using continuous intakes with asterisks indicating statistical significance after using false discovery rate to correct for multiple testing.  ^b^ Tests of difference between subgroups were performed by adding appropriate interaction terms to the multivariable Cox models and testing for statistical significance of interaction across strata using likelihood ratio tests with asterisks indicating statistical significance after using false discovery rate to correct for multiple testing.  *Abbreviations: BMI* body mass index, *CI* confidence intervals, *CVD* cardiovascular disease, *HR* hazard ratio, *IHD* ischaemic heart disease, *SBP* systolic blood pressure, *SFA* saturated fatty acid. | | | | | | | | | | | | |

#### **Table S19.** Associations between each 5% of energy from free sugars and concentrations of total triglycerides and triglycerides in lipoprotein subclasses in minimally-adjusted linear regression models in 26,095 UK Biobank participants.

| **Triglycerides** | | **Geometric mean (95% CI), mmol/L** | **Percentage difference geometric mean concentrations (95% CI)** |
| --- | --- | --- | --- |
| Total triglycerides | | 1.142 (1.136, 1.148) | 3.32 (2.79, 3.84) |
| Triglycerides in VLDL | | 0.760 (0.755, 0.765) | 4.24 (3.58, 4.90) |
|  | Triglycerides in chylomicrons & extremely large VLDL | 0.058 (0.057, 0.060) | 12.57 (9.93, 15.28) |
|  | Triglycerides in very large VLDL | 0.073 (0.073, 0.074) | 8.59 (7.29, 9.90) |
|  | Triglycerides in large VLDL | 0.135 (0.134, 0.136) | 4.51 (3.76, 5.27) |
|  | Triglycerides in medium VLDL | 0.241 (0.240, 0.243) | 2.93 (2.39, 3.48) |
|  | Triglycerides in small VLDL | 0.141 (0.140, 0.142) | 2.73 (2.25, 3.21) |
|  | Triglycerides in very small VLDL | 0.064 (0.064, 0.064) | 1.88 (1.51, 2.25) |
| Triglycerides in IDL | | 0.095 (0.094, 0.095) | 1.24 (0.94, 1.55) |
| Triglycerides in LDL | | 0.136 (0.136, 0.137) | 1.40 (1.08, 1.72) |
|  | Triglycerides in large LDL | 0.092 (0.092, 0.092) | 1.18 (0.88, 1.48) |
|  | Triglycerides in medium LDL | 0.030 (0.030, 0.030) | 1.65 (1.29, 2.00) |
|  | Triglycerides in small LDL | 0.013 (0.013, 0.013) | 2.30 (1.89, 2.72) |
| Triglycerides in HDL | | 0.134 (0.133, 0.134) | 2.15 (1.75, 2.56) |
|  | Triglycerides in large HDL | 0.028 (0.028, 0.028) | 1.11 (0.62, 1.60) |
|  | Triglycerides in medium HDL | 0.049 (0.049, 0.050) | 2.36 (1.90, 2.81) |
|  | Triglycerides in small HDL | 0.048 (0.047, 0.048) | 2.95 (2.51, 3.38) |
| Minimally-adjusted linear regression models were adjusted for age at recruitment, sex, and recruitment region. Full details for each covariate are provided in the statistical analysis section in the main text. Results are expressed as the percentage difference (95% CI) in triglyceride concentrations per 5% higher energy intake from free sugars and were calculated as follows: (e^β^-1)*100.  *P*-trend values calculated using continuous intakes were all significant after using false discovery rate to correct for multiple testing.  *Abbreviations: BMI* body mass index, *CI* confidence intervals, *HDL* high-density lipoprotein, *IDL* intermediate-density lipoprotein, *LDL* low-density lipoprotein, *mmol/L* millimoles per litre, *NMR* nuclear magnetic resonance, *SBP* systolic blood pressure, *SFA* saturated fatty acid, *VLDL* very low-density lipoprotein. | | | |

#### **Table S20.** Associations between each 5% of energy from free sugars and concentrations of total triglycerides and triglycerides in lipoprotein subclasses in sensitivity analyses restricting to participants fasting for ≥ four hours prior to serum collection (n=11,076).

| **Triglycerides** | | **Geometric mean (95% CI), mmol/L** | **Percentage difference geometric mean concentrations (95% CI)** |
| --- | --- | --- | --- |
| Total triglycerides | | 1.134 (1.125, 1.142) | 3.32 (2.79, 3.84) |
| Triglycerides in VLDL | | 0.753 (0.746, 0.760) | 4.24 (3.58, 4.90) |
|  | Triglycerides in chylomicrons & extremely large VLDL | 0.061 (0.059, 0.063) | 12.57 (9.93, 15.28) |
|  | Triglycerides in very large VLDL | 0.072 (0.071, 0.074) | 8.59 (7.29, 9.90) |
|  | Triglycerides in large VLDL | 0.133 (0.132, 0.135) | 4.51 (3.76, 5.27) |
|  | Triglycerides in medium VLDL | 0.239 (0.237, 0.241) | 2.93 (2.39, 3.48) |
|  | Triglycerides in small VLDL | 0.139 (0.138, 0.140) | 2.73 (2.25, 3.21) |
|  | Triglycerides in very small VLDL | 0.064 (0.064, 0.064) | 1.88 (1.51, 2.25) |
| Triglycerides in IDL | | 0.095 (0.094, 0.095) | 1.24 (0.94, 1.55) |
| Triglycerides in LDL | | 0.136 (0.136, 0.137) | 1.40 (1.08, 1.72) |
|  | Triglycerides in large LDL | 0.092 (0.091, 0.092) | 1.18 (0.88, 1.48) |
|  | Triglycerides in medium LDL | 0.030 (0.030, 0.030) | 1.65 (1.29, 2.00) |
|  | Triglycerides in small LDL | 0.013 (0.013, 0.013) | 2.30 (1.89, 2.72) |
| Triglycerides in HDL | | 0.133 (0.132, 0.133) | 2.15 (1.75, 2.56) |
|  | Triglycerides in large HDL | 0.028 (0.028, 0.028) | 1.11 (0.62, 1.60) |
|  | Triglycerides in medium HDL | 0.049 (0.049, 0.049) | 2.36 (1.90, 2.81) |
|  | Triglycerides in small HDL | 0.047 (0.047, 0.047) | 2.95 (2.51, 3.38) |
| Multivariable linear regression models were adjusted for age at recruitment, sex, recruitment region, ethnicity, Townsend deprivation index, education, alcohol intake, smoking status, physical activity, menopausal status, SBP, BMI, fruit and vegetable intake, SFA intake, daily energy intake, and fasting status. Full details for each covariate are provided in the statistical analysis section in the main text. Results are expressed as the percentage difference (95% CI) in triglyceride concentrations per 5% higher energy intake from free sugars and were calculated as follows: (e^β^-1)*100.  *P*-trend values calculated using continuous intakes were all significant after using false discovery rate to correct for multiple testing.  *Abbreviations: BMI* body mass index, *CI* confidence intervals, *HDL* high-density lipoprotein, *IDL* intermediate-density lipoprotein, *LDL* low-density lipoprotein, *mmol/L* millimoles per litre, *NMR* nuclear magnetic resonance, *SBP* systolic blood pressure, *SFA* saturated fatty acid, *VLDL* very low-density lipoprotein. | | | |

#### **Table S21.** Associations between each 5% of energy from free sugars and concentrations of total triglycerides and triglycerides in lipoprotein subclasses in sensitivity analyses restricting to participants with all triglyceride measurements above the limit of quantification (n=21,865).

| **Triglycerides** | | **Geometric mean (95% CI), mmol/L** | **Percentage difference geometric mean concentrations (95% CI)** |
| --- | --- | --- | --- |
| Total triglycerides | | 1.282 (1.276, 1.288) | 3.32 (2.79, 3.84) |
| Triglycerides in VLDL | | 0.876 (0.871, 0.881) | 4.24 (3.58, 4.90) |
|  | Triglycerides in chylomicrons & extremely large VLDL | 0.081 (0.079, 0.083) | 12.57 (9.93, 15.28) |
|  | Triglycerides in very large VLDL | 0.096 (0.095, 0.097) | 8.59 (7.29, 9.90) |
|  | Triglycerides in large VLDL | 0.157 (0.156, 0.158) | 4.51 (3.76, 5.27) |
|  | Triglycerides in medium VLDL | 0.271 (0.270, 0.273) | 2.93 (2.39, 3.48) |
|  | Triglycerides in small VLDL | 0.157 (0.157, 0.158) | 2.73 (2.25, 3.21) |
|  | Triglycerides in very small VLDL | 0.070 (0.069, 0.070) | 1.88 (1.51, 2.25) |
| Triglycerides in IDL | | 0.100 (0.100, 0.101) | 1.24 (0.94, 1.55) |
| Triglycerides in LDL | | 0.145 (0.145, 0.146) | 1.40 (1.08, 1.72) |
|  | Triglycerides in large LDL | 0.097 (0.097, 0.097) | 1.18 (0.88, 1.48) |
|  | Triglycerides in medium LDL | 0.033 (0.032, 0.033) | 1.65 (1.29, 2.00) |
|  | Triglycerides in small LDL | 0.015 (0.015, 0.015) | 2.30 (1.89, 2.72) |
| Triglycerides in HDL | | 0.145 (0.144, 0.145) | 2.15 (1.75, 2.56) |
|  | Triglycerides in large HDL | 0.030 (0.030, 0.030) | 1.11 (0.62, 1.60) |
|  | Triglycerides in medium HDL | 0.054 (0.054, 0.054) | 2.36 (1.90, 2.81) |
|  | Triglycerides in small HDL | 0.052 (0.052, 0.052) | 2.95 (2.51, 3.38) |
| Multivariable linear regression models were adjusted for age at recruitment, sex, recruitment region, ethnicity, Townsend deprivation index, education, alcohol intake, smoking status, physical activity, menopausal status, SBP, BMI, fruit and vegetable intake, SFA intake, daily energy intake, and fasting status. Full details for each covariate are provided in the statistical analysis section in the main text. Results are expressed as the percentage difference (95% CI) in triglyceride concentrations per 5% higher energy intake from free sugars and were calculated as follows: (e^β^-1)*100.  *P*-trend values calculated using continuous intakes were all significant after using false discovery rate to correct for multiple testing.  *Abbreviations: BMI* body mass index, *CI* confidence intervals, *HDL* high-density lipoprotein, *IDL* intermediate-density lipoprotein, *LDL* low-density lipoprotein, *mmol/L* millimoles per litre, *NMR* nuclear magnetic resonance, *SBP* systolic blood pressure, *SFA* saturated fatty acid, *VLDL* very low-density lipoprotein. | | | |

## Additional File 1: Figures

#### **Fig. S1** Dietary assessments in sub-sample of 110,497 UK biobank participants included in our main analyses.

##### Numbers for 24-hour dietary assessment (WebQ) completion in the main analyses excluded participants who withdrew consent, had prevalent CVD at baseline, or were pregnant or unsure if pregnant at baseline. Participants were also excluded if they did not meet the minimum requirements for ≥ two valid 24-hour dietary assessments after assessments were excluded if participants reported extreme values for total energy intake or if participants reported they were ill or fasting on the respective day. *Abbreviations: CVD* cardiovascular disease.

#### **Fig. S2** Top five food contributors to total carbohydrates and types of carbohydrates in 110,497 UK Biobank participants.

##### *Abbreviations: g/d* grams per day, *SSB* sugar-sweetened beverage.
